# Novel *N*-Substituted Amino Acid Hydrazone-Isatin Derivatives: Synthesis, Antioxidant Activity, and Anticancer Activity in 2D and 3D Models *In Vitro*

**DOI:** 10.3390/ijms22157799

**Published:** 2021-07-21

**Authors:** Ingrida Tumosienė, Ilona Jonuškienė, Kristina Kantminienė, Vytautas Mickevičius, Vilma Petrikaitė

**Affiliations:** 1 Department of Organic Chemistry, Kaunas University of Technology, Radvilėnų pl. 19, LT-50254 Kaunas, Lithuania; ingrida.tumosiene@ktu.lt (I.T.); ilona.jonuskiene@ktu.lt (I.J.); vytautas.mickevicius@ktu.lt (V.M.); 2 Department of Physical and Inorganic Chemistry, Kaunas University of Technology, Radvilėnų pl. 19, LT-50254 Kaunas, Lithuania; 3 Laboratory of Drug Targets Histopathology, Institute of Cardiology, Lithuanian University of Health Sciences, Sukilėlių pr. 13, LT-50162 Kaunas, Lithuania; vilma.petrikaite@lsmuni.lt; 4 Institute of Physiology and Pharmacology, Faculty of Medicine, Lithuanian University of Health Sciences, A. Mickevičiaus g. 9, LT-44307 Kaunas, Lithuania; 5 Life Sciences Center, Institute of Biotechnology, Vilnius University, Saulėtekio al. 7, LT-10257 Vilnius, Lithuania

**Keywords:** hydrazone, isatin, 2-oxindole, pyrrolidin-2-one, colon cancer, melanoma, 3D tumor model, clonogenic

## Abstract

A series of novel mono and bishydrazones each bearing a 2-oxindole moiety along with substituted phenylaminopropanamide, pyrrolidin-2-one, benzimidazole, diphenylmethane, or diphenylamine fragments were synthesized, and their anticancer activities were tested by MTT assay against human melanoma A375 and colon adenocarcinoma HT-29 cell lines. In general, the synthesized compounds were more cytotoxic against HT-29 than A375. 3-((4-Methoxyphenyl)(3-oxo-3-(2-(2-oxoindolin-3-ylidene)hydrazinyl)propyl)amino)-*N*′-(2-oxoindolin-3-ylidene)propanehydrazide and (*N*′,*N*‴)-1,1′-(methylenebis(4,1-phenylene))bis(5-oxo-*N*′-(2-oxoindolin-3-ylidene)pyrrolidine-3-carbohydrazide) were identified as the most active compounds against HT-29 in 2D and 3D cell cultures. The same compounds showed the highest antioxidant activity among the synthesized compounds screened by ferric reducing antioxidant power assay (FRAP). Their antioxidant activity is on par with that of a well-known antioxidant ascorbic acid.

## 1. Introduction

Cancer is a malignant disease characterized by rapid and uncontrolled cell proliferation [1]. The early cancer treatment strategies were based on non-specific chemotherapeutic agents that block DNA synthesis in replicating cells [2], act as antimetabolites [3], or induce cell cycle arrest by other mechanisms [4]. Almost all existing anticancer cytostatic drugs have severe side effects due to low selectivity of the antiproliferative action and allow tumors to develop resistance to multiple chemotherapeutic drugs. The search for new effective anticancer agents with superior selectivity towards cancer cells is still of crucial importance [5]. The more recent strategies based on the targeted therapies aim at the identification and targeting of biomarkers specific for cancer cells, such as deregulated, mutated, or overexpressed proteins [6]. Protein kinases constitute important molecular targets for the development of novel anticancer agents, and a number of kinase inhibitors have been developed. Some are already in clinical use [7,8,9].

Isatin (1*H*-indole-2,3-dione) is a versatile heterocyclic compound which has been attracting the attention of medicinal chemists worldwide as a synthetic building block due to the various biological activities of its derivatives. Among the latter, compounds bearing an oxindole scaffold constitute a group of promising compounds endowed with significant biological activities, such as anticonvulsant, antimicrobial, antitubercular [10], antileishmanial [11], anti-HIV [12], antioxidative [13], antiproliferative [14], and anticancer [15,16] activities.

Oxindole derivatives have been widely recognized in cancer therapeutics as multi-kinase inhibitors. The progress towards synthetic oxindole derivatives accelerated with the FDA approval of sunitinib for the treatment of metastatic renal cell cancer [17], which acts as an inhibitor of several tyrosine kinases at nanomolar concentrations [18]. Optimization of the substituents around the oxindole nucleus led to the development of several other oxindole-based kinase inhibitors, including toceranib, the only dog-specific anti-cancer drug [19], and nintedanib, which has been approved for use in the United States to treat idiopathic pulmonary fibrosis with a progressive phenotype [20].

Kinase inhibitors semaxanib and orantinib bear an oxindole moiety with a substituent at the C3 position [21]. Semaxanib has reached phase III clinical trials in the treatment of advanced colorectal cancer [22]; and orantinib is in phase II clinical trials for the treatment of breast cancer and in phase III clinical trials for the treatment of hepatocellular carcinoma [23].

Wang et al. have reported syntheses of isatin derivatives bearing an α,β-unsaturated ketone moiety as the only substituent of an oxindole moiety at C3, and they have promising anticancer properties which are dependent on the electron-donating substituents on the benzyl ring [24].

Hydrazone derivatives, whose biological activity is associated with the presence of the active azomethine pharmacophore, constitute another significant class of biologically active compounds in medicinal and pharmaceutical chemistry [25]. These compounds, in combination with various heterocyclic scaffolds, produce diverse biological results, including antioxidant and anticancer activities [26,27,28].

3-((2,6-Dichlorobenzylidene)hydrazono)indolin-2-one and 3-((2-chloro-6-fluorobenzylidene)hydrazono)indolin-2-one showed excellent activity against human breast adenocarcinoma cell line MCF7 using the MTT assay, whereas 3-((2-bromobenzylidene)hydrazono)indolin-2-one showed cytotoxicity against MCF7 and human ovarian adenocarcinoma (A2780) [29].

A hydrazone moiety has been used as a linker in the construction of bis-isatin compounds with vast structural variety and numerous biological activities [15]. Ibrahim et al. have reported syntheses of variously substituted bis(2-oxoindolin-3-ylidene)-1*H*-pyrrole-2,4-dicarbohydrazide derivatives, which have shown promising anticancer activity against HepG2 (liver), MCF-7 (breast), and HCT-116 (colon) human cancer cell lines [30].

5-Oxopyrrolidine is yet another scaffold incorporated in natural and synthetic biologically active compounds. The 2-pyrrolidinone rich fraction of *Brassica oleracea var. capitatahas* has been shown to exhibit antioxidant and in vitro anticancer activities [31]. Recently, the antifungal and anticancer properties exhibited by several compounds embedded with pyrrolidine-hydrazone and pyrrolidine-oxindole moieties have been reported [32,33].

As a continuation of our interest in further searching for the nitrogen-containing heterocyclic compounds possessing anticancer and antioxidant activities [34,35,36], we report herein the syntheses of a series of derivatives bearing one or two 2-oxindole-hydrazone moieties, and evaluations of their anticancer and antioxidant activities. Two human cell lines of different origins, namely, malignant melanoma (A375) and human colon adenocarcinoma (HT-29), were selected to test the proliferation inhibition and colony-forming inhibition of the synthesized compounds. Both cell lines are considered to be derived from very aggressive types of tumors, and resistance develops quite often during treatment with drugs [37,38]. In order to combat this resistance, combinations of drugs with different mechanisms of action are used. Kinase inhibitors such as dabrafenib [39] and dasatinib are often used [40]. Many more compounds are being studied at different stages of preclinical research. We decided to also explore the activity of novel compounds in tumor spheroids (3D cultures)—as they mimic the real tumor microenvironment much better than cell monolayers [41]—and identify the most promising hydrazone-isatin derivatives for further development.

## 2. Results and Discussion

### 2.1. Chemistry 

**Scheme 1:**
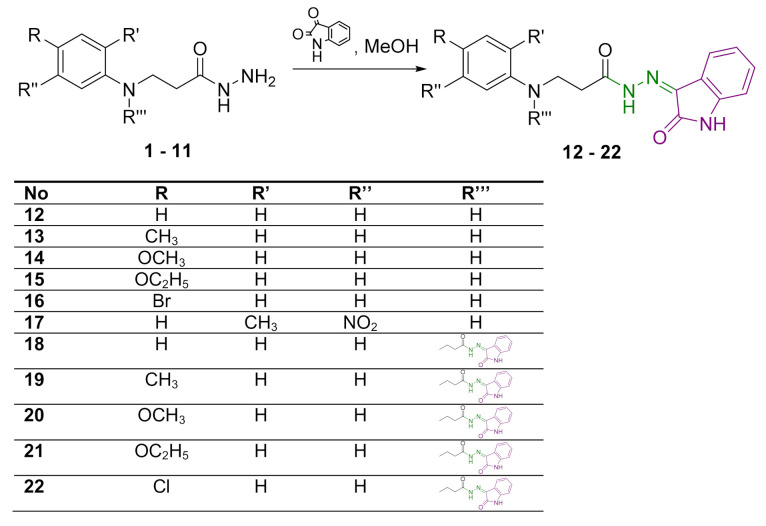
Syntheses of hydrazones **12**–**22**. Azomethine group is marked in green colour and 2-oxindole moiety is represented in purple colour.

Target hydrazones **12**–**17** were synthesized in reactions of the corresponding hydrazides **1**–**6**, each time with a slight excess (molar ratio 1:1.2) of isatin in methanol, in the presence of glacial acetic acid, at 65 °C, giving 58–96% yields (Scheme 1). 

In the ^1^H NMR spectra of hydrazones **12**–**17**, the proton of the secondary amine group adjacent to the phenyl ring (CH_2_*NH*) resonated as a singlet in the region of 5.26–5.97 ppm (Figures S1, S4, S7, S10, S13 and S16 in Supplementary Material). As expected, the amide-group proton gave a singlet at lower field at 11.12 ppm in the ^1^H NMR spectra of **12** and **16** (Figures S1 and S13 in Supplementary Material). Proton resonance at 10.78 ppm has been attributed to the secondary amine group in the 2-oxindole moiety, thereby—along with the increased intensity of the proton resonances in the aromatic region—confirming the presence of this moiety in the novel compounds. The ^1^H NMR spectra of hydrazones **13**–**15** and **17** display double sets of resonances of the CO–NH group protons, and a 2-oxindole NH proton with signal intensity ratio 0.7:0.3 due to the restricted rotation around the amide bond (Figures S4, S7, S10 and S16 in Supplementary Material). This splitting of the proton resonances indicates that in DMSO-*d_6_*, hydrazones exist as a mixture of *Z/E* isomers with respect to the hindered rotation around the amide bond. Usually, the *Z* isomer predominates [34,35,42]. In the ^1^H NMR spectra, the NH protons of *Z* isomers resonate at a lower field with respect to the resonances attributed to *E* isomers [43]. In the ^13^C NMR spectra for **12**–**17**, two carbonyl group carbon resonances in the range of 162–175 ppm confirm the presence of a 2-oxindole moiety along with the amide group (Figures S2, S5, S8, S11, S14 and S17 in Supplementary Material).

When dihydrazides **7**–**11** were treated with isatin in the molar ratio 1:2.4, hydrazones **18**–**22** bearing two 2-oxindole moieties were obtained. In this case, yields of the target compounds were 36–68%. The ^1^H NMR spectra for **18**, **19** and **22** display the double sets of amide-group-proton and 2-oxindole-amine-proton resonances, indicating mixtures of *Z/E* isomers in DMSO-*d_6_* solutions (Figure S19, S22 and S31 in Supplementary Material). 

**Scheme 2:**
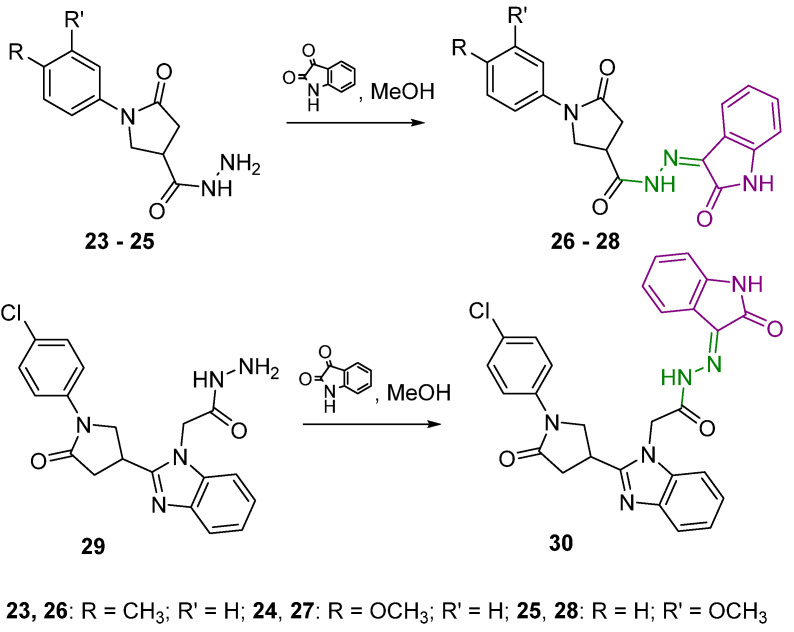
Syntheses of hydrazones **26**–**28** and **30**. Azomethine group is marked in green colour and 2-oxindole moiety is represented in purple colour.

With the aim of introducing a 2-oxindole fragment into a structure of a molecule containing the pyrrolidin-2-one moiety, reactions of 5-oxo-1-substituted phenylpyrrolidine-3-carbohydrazides **23**–**25** with isatin in the molar ratio 1:1.2 were carried out resulting in the formation of 5-oxo-*N′*-(2-oxoindolin-3-ylidene)pyrrolidine-3-carbohydrazides **26**–**28** bearing *m*-methoxy, *p*-methoxy, or *p*-chlorophenyl substituents (Scheme 2). 

2-(2-(1-(4-Chlorophenyl)-5-oxopyrrolidin-3-yl)-1*H*-benzo[d]imidazol-1-yl)-*N*′-(2-oxoindolin-3-ylidene)acetohydrazide (**30**) was obtained from a corresponding hydrazide **29** in the reaction with isatin (molar ratio 1:1.2). In the ^1^H NMR spectrum for **30**, two distinct proton sets at 10.78–10.88 ppm and 11.14–11.24 ppm attributed to the amine group proton in 2-oxindole fragment and amide proton are visible, indicating the formation of *Z/E* isomers in DMSO-*d_6_* solutions (Figure S43 in Supplementary Material).

**Scheme 3:**
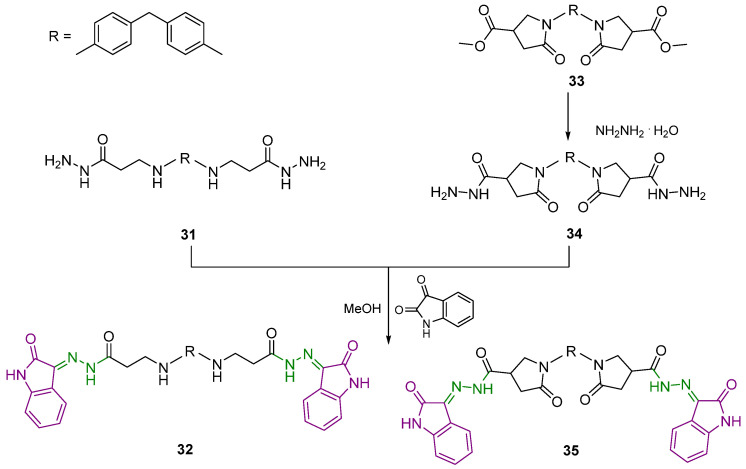
Synthesis of hydrazones **32** and **35**. Azomethine group is marked in green colour and 2-oxindole moiety is represented in purple colour.

Another set of isatin derivatives were developed by introducing a 2-oxindole fragment into structures of molecules bearing diphenylmethane and diphenylamine along with aliphatic and heterocyclic linkers between the benzene ring and hydrazone-oxindole moiety. First of all, a reaction of 3,3′-((methylenebis(4,1-phenylene))bis(azanediyl))di(propanehydrazide) (**31**) with isatin in the molar ratio 1:2.4 provided the target compound **32** bearing two *N*′-(2-oxoindolin-3-ylidene)propanehydrazide “arms” (Scheme 3). 

The next step required the introduction of the pyrrolidin-2-one moiety into the structure of the target compound **35**. Thus, 1-[4-({4-[4-(methoxycarbonyl)-2-oxopyrrolidine-1-il]phenyl}methyl)phenyl]-5-oxopyrrolidine-3-carboxylate (**33**) was synthesized by the classical esterification reaction of a corresponding diacid in methanol in the presence of H_2_SO_4_ as a catalyst. Afterwards, diester **33** was dissolved in DMSO and treated with hydrazine hydrate to afford dihydrazide **34**, which was subjected to a reaction with isatin in the molar ratio 1:2.4. Double intensities of the corresponding proton signals in pyrrolidinone and 2-oxindole moieties in the ^1^H NMR spectra for **33**–**35** prove the structures of the target molecules as having two 5-oxo-*N*′-(2-oxoindolin-3-ylidene)pyrrolidine-3-carbohydrazide “arms“ attached to the diphenylmethane core (Figures S49, S52 and S54 in Supplementary Material).

**Scheme 4:**
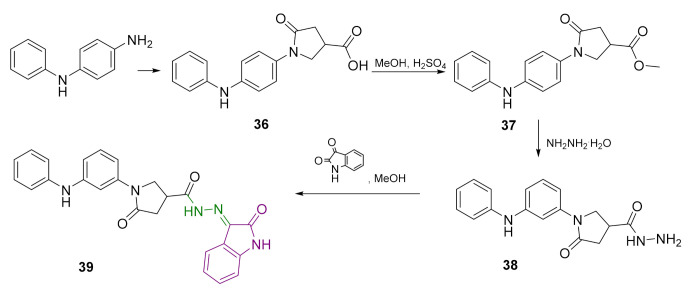
Synthesis of hydrazone **39**. Azomethine group is marked in green colour and 2-oxindole moiety is represented in purple colour.

Reaction of 4-aminodiphenylamine with itaconic acid at the reflux temperature of the reaction mixture provided 5-oxo-1-(4-(phenylamino)phenyl)pyrrolidine-3-carboxylic acid (**36**), which was first converted to ester **37** and then to hydrazide **38** according to the classical synthesis procedures (Scheme 4). Hydrazide **38** was treated with isatin in methanol at 65 °C to afford 5-oxo-*N′*-(2-oxoindolin-3-ylidene)-1-(4-(phenylamino)phenyl)pyrrolidine-3-carbohydrazide (**39**).

### 2.2. Pharmacology

#### 2.2.1. Anticancer Activity

**Figure 1:**
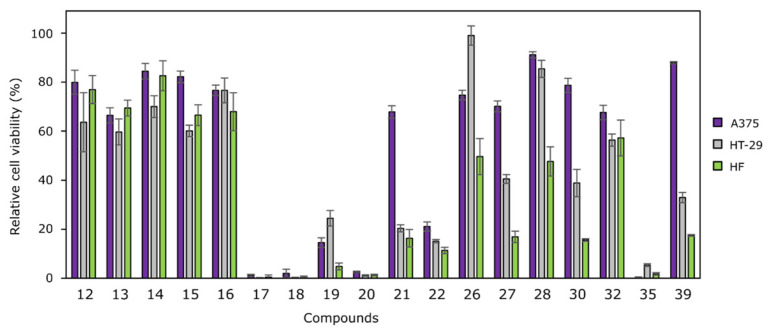
Effects of compounds **12**–**22**, **26**–**28**, **30**, **32**, **35** and **39** on cancer cell viability at 100 µM against human melanoma (A375) and human colon cancer (HT-29) cell lines, and human fibroblasts HF. Cell lines were incubated with 100 µM of compounds for 72 h. The cytotoxicity was measured using MTT assays. The cytotoxicity is represented as a percentage of viability, relative to the untreated cells (negative control, which is equal to 100%). N = 3.

The synthesized compounds **12**–**22**, **26**–**28**, **30**, **32**, **35** and **39** showed different levels of activity against human malignant melanoma (A375) and colon adenocarcinoma (HT-29) cell lines at 100 µM. This concentration was chosen based on our previous experience and the observations from other studies. It has been shown that 100 µM allows distinguishing the most active compounds from a group better than 10 µM [44]. A 100 µM concentration is suggested for primary screenings of anticancer agents [45]. It is also included in experiments as the highest concentration when establishing EC_50_ values [46,47]. In general, our compounds showed relatively low activity against the A375 cell line used in the screening experiments (Figure 1). Malignant melanoma is usually characterized as a cancer that is poorly responsive to many available chemotherapeutic agents due to different drug-resistance mechanisms [48]. HT-29 cells were more sensitive to the majority of the compounds tested. However, they did not have highly cytotoxic effects against this cell line. HT-29 cells are typically resistant to the majority of available drugs due to stemness [37], high expression of the MRP-1 P-glycoprotein that enhances drug efflux [49], and other mechanisms.

**Figure 2:**
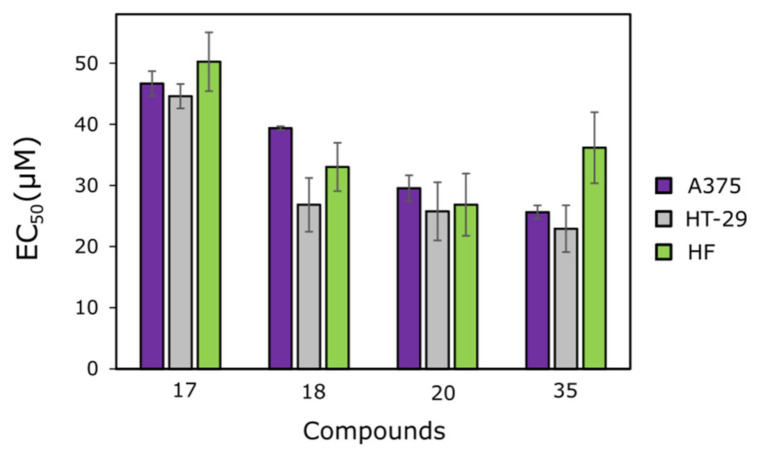
EC_50_ values of the most active compounds: **17**, **18**, **20** and **35**. Cell lines were incubated with different concentrations of those compounds for 72 h. The cytotoxicity was measured using MTT assays. The cytotoxicity is represented as the calculated half maximal effective concentration (EC_50_ value). N = 3.

Among mono-*N*-substituted *N*′-(2-oxoindolin-3-ylidene)propanehydrazides **12**–**17**, compound **17** bearing the 2-methyl-5-nitrobenzene moiety was identified as the most active one, whereas bis(hydrazone-isatins) **18** (bearing an unsubstituted benzene ring) and **20** (bearing the electron-donating methoxy group in benzene ring) were the most active among the compounds with two *N*′-(2-oxoindolin-3-ylidene)propanehydrazide “arms.” Another compound, which was selected for more thorough testing was diphenylmethane derivative **35** bearing two 5-oxo-*N*′-(2-oxoindolin-3-ylidene)pyrrolidine-3-carbohydrazide “arms” at *p*-positions of benzene rings. These four compounds reduced cell viability below 10%. The effective concentrations that reduced cell viability by 50% (EC_50_ values) were determined (Figure 2).

The mono-hydrazone-isatin **17**, bearing an electron-withdrawing nitro group at the *o*-position of the benzene ring, was the least active out of the four compounds selected, and it did not possess any selectivity towards cancer cell lines (EC_50_ = 46.7 ± 2.0 µM against A375, and EC_50_ = 44.6 ± 2.0 µM against HT-29) compared to fibroblasts (EC_50_ = 50.2 ± 4.8 µM). The cytotoxic effects of bis(hydrazone-isatins) **20** and **35** were similar against both cell lines; however, compound **35** showed less cytotoxicity toward fibroblasts, and this makes it a more promising one.

As a comparison, dacarbazine, which is a chemotherapeutic drug approved for melanoma treatment, is far less active and inhibits A375 cell survival only at high concentrations of 25–100 μM [50]. However, the clinically approved BRAF inhibitor dabrafenib reduces A375 cell viability by 50% at a nanomolar concentration after 72 h of incubation [51]. 5-Fluorouracil, which is approved as a chemotherapeutic agent against colon cancer, possesses a rather low cytotoxic effect after 72 h of incubation, and usually five or more days are needed for it to reach its EC_50_ value, which is >100 μM [52]. The kinase inhibitor regorafenib achieves 50% HT-29 cell viability reduction at 0.5 μM concentration after 6 h of incubation [53].

A clonogenic assay was used to evaluate each compound’s impact on the ability of a single cell to form a colony. Numbers of colonies (ability of single cells to survive) and the areas of colonies (ability to form a colony and proliferate) were determined. The most active compounds, **17**, **18**, **20** and **35**, were tested for their activity against human melanoma (A375) and colon adenocarcinoma (HT-29) cell lines at 50% of their EC_50_ values.

**Figure 3:**
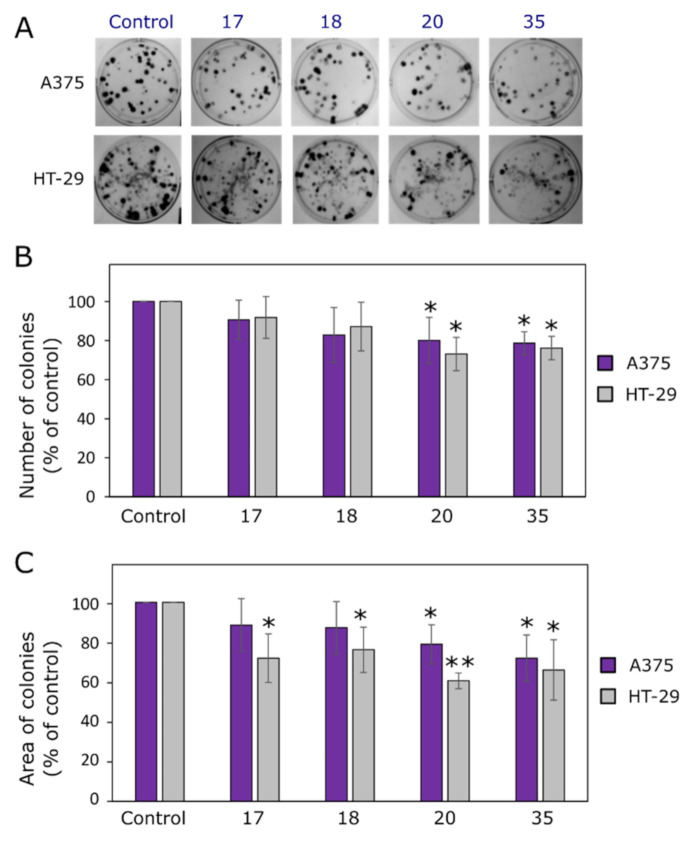
Effects of compounds **17**, **18**, **20** and **35** on colony formation. (**A**) Photos of A375 and HT-29 colonies at the end of the experiment. (**B**) Numbers of colonies at the end of the experiment. (**C**) Areas of colonies at the end of the experiment. Single asterisks (*) indicate *p* < 0.05 and pairs (**) indicate *p* < 0.01 compared to the control (untreated cell colonies).

The synthesized compounds showed higher activity against HT-29 colony formation (Figure 3). Derivatives **17** and **18** did not affect A375 colony number or colony area, compared to the control (p > 0.05), although both compounds slowed down the colony proliferation. Compound **20** was identified as the most active compound; its inhibitory effect on the ability to proliferate and form colonies was the highest. For instance, in A375 and HT-29 cell lines, the colony areas dropped down to 78.9% and 60.6%, respectively, and reached their lowest values in comparison to the control cells (Figure 3). The activity of diphenylmethane derivative **35** was similar to that of bis(hydrazone-isatin) **20** bearing the electron-donating methoxy group at *p*-position of benzene ring.

Malignant melanoma is one of the most aggressive tumor types, and A375 is characterized by high proliferation and migration rates, and high invasiveness potential [38]. This suggests the need to identify novel molecular targets and new therapeutic strategies. The combination strategy is one of the possible solutions that can overcome the resistance of melanoma cells to conventional therapy. Li and Han [54] have shown that a combination of dacarbazine and all-trans retinoic acid, loaded in lipid nanoformulations, is able to reduce B16F10’s colony formation ability, whereas dacarbazine alone shows a limited ability to inhibit colony formation. In several studies, the colony-forming effect of kinase inhibitors was assessed by clonogenic assay. Sinik et al. [55] determined that MERTK inhibitor UNC2025 decreased colony formation and cell density in most tested BRAF mutant and BRAF wild-type cell lines at 300–500 nM. Ross et al. [39] have determined that the antifolate methotrexate sensitizes resistant malignant melanoma cells to the kinase inhibitor dabrafenib, and their combination reduces colony formation by up to 42.5% relative to dabrafenib alone. Similarly, colon cancer is considered to be one of the most aggressive cancers worldwide, and it gains resistance to drugs quite often [37]. In order to combat the resistance, the approach of pretreating cancer cells with other compounds before adding chemotherapeutic agents is widely studied. By using clonogenic assays, it has been shown that the kinase inhibitor dasatinib could have a favorable synergistic effect with oxaliplatin, which is an approved drug for colon adenocarcinoma treatment [40]. A combination of 100 nM dasatinib and 2.5 µM oxaliplatin significantly reduced HT-29 (but not KM12-L4) colony growth after 14 days of incubation. In summary, the effects of different compounds on colony-forming ability depends a lot on the cell line used, and kinase inhibitors could sensitize cells to cytotoxic drugs. In our case, the hydrazone-isatin derivatives bear kinase inhibitor fragments and are supposed to have a similar mechanism of action. However, deeper analysis of mechanisms of action is needed.

**Figure 4:**
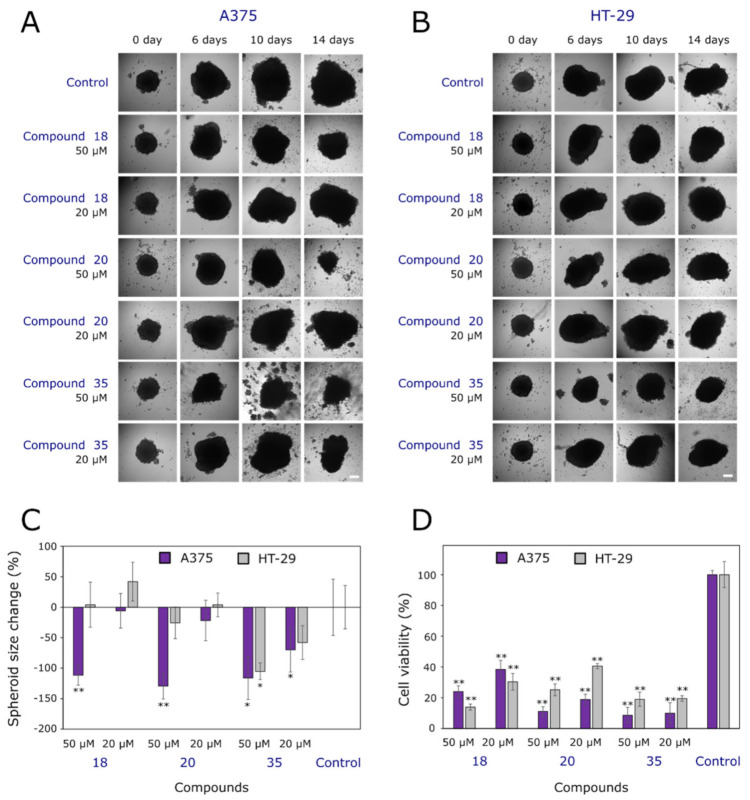
Effects of compounds **18**, **20** and **35** on 3D cell cultures. (**A**) Photos of human melanoma (A375) tumor spheroids at different periods of the experiment. (**B**) Photos of human colon adenocarcinoma (HT-29) tumor spheroids at different periods of the experiment. (**C**) Spheroid size at the end of the experiment. (**D**) Cell viability in A375 and HT-29 spheroids. Single asterisks (*) indicate *p* < 0.05, and pairs (**) indicate *p* < 0.01 compared to the control (untreated spheroids). Scale bars indicate 100 µm.

Nowadays, 3D cell culture models are becoming popular among cancer biologists due to the spatial arrangement of cells, and they enable the formation of hypoxia inside and a gradient of tested substances [41,56]. Such a model resembles a real tumor microenvironment and is more reminiscent of a real tumor than conventional cell monolayers. Thus, the effects of 20 and 50 µM solutions of bis(hydrazone-isatins) **18**, **20**, and **35** on melanoma A375 and colon adenocarcinoma HT-29 cell spheroid growth (Figure 4) were studied. As shown in Figure 4C, all tested compounds reduced A375 spheroid growth by ~120% at the higher concentration of 50 µM in comparison to the control group. Diphenylmethane derivative **35** showed an inhibitory effect on A375 spheroid growth at a concentration of 20 µM, too. Meanwhile, the effect on HT-29 3D culture growth was lower. Only compound **35** statistically significantly inhibited colon cancer cell spheroid growth (by ~105%) in comparison with the control (Figure 4C).

While taking into account that spheroid size does not always necessarily correlate with viability [57], we decided to study cell viability in all groups at the end of the experiment (Figure 4D). Interestingly, cell viability was from 2.5-fold to 10-fold lower compared to the control for both types of spheroids. This could be explained by cells at the cores of the spheroids being more affected by the compound, thereby becoming more hypoxic or even necrotic, denser, and less viable. Some spheroids at the end of the experiment became looser and irregular in shape, and started to disintegrate (Figure 4A,B). However, Golas et al. [58] determined that SKI-606, an inhibitor of Src and Abl kinases at the concentration of 2.5 µM, reduced the HT-29 spheroid size after the 6 days of incubation and concluded that SKI-606 strengthens cell–cell interactions. Cell viability was not measured at the end of this experiment, but the hypothesis regarding cell–cell interaction protection against SKI-606 treatment has been formed. Another study by Folkesson et al. [59] has revealed that MEK and TAK1 inhibitors strongly reduce cell viability both in 2D and 3D cell cultures, and combined treatments with a MEK inhibitor and conventional drugs produce synergistic effects in 3D models. Meanwhile, MEK inhibitors combined with STAT3 small-molecule inhibitors can cause cell death in 3D cultures [60]. This means that kinase inhibitors could be beneficial in reducing cell viability and inhibiting the growth of cells in 2D models.

In summary, compounds **20** and **35** have been identified as the most promising anticancer agents out of the series of hydrazone-isatin derivatives. Though their cytotoxicity against both cancer cell lines was not very high (EC_50_ values were in the range of 22–30 µM), they were more active than dacarbazine 5-fluorouracil and reforafenib, which are used to treat malignant melanoma and colon cancer. It is worth mentioning that the selectivity of the most promising compounds towards cancer cells (in comparison to fibroblasts) was not high. Compound **35** alone was 1.6 times more active against A375 and 1.4 times more selective against HT-29 compared to fibroblasts. However, the selectivity studies were performed in cell monolayers, and this is only the first step toward identifying the most active scaffolds for further development of more selective and more active anticancer agents. It should be worth exploring the accumulation of modified compounds in more sophisticated models, e.g., the three-dimensional models combining both fibroblasts and cancer cells, and thereby evaluating the fractions and types of surviving cells after compound treatment. Furthermore, the selectivity towards tumors is a major problem for many (even clinically-available) cytotoxic agents. Currently, many different approaches are used to improve selectivity, such as packing molecules into nanoformulations [61] and conjugating cytotoxic agents to antibodies [62].

#### 2.2.2. Antioxidant Activity

Reactive oxygen species (ROS) and reactive nitrogen species (NOS) are well known as both harmful and beneficial species [63]. However, imbalances between endogenous antioxidant defense and ROS oxidative stress have been related to an extensive range of diseases, including cardiovascular, inflammatory, neurodegenerative, and autoimmune ones. Overproduction of ROS can be responsible for damage to vital cell components, especially to DNA, lipids, and proteins. It is known that the use of antioxidants is beneficial in the prevention or delaying of numerous diseases associated with oxidative stress, including cancer, Alzheimer’s and other neurodegenerative diseases, and atherosclerosis [64,65,66,67]. Antioxidants are believed to prevent and treat various types of malignancies [68]. The compounds **12**–**22**, **26**–**28**, **30**, **32**, **35** and **39** were designed to contribute to radical-scavenging (to be antioxidants) [69,70].

Ferric ion (Fe^3+^), which is a relatively biologically inactive form of iron, can be reduced to the active Fe^2+^ depending on the conditions, particularly pH, and oxidized back through a Fenton-type reaction with the production of hydroxyl radicals or a Haber–Weiss reaction with the generation of superoxide anions [71,72]. Reducing power measures the reductive ability of an antioxidant, and it is evaluated by the transformation of Fe^3+^ to Fe^2+^ by donation of an electron in the presence of the tested compound [73].

**Figure 5:**
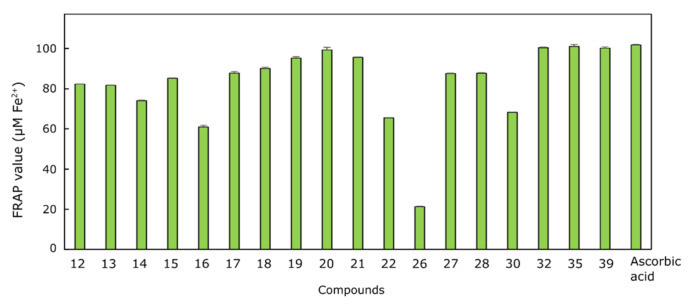
Antioxidant activity levels of compounds **12**–**22**, **26**–**28**, **30**, **32**, **35** and **39** tested by Ferric reducing antioxidant power assay. For each assay, 100 μL each of the tested compound solution (20 mM) and positive control (20 mM ascorbic acid) were mixed with 3 mL of the FRAP reagent (negative control: 3 mL FRAP reagent and 100 μL DMSO). The absorbance of the reaction mixture at 593 nm was measured spectrophotometrically after 5 min.

As seen from the results presented in Figure 5, compounds **32**, **35** and **39** possessed the highest capacity to reduce Fe(TPTZ)^3+^ to Fe(TPTZ)^2+^ in comparison with the positive control, ascorbic acid (103.41 µM). It is interesting to note that compounds **32** and **35** are bis(hydrazone-isatins), and compound **39** has just one 5-oxo-*N*′-(2-oxoindolin-3-ylidene)pyrrolidine-3-carbohydrazide “arm.” The relatively high ferric reducing antioxidant power of **39** proves the expectation that substitution of diphenylmethane with a diphenylamine moiety bearing a secondary amine group [74] enhances the reducing power of compound **39**. Another group of compounds which have been identified as possessing high antioxidant activity comprises bis(hydrazone-isatins) **19**–**21**, whose structures differ by a substituent at the *p*-position of benzene ring. Compound **20** bearing the electron-donating methoxy group in the benzene ring has shown higher radical scavenging ability than **19** and **21**. Compounds **19**–**21** have been found to be considerably more active than their analogues bearing just one *N*′-(2-oxoindolin-3-ylidene)propanehydrazide “arm“: **13**–**15**. In this group, the ethoxy substituent in **15** had a more significant positive effect on the activity of the molecule. In another group of compounds, **26**–**28**, bearing a pyrrolidin-2-one ring as a linker between the hydrazine-oxindole moiety and the *p*-substituted benzene ring, compound **26**, containing an electron-donating methyl group, was identified as the least active one. The position of the methoxy substituent in the benzene ring had no significant influence on the antioxidant activity of **27** and **28**.

## 3. Materials and Methods

### 3.1. Chemistry

#### 3.1.1. Chemical Reagents and Instruments

Reagents were purchased from Sigma-Aldrich (St. Lous, MO, USA) and TCI Europe N.V. (Zwijndrecht, Belgium). Reaction course and purity of the synthesized compounds were monitored by TLC using aluminum plates precoated with silica gel 60 F254 (MerckKGaA, Darmstadt, Germany). Melting points were determined on a MEL-TEMP (Electrothermal, A Bibby Scientific Company, Burlington, NJ, USA) melting point apparatus and are uncorrected. FT-IR spectra (ν, cm^−1^) were recorded on a Perkin–Elmer Spectrum BX FT–IR spectrometer using KBr pellets. The ^1^H and ^13^C-NMR spectra were recorded in DMSO-*d_6_* on a Bruker Avance III (400 MHz, 101 MHz) spectrometer operating in Fourier transform mode. Chemical shifts (*δ*) are reported in parts per million (ppm) calibrated from TMS (0 ppm) as an internal standard for ^1^H NMR, and DMSO-*d_6_* (39.43 ppm) for ^13^C NMR. Mass spectra were obtained on the Bruker maXis UHR-TOF mass spectrometer (Bruker Daltonics, Bremen, Germany) with ESI ionization.

#### 3.1.2. General Procedure for Synthesis of Compounds **12**–**17**

To a solution of a corresponding hydrazide (5 mmol) in methanol (25 mL), isatin (6 mmol) and glacial acetic acid (5 drops) were added. The reaction mixture was stirred at 65 °C for 15–20 min. Precipitate was filtered off, washed with methanol, and recrystallized from DMF/H_2_O mixture.

*N*′-(2-oxoindolin-3-ylidene)-3-(phenylamino)propanehydrazide (**12**)

Prepared from 3-(phenylamino)propanehydrazide (**1**) [75] (0.89 g). Yield 68% (1.05 g); yellow crystals; m.p. 207–208 °C; IR (KBr) ν_max_ (cm^−1^): 1695, 1728 (C=O), 3258, 3355, 3447 (NH); ^1^H NMR (400 MHz, DMSO-*d_6_*): δ 2.63–3.11 (m, 2H, CH_2_CO), 3.33–3.42 (m, 2H, *CH_2_*NH), 5.69 (s, 1H, CH_2_*NH*), 6.55 (t, 1H, *J* = 8.0 Hz, H_Ar_), 6.63 (d, 2H, *J* = 8.0 Hz, H_Ar_), 6.89 (d, 1H, *J* = 8.0 Hz, H_Ar_), 6.92–6.99 (m, 1H, H_Ar_), 7.09 (t, 2H, *J* = 8.0 Hz, H_Ar_), 7.36 (t, 1H, *J* = 8.0 Hz, H_Ar_), 7.76–8.30 (m, 1H, H_Ar_), 10.78 (s, 1H, NH_isatin_), 11.12 (s, 1H, NHCO); ^13^C NMR (101 MHz, DMSO-*d_6_*): δ 34.23 (*CH_2_*CO), 38.38 (CH_2_NH), 110.56, 112.24, 115.30, 115.90, 121.63, 126.02, 128.98, 132.43, 143.65, 148.49 (C_Ar_), 164.67, 170.35 (C=O); HRMS (ESI): *m*/*z* calcd for C_17_H_16_N_4_O_2_ 309.1352 [M+H]^+^, found 309.1346.

*N*′-(2-oxoindolin-3-ylidene)-3-(*p*-tolylamino)propanehydrazide (**13**)

Prepared from 3-(*p*-tolylamino)propanehydrazide (**2**) [76,77] (0.97 g). Yield 96% (1.54 g); yellow crystals; m.p. 215–216 °C; IR (KBr) ν_max_ (cm^−1^): 1694, 1728 (C=O), 3132, 3244, 3356 (NH); ^1^H NMR (400 MHz, DMSO-*d_6_*): δ 2.14 (s, 3H, CH_3_), 2.63–3.39 (m, 4H, CH_2_CO+*CH_2_*NH), 5.46 (s, 1H, CH_2_*NH*), 6.47–6.57 (m, 2H, H_Ar_), 6.86–6.95 (m, 3H, H_Ar_), 7.08 (t, 1H, *J* = 8.0 Hz, H_Ar_), 7.36 (t, 1H, *J* = 8.0 Hz, H_Ar_), 7.40–7.64 (m, 1H, H_Ar_), 10.77 (s, 0.3H, NHCO), 11.11 (s, 0.3H, NH_isatin_), 11.23 (s, 0.7H, NH_isatin_), 12.54, 12.97 (2s, 0.7H, NHCO);; ^13^C NMR (101 MHz, DMSO-*d_6_*): δ 20.08 (CH_3_), 31.16 (*CH_2_*CO), 40.19 (CH_2_NH), 111.09, 112.28, 115.28, 121.61, 122.50, 124.21, 125.99, 129.37, 131.39, 133.91, 142.29, 146.18 (C_Ar_), 162.46, 173.86 (C=O); HRMS (ESI): *m*/*z* calcd for C_18_H_18_N_4_O_2_ 323.1509 [M+H]^+^, found 323.1503.

3-((4-Methoxyphenyl)amino)-*N*′-(2-oxoindolin-3-ylidene)propanehydrazide (**14**)

Prepared as described in [35].

3-((4-Ethoxyphenyl)amino)-*N*′-(2-oxoindolin-3-ylidene)propanehydrazide (**15**)

Prepared from 3-((4-ethoxyphenyl)amino)propanehydrazide (**4**) [78] (1.12 g). Yield 58% (0.97 g); yellow crystals; m.p. 208–209 °C. IR (KBr) ν_max_ (cm^−1^): 1695, 1728 (C=O), 3156, 3267, 3341 (NH); ^1^H NMR (400 MHz, DMSO-*d_6_*): δ 1.26 (t, 3H, *J* = 6.9 Hz, *CH_3_*CH_2_O), 2.66–3.10 (m, 2H, CH_2_CO), 3.29–3.39 (m, 2H, *CH_2_*NH), 3.83–3.92 (m, 2H, CH_3_*CH_2_*O), 5.26 (s, 1H, CH_2_*NH*), 6.54–6.60 (m, 2H, H_Ar_), 6.72 (t, 2H, *J* = 8.0 Hz, H_Ar_), 6.88–7.09 (m, 2H, H_Ar_), 7.36 (t, 1H, *J* = 8.0 Hz, H_Ar_), 7.47–8.14 (m, 1H, H_Ar_), 10.78 (s, 0.7H, NHCO), 11.14 (s, 0.7H, NH_isatin_), 11.23 (s, 0.3H, NH_isatin_), 12.54, 12.97 (2s, 0.3H, NHCO); ^13^C NMR (101 MHz, DMSO-*d_6_*): δ 14.89 (*CH_3_*CH_2_O), 31.17 (*CH_2_*CO), 40.19 (CH_2_NH), 63.30 (CH_2_*CH_2_*O), 110.55, 113.26, 115.28, 115.45, 121.61, 125.97, 131.40, 132.42, 142.65, 143.63, 150.03 (C_Ar_), 162.47, 173.92 (C=O); HRMS (ESI): *m*/*z* calcd for C_19_H_20_N_4_O_3_ 353.1614 [M+H]^+^, found 353.1608.

3-((4-Bromophenyl)amino)-*N*′-(2-oxoindolin-3-ylidene)propanehydrazide (**16**)

Prepared from 3-((4-bromophenyl)amino)propanehydrazide (**5**) [79] (1.29 g). Yield 73% (1.41 g); yellow crystals; m.p. 218–219 °C. IR (KBr) ν_max_ (cm^−1^): 1695, 1726 (C=O), 3136, 3257, 3356 (NH); ^1^H NMR (400 MHz, DMSO-*d_6_*): δ 2.69–3.17 (m, 2H, CH_2_CO), 3.35 (s, 2H, *CH_2_*NH), 5.97 (s, 1H, CH_2_*NH*), 6.60 (d, 2H, *J* = 8.0 Hz, H_Ar_), 6.89 (d, 1H, *J* = 8.0 Hz, H_Ar_), 7.00 (t, 1H, *J* = 8.0 Hz, H_Ar_), 7.21 (d, 2H, *J* = 8.0 Hz, H_Ar_), 7.36 (t, 1H, *J* = 8.0 Hz, H_Ar_), 7.80–8.21 (m, 1H, H_Ar_), 10.78 (s, 1H, NH_isatin_), 11.12 (s, 1H, NHCO); ^13^C NMR (101 MHz, DMSO-*d_6_*): δ 32.27 (*CH_2_*CO), 38.41 (CH_2_NH), 106.36, 110.55, 114.10, 115.27, 121.62, 126.07, 131.45, 132.41, 143.65, 147.72 (C_Ar_), 164.63, 175.44 (C=O); HRMS (ESI): *m*/*z* calcd for C_17_H_15_BrN_4_O_2_ 387.0457 [M+H]^+^, found 387.0451.

3-((2-Methyl-5-nitrophenyl)amino)-*N*′-(2-oxoindolin-3-ylidene)propanehydrazide (**17**)

Prepared from 3-((2-methyl-5-nitrophenyl)amino)propanehydrazide (**6**) [76,80] (1.2 g). Yield 95% (1.93 g); yellow crystals; m.p. 205–206 °C; IR (KBr) ν_max_ (cm^−1^): 1699, 1729 (C=O), 3160, 3191, 3419 (NH); ^1^H NMR (400 MHz, DMSO-*d_6_*): δ 2.16 (s, 3H, CH_3_), 2.73–3.18 (m, 2H, CH_2_CO), 3.47–3.63 (m, 2H, *CH_2_*NH), 5.66 (s, 1H, CH_2_*NH*), 6.85–6.94 (m, 1H, H_Ar_), 7.00 (t, 1H, *J* = 8.0 Hz, H_Ar_), 7.21 (d, 2H, *J* = 8.0 Hz, H_Ar_), 7.28–7.42 (m, 3H, H_Ar_), 7.80–8.19 (m, 1H, H_Ar_), 10.77 (s, 0.7H, NHCO), 11.12 (s, 0.7H, NH_isatin_), 11.23 (s, 0.3H, NH_isatin_), 12.57, 12.96 (2s, 0.3H, NHCO); ^13^C NMR (101 MHz, DMSO-*d_6_*): δ 17.88 (CH_3_), 40.19 (*CH_2_*CO), 46.73 (CH_2_NH), 102.25, 110.56, 111.10, 115.28, 121.61, 126.05, 130.21, 130.37, 132.45, 142.30, 143.66, 147.01, 147.22 (C_Ar_), 162.47, 164.65 (C=O); HRMS (ESI): *m*/*z* calcd for C_18_H_17_N_5_O_4_ 368.1360 [M+H]^+^, found 368.1353.

#### 3.1.3. General Procedure for Synthesis of Compounds **18**–**22**

To a solution of corresponding hydrazide (5 mmol) in methanol (25 mL), isatin (12 mmol) and glacial acetic acid (5 drops) were added. The reaction mixture was stirred at 65 °C for 15 min. Precipitate was filtered off, washed with methanol, and recrystallized from DMF/H_2_O mixture.

3-((3-Oxo-3-(2-(2-oxoindolin-3-ylidene)hydrazinyl)propyl)(phenyl)amino)-*N*′-(2-oxoindolin-3-ylidene)propanehydrazide (**18**)

Prepared from 3,3′-(phenylazanediyl)di(propanehydrazide) (**7**) [81] (1.33 g). Yield 60% (1.57 g); yellow crystals; m.p. 191–192 °C; IR (KBr) ν_max_ (cm^−1^): 1694, 1730 (C=O), 3213, 3305 (NH); ^1^H NMR (400 MHz, DMSO-*d_6_*): δ 2.32 (t, 1.7H, *J* = 6.0 Hz, CH_2_CO), 3.33–3.76 (m, 6.3H, CH_2_CO+CH_2_N), 6.55–6.69 (m, 1.5H, H_Ar_), 6.71–6.83 (m, 1.5H, H_Ar_), 6.87–6.91 (m, 3H, H_Ar_), 6.94–7.06 (m, 2H, H_Ar_), 7.08–7.27 (m, 3H, H_Ar_), 7.28–7.41 (m, 2H, H_Ar_), 9.06 (s, 0.6H, NHCO), 10.76, 10.79 (2s, 1.4H, NH_isatin_), 11.09, 11.21 (2s, 1.4H, NHCO+NH_isatin_), 12.54, 12.93 (2s, 0.6H, NHCO);; ^13^C NMR (101 MHz, DMSO-*d_6_*): δ 31.61 (*CH_2_*CO), 46.95 (CH_2_N), 110.55, 111.93, 115.27, 115.79, 121.66, 126.09, 129.29, 129.39, 132.46, 143.66, 146.88 (C_Ar_), 164.65, 169.98 (C=O); HRMS (ESI): *m*/*z* calcd for C_28_H_25_N_7_O_4_ 524.2047 [M+H]^+^, found 524.2041.

3-((3-Oxo-3-(2-(2-oxoindolin-3-ylidene)hydrazinyl)propyl)(*p*-tolyl)amino)-*N*′-(2-oxoindolin-3-ylidene)propanehydrazide (**19**)

Prepared from 3,3′-(*p*-tolylazanediyl)di(propanehydrazide) (**8**) [81] (1.40 g). Yield 51% (1.36 g); orange crystals; m.p. 180–181 °C; IR (KBr) ν_max_ (cm^−1^): 1689, 1728 (C=O), 3176, 3212 (NH); ^1^H NMR (400 MHz, DMSO-*d_6_*): δ 2.17 (s, 3H, CH_3_), 2.30 (t, 1.5H, *J* = 7.0 Hz, CH_2_CO), 3.20–3.84 (m, 6.5H, CH_2_CO+CH_2_N), 6.60–6.72 (m, 1.5H, H_Ar_), 6.72–6.83 (m, 1.5H, H_Ar_), 6.84–6.92 (m, 2H, H_Ar_), 6.94–7.08 (m, 5H, H_Ar_), 7.72–8.28 (m, 2H, H_Ar_), 9.05 (s, 0.6H, NHCO), 10.76, 10.78 (s, 1.7H, NH_isatin_), 11.07 (s, 1.2H, NHCO), 11.21 (s, 0.3H, NH_isatin_), 12.52, 12.92 (2s, 0.2H, NHCO); ^13^C NMR (101 MHz, DMSO-*d_6_*): δ 19.92 (CH_3_), 31.66 (*CH_2_*CO), 47.18 (CH_2_N), 110.55, 112.38, 115.28, 121.62, 126.11, 129.75, 129.85, 132.42, 142.30, 143.66, 144.82 (C_Ar_), 162.45, 164.66 (C=O); HRMS (ESI): *m*/*z* calcd for C_29_H_27_N_7_O_4_ 538.2204 [M+H]^+^, found 538.2197.

3-((4-Methoxyphenyl)(3-oxo-3-(2-(2-oxoindolin-3-ylidene)hydrazinyl)propyl)amino)-*N*′-(2-oxoindolin-3-ylidene)propanehydrazide (**20**)

Prepared from 3,3′-((4-methoxyphenyl)azanediyl)di(propanehydrazide) (**9**) [82] (1.48 g). Yield 68% (1.88 g); orange crystals; m.p. 172–173 °C; IR (KBr) ν_max_ (cm^−1^): 1692, 1723 (C=O), 3225, 3429 (NH); ^1^H NMR (400 MHz, DMSO-*d_6_*): δ 3.17–3.66 (m, 11H, CH_2_CO+CH_2_N+CH_3_O), 6.60–7.01 (m, 7H, H_Ar_), 7.00 (t, 2H, *J* = 8.0 Hz, H_Ar_), 7.35 (t, 2H, *J* = 8.0 Hz, H_Ar_), 7.96–8.18 (m, 1H, H_Ar_), 9.04, 9,78 (2s, 0.6H, NHCO), 10.75, 10.77, 10.79 (3s, 1.7H, NH_isatin_), 11.06, (s, 1.2H, NHCO), 11.20 (s, 0.3H, NH_isatin_), 12.51, 12.93 (2s, 0.2H, NHCO); ^13^C NMR (101 MHz, DMSO-*d_6_*): δ 39.98, 40.19 (*CH_2_*CO) 46.97, 47.75 (CH_2_N), 55.27 (CH_3_O), 110.53, 110.05, 111.09, 114.85, 114.94, 115.27, 121.61, 126.09, 132.41, 141.41, 142.28, 143.65, 151.17 (C_Ar_), 162.43, 164.70, 170.11 (C=O); HRMS (ESI): *m*/*z* calcd for C_29_H_27_N_7_O_5_ 554.2153 [M+H]^+^, found 554.2146.

3-((4-Ethoxyphenyl)(3-oxo-3-(2-(2-oxoindolin-3-ylidene)hydrazinyl)propyl)amino)-*N*′-(2-oxoindolin-3-ylidene)propanehydrazide (**21**)

Prepared from 3,3′-((4-ethoxyphenyl)azanediyl)di(propanehydrazide) (**10**) [82] (1.7 g). Yield 44% (270 mg); orange crystals; m.p. 178–180 °C; IR (KBr) ν_max_ (cm^−1^): 1693, 1726 (C=O), 3240, 3452 (NH); ^1^H NMR (400 MHz, DMSO-*d_6_*): δ 1.28 (t, 3H, *J* = 8.0 Hz, *CH_3_*CH_2_O), 2.66–3.09 (m, 4H, CH_2_CO), 3.46–3.65 (m, 4H, CH_2_N), 3.90 (q, 2H, *J* = 8.0 Hz, CH_3_*CH_2_*O), 6.63–6.77 (m, 1H, H_Ar_), 6.78–6.83 (m, 4H, H_Ar_), 6.85–6.92 (m, 2H, H_Ar_), 6.95–7.08 (m, 2H, H_Ar_), 7.26–7.42 (m, 2H, H_Ar_), 8.04 (s, 1H, H_Ar_), 9.03 (s, 0.3H, NHCO), 10.77, 10.79 (3s, 1.8H, NH_isatin_), 11.08 (s, 1.7H, NHCO+NH_isatin_), 12.51, 12.93 (2s, 0.2H, NHCO); ^13^C NMR (101 MHz, DMSO-*d_6_*): δ 14.89 (*CH_3_*CH_2_O), 31.66 (*CH_2_*CO), 47.72 (CH_2_N), 63.28 (CH_3_*CH_2_*O), 110.54, 114.51, 115.30, 115.59, 121.63, 126.13, 132.45, 141.36, 141.50, 143.66, 150.40 (C_Ar_), 164.68, 170.15 (C=O); HRMS (ESI): *m*/*z* calcd for C_30_H_29_N_7_O_5_ 568.2309 [M+H]^+^, found 568.2308.

3-((4-Chlorophenyl)(3-oxo-3-(2-(2-oxoindolin-3-ylidene)hydrazinyl)propyl)amino)-*N*′-(2-oxoindolin-3-ylidene)propanehydrazide (**22**)

Prepared from 3,3′-((4-chlorophenyl)azanediyl)di(propanehydrazide) (**11**) [83] (1.58 g). Yield 36% (1.09 g) yield, yellow crystals; m.p. 193–195 °C; IR (KBr) ν_max_ (cm^−1^): 1689, 1726 (C=O), 3224, 3411 (NH); ^1^H NMR (400 MHz, DMSO-*d_6_*): δ 2.30 (t, 2H, *J* = 8.0 Hz, CH_2_CO), 3.34–3.70 (m, 6H, CH_2_CO+CH_2_N), 6.66–6.81 (m, 2H, H_Ar_), 6.86–6.93 (m, 3H, H_Ar_), 6.95–7.09 (m, 2H, H_Ar_), 7.14–7.25 (m, 2H, H_Ar_), 7.27–7.43 (m, 2H, H_Ar_), 8.09 (s, 1H, H_Ar_), 9.05 (s, 0.6H, NHCO), 10.77, 10.80 (2s, 1.7H, NH_isatin_), 11.11 (s, 1.2H, NHCO), 11.21 (s, 0.3H, NH_isatin_), 12.53, 12.87 (2s, 0.2H, NHCO); ^13^C NMR (101 MHz, DMSO-*d_6_*): δ 31.51 (*CH_2_*CO), 46.28, 47.02 (CH_2_N), 110.56, 113.47, 113.67, 115.26, 121.65, 126.12, 128.89, 128.94, 129.00, 132.49, 143.68, 145.77 (C_Ar_), 166.62, 169.84 (C=O); HRMS (ESI): *m*/*z* calcd for C_28_H_24_ClN_7_O_4_ 558.1657 [M+H]^+^, found 559.1706.

#### 3.1.4. 5-Oxo-*N*′-(2-oxoindolin-3-ylidene)-1-(*p*-tolyl)pyrrolidine-3-carbohydrazide (**26**)

Prepared from 5-oxo-1-(*p*-tolyl)pyrrolidine-3-carbohydrazide (**23**) [84] (1.17g) according to the synthesis procedure for compounds **12**–**17**. Yield 89% (1.62 g); yellow crystals; m.p. 268–269 °C; IR (KBr) ν_max_ (cm^−1^): 1639, 1682, 1690 (C=O), 3160, 3282 (NH); ^1^H NMR (400 MHz, DMSO-*d_6_*): δ 2.27 (s, 3H, CH_3_), 2.77–2.93 (m, 2H, CH_2_CO), 3.90–4.27 (m, 3H, N*CH_2_*CH+CH), 6.90 (d, 0.7H, *J* = 8.0 Hz, H_Ar_), 6.94 (d, 0.3H, *J* = 8.0 Hz, H_Ar_), 6.99–7.11 (m, 1H, H_Ar_), 7.17 (d, 2H, *J* = 8.0 Hz, H_Ar_), 7.34–7.42 (m, 1H, H_Ar_), 7.54 (d, 2H, *J* = 8.0 Hz, H_Ar_), 10.82 (s, 0.7H, NHCO), 11.27, 11.34 (2s, 1H, NH_isatin_), 12.59, 13.08 (2s, 0.3H, NHCO); ^13^C NMR (101 MHz, DMSO-*d_6_*): δ 20.43 (CH_3_), 32.31 (NCO*CH_2_*), 34.66 (CH), 49.78 (NCH_2_), 110.62, 111.14, 115.20, 119.54, 121.67, 126.22, 129.09, 133.20, 136.70, 142.49, 143.86 (C_Ar_), 162.47, 164.57, 171.50 (C=O); HRMS (ESI): *m*/*z* calcd for C_20_H_18_N_4_O_3_ 362.1379 [M+H]^+^, found 363.1453.

#### 3.1.5. 1-(4-Methoxyphenyl)-5-oxo-*N*′-(2-oxoindolin-3-ylidene)pyrrolidine-3-carbohydrazide (**27**)

Prepared from 1-(4-methoxyphenyl)-5-oxopyrrolidine-3-carbohydrazide (**24**) [85] (1.25 g) according to the synthesis procedure for compounds **12**–**17**. Yield 82% (1.55 g); yellow crystals; m.p. 203–204 °C; IR (KBr) ν_max_ (cm^−1^): 1595, 1668, 1689 (C=O), 3198, 3213 (NH); ^1^H NMR (400 MHz, DMSO-*d_6_*): δ 2.71–2.95 (m, 2H, CH_2_CO), 3.73 (s, 3H, CH_3_O), 3.91–4.26 (m, 3H, NCH_2_+CH), 6.86 (d, 2H, *J* = 8.0 Hz, H_Ar_), 6.89–7.01 (m, 2H, H_Ar_), 7.08 (t, 0.6H, *J* = 8.0 Hz, H_Ar_), 7.14 (t, 1.4H, *J* = 8.0 Hz, H_Ar_), 7.36 (d, 1H, *J* = 8.0 Hz, H_Ar_), 7.55 (d, 1H, *J* = 8.0 Hz, H_Ar_), 10.82, 11.26, 12.59, 13.09 (4s, 2H, NHCO+NH_isatin_); ^13^C NMR (101 MHz, DMSO-*d_6_*): δ 32.35 (CO*CH_2_*), 34.48 (CH), 50.07 (N*CH_2_*), 55.22 (CH_3_O), 109.97, 113.84, 115.21, 117.46, 121.36, 126.23, 127.03, 132.31, 138.65, 142.50, 155.95 (C_Ar_), 162.48, 162.78, 171.22 (C=O); HRMS (ESI): *m*/*z* calcd for C_20_H_18_N_4_O_4_ 378.1328 [M+H]^+^, found 379.1400.

#### 3.1.6. 1-(3-Methoxyphenyl)-5-oxo-*N*′-(2-oxoindolin-3-ylidene)pyrrolidine-3-carbohydrazide (**28**)

Prepared from 1-(3-methoxyphenyl)-5-oxopyrrolidine-3-carbohydrazide (**25**) [86,87] (1.25 g) according to the synthesis procedure for compounds **12**–**17,** just DMSO (10 mL) was used instead of methanol. Yield 85% (1.61 g); yellow crystals; m.p. 189–190 °C; IR (KBr) ν_max_ (cm^−1^): 1615, 1685, 1700 (C=O), 3191, 3265 (NH); ^1^H NMR (400 MHz, DMSO-*d_6_*): δ 2.73–2.95 (m, 2H, CH_2_CO), 3.75 (s, 3H, CH_3_O), 3.93–4.25 (m, 3H, NCH_2_+CH), 6.73 (d, 1H, *J* = 8.0 Hz, H_Ar_), 6.90 (d, 0.5H, *J* = 8.0 Hz, H_Ar_), 6.95 (d, 0.5H, *J* = 8.0 Hz, H_Ar_), 7.09 (t, 1H, *J* = 8.0 Hz, H_Ar_), 7.18 (d, 1H, *J* = 8.0 Hz, H_Ar_), 7.28 (t, 1H, *J* = 8.0 Hz, H_Ar_), 7.31–7.43 (m, 2H, H_Ar_), 7.51–7.66 (m, 0.5H, H_Ar_), 8.13 (s, 0.5H, H_Ar_), 10.82 (s, 0.5H, NHCO), 11.27 (s, 0.5H, NH_isatin_), 11.37 (s, 0.5H, NH_isatin_), 12.59, 13.07 (2s, 0.5H, NHCO); ^13^C NMR (101 MHz, DMSO-*d_6_*): δ 32.24 (CO*CH_2_*), 34.87 (CH), 49.86 (NCH_2_), 55.12 (CH_3_O), 105.61, 109.56, 110.63, 111.15, 111.69, 115.20, 119.77, 121.68, 122.58, 129.51, 131.67, 140.28, 143.86 (C_Ar_), 159.45, 162.48, 171.87 (C=O); HRMS (ESI): *m*/*z* calcd for C_20_H_18_N_4_O_4_ 378.1328 [M+H]^+^, found 379.1400.

#### 3.1.7. 2-(2-(1-(4-Chlorophenyl)-5-oxopyrrolidin-3-yl)-1*H*-benzo[d]imidazol-1-yl)-*N*′-(2-oxoindolin-3-ylidene)acetohydrazide (**30**)

Prepared from 2-(2-(1-(4-chlorophenyl)-5-oxopyrrolidin-3-yl)-1*H*-benzo[d]imidazol-1-yl)acetohydrazide (**29**) [34] (1.92 g) according to the synthesis procedure for compounds **12**–**17**. Yield 55% (1.40 g); orange crystals; m.p. 216–217 °C; IR (KBr) ν_max_ (cm^−1^): 1616, 1695, 1727 (C=O), 3269, 3340 (NH); ^1^H NMR (400 MHz, DMSO-*d_6_*): δ 2.70–3.09 (m, 2H, CH_2_CO), 3.33 (s, 2H, NCOCH_2_), 3.86–3.91 (m, 2H, N*CH_2_*CH), 5.29 (s, 1H, CH), 6.59 (d, 2H, *J* = 8.0 Hz, H_Ar_), 6.73 (d, 2H, *J* = 8.0 Hz, H_Ar_), 6.89 (d, 1H, *J* = 8.0 Hz, H_Ar_), 6.92–7.27 (m, 2H, H_Ar_), 7.36 (t, 2H, *J* = 8.0 Hz, H_Ar_), 7.43–7.56 (m, 1H, H_Ar_),7.66 (d, 0.5H, *J* = 8.0 Hz, H_Ar_), 7.72 (d, 0.5H, *J* = 8.0 Hz, H_Ar_), 7.81–8.32 (m, 1H, H_Ar_), 10.78, 10.88 (2s, 1H, NH_isatin_), 11.14, 11.24 (2s, 1H, NHCO); ^13^C NMR (101 MHz, DMSO-*d_6_*): δ 28.47 (CH), 39.98 (*CH_2_*CO), 43.16 (CO*CH_2_*), 52.09 (N*CH_2_*CH), 110.55, 113.41, 115.28, 115.45, 119.87, 120.93, 121.62, 125.98, 128.59, 130.48, 132.4, 132.45, 136.22, 137.33, 140.73, 142.61, 143.63, 155.12 (C_Ar_), 160.24, 164.63, 170.08 (C=O); HRMS (ESI): *m*/*z* calcd for C_27_H_21_ClN_6_O_3_ 536.1343 [M+H+Na]^+^, found 536.1645.

#### 3.1.8. (*N*′,*N*‴)-3,3′-((methylenebis(4,1-phenylene))bis(azanediyl))bis(*N*′-(2-oxoindolin-3-ylidene)propanehydrazide) (**32**)

Prepared from 3,3′-((methylenebis(4,1-phenylene))bis(azanediyl))di(propanehydrazide) (**31**) [88] (0.85 g) according to the synthesis procedure for compounds **18**–**22**. Yield 42% (380 mg); orange crystals; m.p. 166–167 °C; IR (KBr) ν_max_ (cm^−1^): 1692, 1730 (C=O), 3160, 3225, 3352 (NH); ^1^H NMR (400 MHz, DMSO-*d_6_*): δ 2.59–3.43 (m, 8H, CH_2_CO+*CH_2_*NH), 3.62 (s, 2H, CH_2_), 5.53 (s, 2H, CH_2_*NH*), 6.39–6.68 (m, 4H, H_Ar_), 6.73–7.13 (m, 8H, H_Ar_), 7.35 (t, 2H, *J* = 8.0 Hz, H_Ar_), 7.43–8.24 (m, 2H, H_Ar_), 9.85, 10.77, 11.03, 11.11, 11.23, 12.54, 12.97 (7s, 4H, NHCO+NH_isatin_); ^13^C NMR (101 MHz, DMSO-*d_6_*): δ 23.22 (*CH_2_*CO), 39.98 (CH_2_), 50.97 (CH_2_NH), 110.54, 112.37, 115.28, 121.62, 126.01, 129.07, 129.13, 132.42, 142.30, 143.63, 146.45 (C_Ar_), 162.48, 168.56 (C=O); HRMS (ESI): *m*/*z* calcd for C_35_H_32_N_8_O_4_ 629.2626 [M+H]^+^, found 629.2619.

#### 3.1.9. Methyl 1-[4-({4-[4-(methoxycarbonyl)-2-oxopyrrolidine-1-il]phenyl}methyl)phenyl]-5-oxopyrrolidine-3-carboxylate (**33**)

A mixture of 1,1′-(methylenebis(4,1-phenylene))bis(5-oxopyrrolidine-3-carboxylic acid) 18 g, 43 mmol), methanol (200 mL), and sulfuric acid (10 mL) was heated at reflux for 1 h (until formation of precipitate). Precipitate was filtered off and recrystallized from DMF/H_2_O mixture. Yield 47% (8.95 g); white crystals; m. p. 166–167 °C; IR (KBr) ν_max_ (cm^−1^): 1688, 1727 (C=O); ^1^H NMR (400 MHz, DMSO-*d_6_*): δ 2.67–2.85 (m, 4H, CH_2_CO), 3.41–3.48 (m, 2H, CH), 3.67 (s, 6H, CH_3_), 3.89 (s, 2H, CH_2_), 3.92–4.06 (m, 4H, CH_2_N), 7.21 (d, 4H, *J* = 8.8 Hz, H_Ar_), 7.54 (d, 4H, *J* = 8.8 Hz, H_Ar_); ^13^C NMR (101 MHz, DMSO-*d_6_*): 34.95 (*CH_2_*CO), 34.98 (CH), 39.84 (CH_2_), 49.77 (NCH_2_), 52.19 (CH_3_), 119.67, 128.88, 137.10, 137.25 (C_Ar_), 171.35, 173.15 (C=O); HRMS (ESI): *m*/*z* calcd for C_25_H_26_N_2_O_6_ 468.1856 [M+H_2_O]^+^, found 468.2130.

#### 3.1.10. 1,1′-(Methylenebis(4,1-phenylene))bis(5-oxopyrrolidine-3-carbohydrazide) (34)

To a solution of methyl 1-[4-({4-[4-(methoxycarbonyl)-2-oxopyrrolidine-1-il]phenyl}methyl)phenyl]-5-oxopyrrolidine-3-carboxylate (**33**) (9 g, 20 mmol) in hot DMSO (30 mL), hydrazine hydrate (3.5 mL, 72 mmol) was added. Already after 10 min crystals started forming. The reaction mixture was cooled down. The precipitate was filtered off, washed with diethyl ether, and recrystallized from DMF/H_2_O mixture. Yield 67% (6.05 g); white crystals; m.p. >300 °C decomposes. IR (KBr) ν_max_ (cm^−1^): 1644, 1728 (C=O), 2820–3433 (NH); ^1^H NMR (400 MHz, DMSO-*d_6_*): δ 2.57–2.89 (m, 4H, CH_2_CO), 3.39 (s, 2H, CH), 3.78–4.13 (m, 6H, CH_2_N+CH_2_), 7.21 (d, 4H, *J* = 7.2 Hz, H_Ar_), 7.53 (d, 4H, *J* = 7.2 Hz, H_Ar_), 9.79 (s, 4H, NH_2_), 11.35 (s, 2H, NH); ^13^C NMR (101 MHz, DMSO-*d_6_*): δ 33.97 (*CH_2_*CO), 35.41 (CH), 39.8 (CH_2_), 39.94 (CH_2_), 50.33 (NCH_2_), 119.85, 129.06, 137.22, 137.48 (C_Ar_), 171.49, 172.03 (C=O); Anal. Calcd for C_23_H_26_N_6_O_4_: C, 61.31; H, 5.82; N, 18.66, found: C, 61.47; H, 5.67; N, 18.83 %.

#### 3.1.11. (*N*′,*N*‴)-1,1′-(methylenebis(4,1-phenylene))bis(5-oxo-*N*′-(2-oxoindolin-3-ylidene)pyrrolidine-3-carbohydrazide) (**35**)

Prepared from 1,1′-(methylenebis(4,1-phenylene))bis(5-oxopyrrolidine-3-carbohydrazide) (**34**) (2.25 g) according to the synthesis procedure for compounds **18**–**22**. Yield 54% (1.9 g); yellow crystals; m.p. 191–192 °C; IR (KBr) ν_max_ (cm^−1^): 1665, 1694, 1734 (C=O), 3398, 3451 (NH); ^1^H NMR (400 MHz, DMSO-*d_6_*): δ 2.54–2.64 (m, 3H, CH_2_CO), 3.44 (quint, 1H, *J* = 8.0 Hz, CH), 3.67 (s, 2H, CH_2_), 3.89–4.16 (m, 6H, NCH_2_+CH_2_CO+CH), 6.87–6.97 (m, 1.5H, H_Ar_), 7.02–7.12 (m, 1.5H, H_Ar_), 7.21 (d, 4H, *J* = 8.0 Hz, H_Ar_), 7.37 (t, 1H, *J* = 8.0 Hz, H_Ar_), 7.45–7.66 (m, 8H, H_Ar_), 10.19, 10.82, 11.03, 11.26, 12.59, 13.07 (6s, 4H, NHCO+NH_isatin_); ^13^C NMR (101 MHz, DMSO-*d_6_*): δ 32.34 (*CH_2_*CO), 34.94, 34.96 (CH), 39.83, 40.43 (CH_2_), 49.75, 52.15 (NCH_2_), 111.13, 112.19, 117.82, 119.65, 120.85, 122.57, 122.75, 124.68, 128.84, 131.65, 137.08, 137.14, 137.22, 138.36, 142.49, 150.71 (C_Ar_), 159.37, 162.47, 171.30, 173.10 (C=O); HRMS (ESI): *m*/*z* calcd for C_39_H_32_N_8_O_6_ 712.2761 [M+4H]^+^, found 712.234.

#### 3.1.12. 5-Oxo-1-(4-(phenylamino)phenyl)pyrrolidine-3-carboxylic acid (**36**)

To a solution of itaconic acid (26 g, 0.2 mol) in water (250 mL), 4-aminodiphenylamine (36.80 g, 0.2 mol) was added and the reaction mixture was heated at reflux for 24 h. Precipitate was filtered off and dissolved in an aqueous 20% NaOH solution. The solution was heated at reflux with activated carbon for 5 min, filtered, and acidified with conc. HCl to pH 2. Yield 87% (51.30 g); grey crystals; m.p. 168–169 °C; IR (KBr) ν_max_ (cm^−1^): 1677, 1696 (C=O), 3284 (NH); ^1^H NMR (400 MHz, DMSO-*d_6_*): δ 2.60–2.81 (m, 2H, CH_2_CO); 3.29–3.37 (m, 1H, CH), 3.87–4.05 (m, 2H, N*CH_2_*), 6.79 (t, 1H, *J* = 7.2 Hz, H_Ar‘4_), 6.98–7.12 (m, 4H, H_Ar, Ar‘_), 7.21 (t, 2H, *J* = 7.2 Hz, H_Ar‘3,5_), 7.49 (d, 2H, *J* = 8.8 Hz, H_Ar3,5_), 8.17 (s, 1H, NH), 12.76 (s, 1H, OH); ^13^C NMR (101 MHz, DMSO-*d_6_*): δ 35.08 (*CH_2_*CO), 35.25 (CH), 50.26 (NCH_2_), 116.23, 117.18, 119.43, 121.08, 129.22, 131.76, 139.85, 143.66 (C_Ar_), 171.15, 174.39 (C=O); HRMS (ESI): *m*/*z* calcd for C_17_H_16_N_2_O_3_ 297.1240 [M+H]^+^, found 297.1242.

#### 3.1.13. Methyl 5-oxo-1-(4-(phenylamino)phenyl)pyrrolidine-3-carboxylate (**37**)

To a solution of 5-oxo-1-(4-(phenylamino)phenyl)pyrrolidine-3-carboxylic acid (**36**) (51 g, 0.17 mol) in methanol (120 mL), H_2_SO_4_ (2 mL) was added dropwise. The reaction mixture was heated at reflux for 24 h. Aqueous 15% Na_2_CO_3_ solution was added until pH 8, and the precipitate was filtered off and dried. Yield 72 % (38.58 g); grey crystals; m.p. 80–81 °C; IR (KBr) ν_max_ (cm^−1^): 1651, 1687 (C=O), 3213 (NH); ^1^H NMR (400 MHz, DMSO-*d_6_*): δ 2.64–2.82 (m, 2H, CH_2_CO), 3.40–3.50 (m, 1H, CH), 3.68 (s, 3H, CH_3_), 3.90–4.06 (m, 2H, CH_2_N), 6.79 (t, 1H, *J* = 8.0 Hz, H_Ar‘4_), 6.99–7.12 (m, 4H, H_Ar+Ar‘_), 7.21 (t, 2H, *J* = 8.0 Hz, H_Ar‘3,5_), 7.48 (d, 2H, *J* = 8.4 Hz, H_Ar3,5_), 8.14 (s, 1H, NH); ^13^C NMR (101 MHz, DMSO-*d_6_*): δ 34.87 (*CH_2_*CO), 35.00 (CH), 50.02 (NCH_2_), 52.20 (CH_3_), 116.25, 117.17, 119.44, 121.12, 129.21, 131.62, 139.92, 143.62 (C_Ar_), 170.87, 173.26 (C=O); HRMS (ESI): *m*/*z* calcd for C_18_H_18_N_2_O_3_ 311.1396 [M+H]^+^, found 311.1404.

#### 3.1.14. 5-Oxo-1-(4-(phenylamino)phenyl)pyrrolidine-3-carbohydrazide (**38**)

To a solution of methyl 5-oxo-1-(4-(phenylamino)phenyl)pyrrolidine-3-carboxylate (**37**) (10 g, 32 mmol) in propan-2-ol (25mL), hydrazine hydrate (3 mL, 96 mmol) was added dropwise, and the reaction mixture was heated at reflux for 22 h. Precipitate was filtered off and recrystallized from propan-2-ol. Yield 82 % (8.21 g); grey crystals; m.p. 163–164 °C; IR (KBr) ν_max_ (cm^−1^): 3299–3028 (NH); 1680, 1644 (C=O); ^1^H NMR (400 MHz, DMSO-*d_6_*): δ 2.55–2.72 (m, 2H, CH_2_CO), 3.090–3.21 (m, 1H, CH), 3.77–3.85 (m, 1H, CH_2_N), 3.89–3.97 (m, 1H, CH_2_N), 4.31, 4.47 (2s, 2H, NH*NH_2_*), 6.79 (t, 1H, *J* = 7.2 Hz, H_Ar‘4_), 6.99–7.12 (m, 4H, H_Ar2,6_ + H_Ar‘2,6_), 7.21 (t, 2H, *J* = 7.2 Hz, H_Ar‘3,5_), 7.49 (d, 2H, *J* = 8.8 Hz, H_Ar3,5_), 8.13 (s, 1H, NH), 9.27 (s, 1H, *NH*NH_2_); ^13^C NMR (101 MHz, DMSO-*d_6_*): δ 34.16 (CH), 35.57 (NCO*CH_2_*), 50.92 (N*CH_2_*CH), 116.21, 117.19, 119.38, 120.97, 121.17, 121.22, 129.17, 131.80, 139.77, 143.66 (C_Ar_), 171.34, 171.63 (C=O); HRMS (ESI): *m*/*z* calcd for C_17_H_18_N_4_O_2_ 311.1508 [M+H]^+^, found 311.1503.

#### 3.1.15. 5-Oxo-*N*′-(2-oxoindolin-3-ylidene)-1-(4-(phenylamino)phenyl)pyrrolidine-3-carbohydrazide (**39**)

Prepared from 5-oxo-1-(4-(phenylamino)phenyl)pyrrolidine-3-carbohydrazide (**38**) according to the synthesis procedure for compounds **12**–**17**. Yield 89% (1.95 g); yellow crystals; m.p. 243–244 °C; IR (KBr) ν_max_ (cm^−1^): 1640, 1695, 1725 (C=O), 3175, 3282, 3310 (NH); ^1^H NMR (400 MHz, DMSO-*d_6_*): δ 2.74–2.94 (m, 2H, CH_2_CO), 3.37 (s, 1H, CH), 3.96–4.18 (m, 2H, N*CH_2_*), 6.79 (t, 1H, *J* = 8.0 Hz, H_Ar_), 6.90–6.96 (m, 1H, H_Ar_), 7.03–7.10 (m, 5H, H_Ar_), 7.21 (t, 2H, *J* = 8.0 Hz, H_Ar_), 7.36–7.62 (m, 4H, H_Ar_), 8.14 (s, 1H, NH), 10.83 (s, 0.7H, NHCO), 11.27, 11.37 (s, 1H, NH_isatin_), 12.60, 13.09 (2s, 0.3H, NHCO); ^13^C NMR (101 MHz, DMSO-*d_6_*): δ 34.50 (CH), 35.47 (NCO*CH_2_*), 50.01 (N*CH_2_*CH), 110.63, 111.14, 115.21, 116.23, 117.18, 119.40, 120.83, 121.17, 121.22, 121.69, 122.57, 126.13, 129.17, 131.66, 131.71, 139.89, 139.92, 142.49, 143.64, 143.86 (C_Ar_), 162.48, 164.56, 171.10 (C=O); HRMS (ESI): *m*/*z* calcd for C_25_H_21_N_5_O_3_ 440.1723 [M+H]^+^, found 440.1718.

### 3.2. Pharmacology

#### 3.2.1. Anticancer Activity

##### Cell Culturing

The human malignant melanoma cell line A375 and human colon adenocarcinoma cell line HT-29 were obtained from the American Type Culture Collection (ATCC, Manassas, VA, USA). Human foreskin fibroblasts (HF) CRL-4001 were originally obtained from ATCC and kindly provided by Prof. Helder Santos (University of Helsinki, Finland). A375, HT-29, and HF were cultured in Dulbecco’s modified eagle’s GlutaMAX medium (Gibco (Carlsbad, CA, USA)). Medium was supplemented with 10,000 U/mL penicillin, 10 mg/mL streptomycin (Gibco), and 10% fetal bovine serum (Gibco). Cell cultures were grown at 37 °C in a humidified atmosphere containing 5% CO_2_. They were used until the passage of 20 min.

##### Cell Viability Assay

The effects of the isatin derivatives on cell viability were studied using 3-(4,5-dimethylthiazol-2-yl)-2,5-diphenyltetrazolium bromide (MTT; Sigma-Aldrich Co., St Louis, MO, USA) assays, as described elsewhere [35,89]. Briefly, A375, HT-29, and HF cells were seeded in 96-well plates (Corning) in triplicate repeats at a volume of 100 μL (5 × 10^3^ cells/well). After 24 h of incubation, the cells were treated with 100 μM of tested compounds. After 72 h, the cells were exposed to the reagent MTT for 4 h. Then the medium was aspirated and the formed formazan crystals were dissolved in 100 μL DMSO (Sigma-Aldrich Co.). The absorbance was measured at 570 and 630 nm using a multi-detection microplate reader. The compound’s effect on cell viability was calculated using a formula:

(1) $$Relative\;cell\;viability\;\left(\%\right)=\frac{A-A_{0}}{A_{NC}-A_{0}}$$

where *A*—mean of absorbance of tested compound; *A*_0_—mean of absorbance of blank (no cells, positive control); *A_NC_*—mean of absorbance of negative control (only cells, no treatment).

The EC_50_ values of the most active compounds, **17**, **18**, **20**, and **35**, were established by the same MTT procedure; only the compound serial dilutions from 50 to 1.56 µM were made in a medium and added to the cells in triplicate repeats. Each EC_50_ value, which represents the concentration of a compound causing a 50% reduction of cancer cells’ metabolic activity, has been calculated using the Hill equation.

##### Colony Formation Assay

Clonogenic assays were used to evaluate the inhibitory effects of the most active compounds, **17**, **18**, **20**, and **35**, on cell survival and proliferation via forming colonies, as described elsewhere [90]. Briefly, melanoma A375 and colon adenocarcinoma HT-29 cells were seeded into 12-well plates in triplicate repeats (2 × 10^2^ cells/well) and grown in the previously described cell culture medium at 37 °C in a humidified atmosphere containing 5% CO_2_. After 24 h, the fresh media containing compounds **17**, **18**, **20**, and **35** at concentrations representing 50% of calculated EC_50_ values were added to cells. Then, cells were incubated at 37 °C in a humidified atmosphere containing 5% CO_2_ for the next 7–8 days. Cells treated with medium containing 0.5% DMSO served as a negative control. After incubation, the colonies were stained with a 0.1% crystal violet (Sigma-Aldrich Co.) solution. First, the media were removed from cells, and the cells were washed with sterile PBS. Then, the cells were fixed in a 4% formaldehyde (Thermo Scientific) solution and washed with PBS two times to remove the fixative, and stained with crystal violet for 20 min. After the stain had been removed, the remaining stain residues were washed three times with sterile water. The plates were dried overnight and imaged with the SYNGENE G:BOX gel doc system, using Gen Sys software. Quantification was performed using Gene tools software.

##### Compound Activity in Spheroids

Cancer cell spheroids were formed by using the magnetic 3D Bioprinting method, as described elsewhere [91]. Briefly, melanoma (A375) and adenocarcinoma (HT-29) cells and human fibroblasts at 70% confluency in a 6-well plate were incubated with Nanoshuttle (n3D Biosciences, Inc.) for 7–8 h at 37 °C in a humidified atmosphere containing 5% CO_2_. After nanoparticles were taken up by cells, they were trypsinized, centrifuged, and seeded into ultra-low attachment 96-well plate in a volume of 100 µL (1.5 × 10^3^ cancer cells and 1.5 × 10^3^ human fibroblasts/well). The plate was placed on a magnetic drive and incubated for 2 days at 37 °C in a humidified atmosphere containing 5% CO_2_. Then the fresh medium containing 20 or 50 µM of tested compound was added to the wells. The spheroids were captured every two days using the Olympus IX73 inverted microscope (OLYMPUS CORPORATION). The quantitative analysis of compound anticancer activity in spheroids was performed using ImageJ (National Institutes of Health) and Microsoft Office Excel software).

#### 3.2.2. Antioxidant Activity

The FRAP (ferric reducing antioxidant power) assay is based on the reduction of the colorless Fe^3+^-2,4,6-tripyridyl-*s*-triazine complex to the intensely blue Fe^2+^-2,4,6-tripyridyl-*s*-triazine complex in acidic medium. FRAP values are calculated from increasing absorbances measured at 593 nm. The FRAP reagent contained 2.5 mL of a 10 mM TPTZ (2,4,6-tripyridyl-*s*-triazine) solution in 40 mM HCl, along with 2.5 mL of FeCl_3_ (20 mM) and 25 mL of acetate buffer (0.3 M, pH = 3.6). For each compound, 100 μL of it (20 mM) was mixed with 3 mL of the FRAP reagent. The absorbance of the reaction mixture at 593 nm was measured spectrophotometrically. For comprising the calibration curve, five concentrations of FeSO_4_∙7H_2_O (5, 10, 15, 20, and 25 μM) were used, and the absorbances of the samples’ solutions were then measured [92]. Each experiment was conducted in triplicate.

#### 3.2.3. Statistical Analysis

All biological experiments were repeated at least three times. The mean and standard deviation are reported. The data were processes using Microsoft Office Excel 2016 software (Microsoft Corporation, Redmond, WA, USA). Statistical analysis was performed by using Student’s t-tests. The level of significance was set as *p* < 0.05.

## 4. Conclusions

In conclusion, a series of novel hydrazone-isatin derivatives were synthesized and evaluated for their anticancer and antioxidant properties. In the anticancer activity assay, the colon adenocarcinoma HT-29 cell line appeared to be more sensitive to the treatment with the hydrazone-isatin derivatives, compared to the malignant melanoma A375 cell line. Bis(hydrazone-isatins) were found to be more active than their mono analogues. Bis(*N*′-(2-oxoindolin-3-ylidene)propanehydrazide) **20**, bearing the electron-donating methoxy group at the *p*-position of the benzene ring, and diphenylmethane derivative **35**, bearing two 5-oxo-*N*′-(2-oxoindolin-3-ylidene)pyrrolidine-3-carbohydrazide “arms” at *p*-positions of the benzene rings, were the most active among all compounds synthesized. These compounds reduced the colony-forming abilities of both cell lines, and also inhibited the growth and viability of colon cancer and melanoma spheroids.

The same bis(hydrazone-isatins) possessed the highest ferric reducing antioxidant power. These promising results suggest that variously substituted bis(hydrazone-isatins) based on an *N*-substituted β-amino acid scaffold could be developed further as a new class of anticancer agents against aggressive malignant melanoma and colon adenocarcinoma tumors.

## Data Availability

All datasets generated for this study are included in the article.

## Supplementary Materials

The following are available online at https://www.mdpi.com/article/10.3390/ijms22157799/s1. Figures S1–S168 display ^1^H NMR, ^13^C NMR, and HRMS spectra of compounds **12**–**22**, **26**–**28**, **30**, and **32**–**39**.
