# Supplementary material for: Novel N-Substituted Amino Acid Hydrazone-Isatin Derivatives: Synthesis, Antioxidant Activity, and Anticancer Activity in 2D and 3D Models In Vitro
Source: Int J Mol Sci. 2021 Jul 21;22(15):7799. doi: 10.3390/ijms22157799 (PMC8346030; doi:10.3390/ijms22157799)
Supplement: Supplementary file 1 [file ijms-22-07799-s001.zip › ijms-1284787-supplementary.pdf]

Supplementary Material

Novel *N*-Substituted Amino Acid Hydrazone-Isatin Derivatives: Synthesis, Antioxidant Activity, and Anticancer Activity in 2D and 3D Models *In Vitro*

Ingrida Tumosienė[1], Ilona Jonuškienė[1], Kristina Kantminienė[2]*, Vytautas Mickevičius[1] and Vilma Petrikaitė[3,4,5]

[1] Department of Organic Chemistry, Kaunas University of Technology, Radvilėnų pl. 19, LT-50254 Kaunas, Lithuania; ingrida.tumosiene@ktu.lt (I.T.); ilona.jonuskiene@ktu.lt (I.J.); vytautas.mickevicius@ktu.lt (V.M.)
[2] Department of Physical and Inorganic Chemistry, Kaunas University of Technology, Radvilėnų pl. 19, LT-50254 Kaunas, Lithuania; kristina.kantminiene@ktu.lt
[3] Laboratory of Drug Targets Histopathology, Institute of Cardiology, Lithuanian University of Health Sciences, Sukilėlių pr. 13, LT-50162 Kaunas, Lithuania; vilma.petrikaite@lsmuni.lt
[4] Institute of Physiology and Pharmacology, Faculty of Medicine, Lithuanian University of Health Sciences, A. Mickevičiaus g. 9, LT-44307 Kaunas, Lithuania; vilma.petrikaite@lsmuni.lt
[4] Institute of Biotechnology, Life Sciences Center, Vilnius University, Saulėtekio al. 7, LT-10257 Vilnius, Lithuania; vilma.petrikaite@lsmuni.lt
* Correspondence: kristina.kantminiene@ktu.lt

Table of contents

Figure S1. ^{1}H NMR (400 MHz, DMSO-d_6) spectrum of **12**

Figure S2. ^{13}C NMR (101 MHz, DMSO-d_6) spectrum of **12**

Figure S3. HRMS spectrum of **12**

Figure S4. ^{1}H NMR (400 MHz, DMSO-d_6) spectrum of **13**

Figure S5. ^{13}C NMR (101 MHz, DMSO-d_6) spectrum of **13**

Figure S6. HRMS spectrum of **13**

Figure S7. ^{1}H NMR (400 MHz, DMSO-d_6) spectrum of **14**

Figure S8. ^{13}C NMR (101 MHz, DMSO-d_6) spectrum of **14**

Figure S9. HRMS spectrum of **14**

Figure S10. ^{1}H NMR (400 MHz, DMSO-d_6) spectrum of **15**

Figure S11. ^{13}C NMR (101 MHz, DMSO-d_6) spectrum of **15**

Figure S12. HRMS spectrum of **15**

Figure S13. ^{1}H NMR (400 MHz, DMSO-d_6) spectrum of **16**

Figure S14. ^{13}C NMR (101 MHz, DMSO-d_6) spectrum of **16**

Figure S15. HRMS spectrum of **16**

Figure S16. ^{1}H NMR (400 MHz, DMSO-d_6) spectrum of **17**

Figure S17. ^{13}C NMR (400 MHz, DMSO-d_6) spectrum of **17**

Figure S18. HRMS spectrum of **17**

Figure S19. ^{1}H NMR (400 MHz, DMSO-d_6) spectrum of **18**

Figure S20. ^{13}C NMR (101 MHz, DMSO-d_6) spectrum of **18**

Figure S21. HRMS spectrum of **18**

Figure S22. ^{1}H NMR (400 MHz, DMSO-d_6) spectrum of **19**

Figure S23. ^{13}C NMR (101 MHz, DMSO-d_6) spectrum of **19**

Figure S24. HRMS spectrum of **19**

Figure S25. ^{1}H NMR (400 MHz, DMSO-d_6) spectrum of **20**

Figure S26. ^{13}C NMR (101 MHz, DMSO-d_6) spectrum of **20**

Figure S27. HRMS spectrum of **20**

Figure S28. ^{1}H NMR (400 MHz, DMSO-d_6) spectrum of **21**

Figure S29. ^{13}C NMR (101 MHz, DMSO-d_6) spectrum of **21**

Figure S30. HRMS spectrum of **21**

Figure S31. ^{1}H NMR (400 MHz, DMSO-d_6) spectrum of **22**

Figure S32. ^{13}C NMR (101 MHz, DMSO-d_6) spectrum of **22**

Figure S33. HRMS spectrum of **22**

Figure S34. ^{1}H NMR (400 MHz, DMSO-d_6) spectrum of **26**

Figure S35. ^{13}C NMR (101 MHz, DMSO-d_6) spectrum of **26**

Figure S36. HRMS spectrum of **26**

Figure S37. ^{1}H NMR (400 MHz, DMSO-d_6) spectrum of **27**

Figure S38. ^{13}C NMR (101 MHz, DMSO-d_6) spectrum of **27**

Figure S39. HRMS spectrum of **27**

Figure S40. ^{1}H NMR (400 MHz, DMSO-d_6) spectrum of **28**

Figure S41. ^{13}C NMR (101 MHz, DMSO-d_6) spectrum of **28**

Figure S42. HRMS spectrum of **28**

Figure S43. ^{1}H NMR (400 MHz, DMSO-d_6) spectrum of **30**

Figure S44. ^{13}C NMR (101 MHz, DMSO-d_6) spectrum of **30**

Figure S45. HRMS spectrum of **30**

Figure S46. ^{1}H NMR (400 MHz, DMSO-d_6) spectrum of **32**

Figure S47. ^{13}C NMR (101 MHz, DMSO-d_6) spectrum of **32**

Figure S48. HRMS spectrum of **32**

Figure S49. ^{1}H NMR (400 MHz, DMSO-d_6) spectrum of **33**

Figure S50. ^{13}C NMR (101 MHz, DMSO-d_6) spectrum of **33**

Figure S51. HRMS spectrum of **33**

Figure S52. ^{1}H NMR (400 MHz, DMSO-d_6) spectrum of **34**

Figure S53. ^{13}C NMR (101 MHz, DMSO-d_6) spectrum of **34**

Figure S54. ^{1}H NMR (400 MHz, DMSO-d_6) spectrum of **35**

Figure S55. ^{13}C NMR (101 MHz, DMSO-d_6) spectrum of **35**

Figure S56. HRMS spectrum of **35**

Figure S57. ^{1}H NMR (400 MHz, DMSO-d_6) spectrum of **36**

Figure S58. ^{13}C NMR (101 MHz, DMSO-d_6) spectrum of **36**

Figure S59. HRMS spectrum of **36**

Figure S60. ^{1}H NMR (400 MHz, DMSO-d_6) spectrum of **37**

Figure S61. ^{13}C NMR (101 MHz, DMSO-d_6) spectrum of **37**

Figure S62. HRMS spectrum of **37**

Figure S63. ^{1}H NMR (400 MHz, DMSO-d_6) spectrum of **38**

Figure S64. ^{13}C NMR (101 MHz, DMSO-d_6) spectrum of **38**

Figure S65. HRMS spectrum of **38**

Figure S66. ^{1}H NMR (400 MHz, DMSO-d_6) spectrum of **39**

Figure S67. ^{13}C NMR (101 MHz, DMSO-d_6) spectrum of **39**

Figure S68. HRMS spectrum of **39**

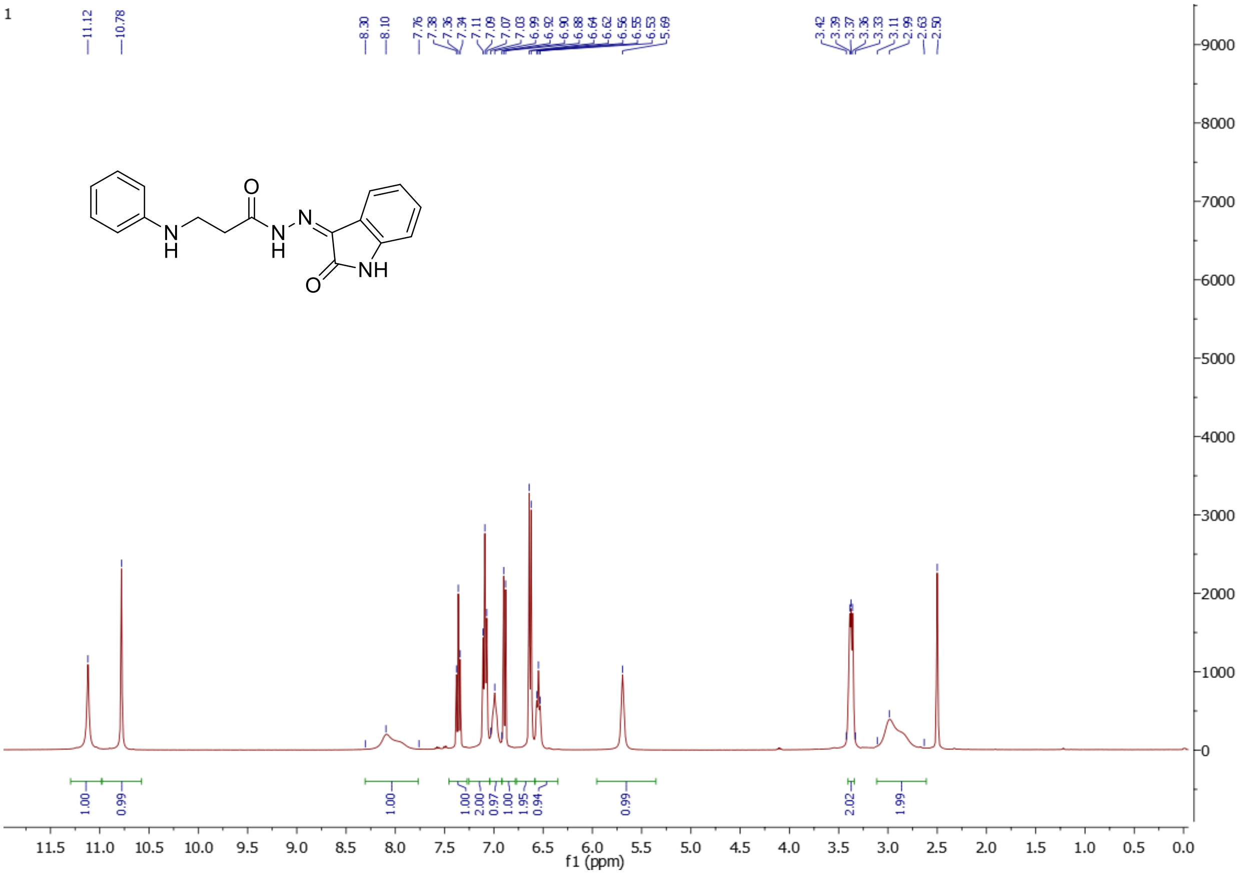

Figure S1. ^{1}H NMR (400 MHz, DMSO-d_6) spectrum of **12**

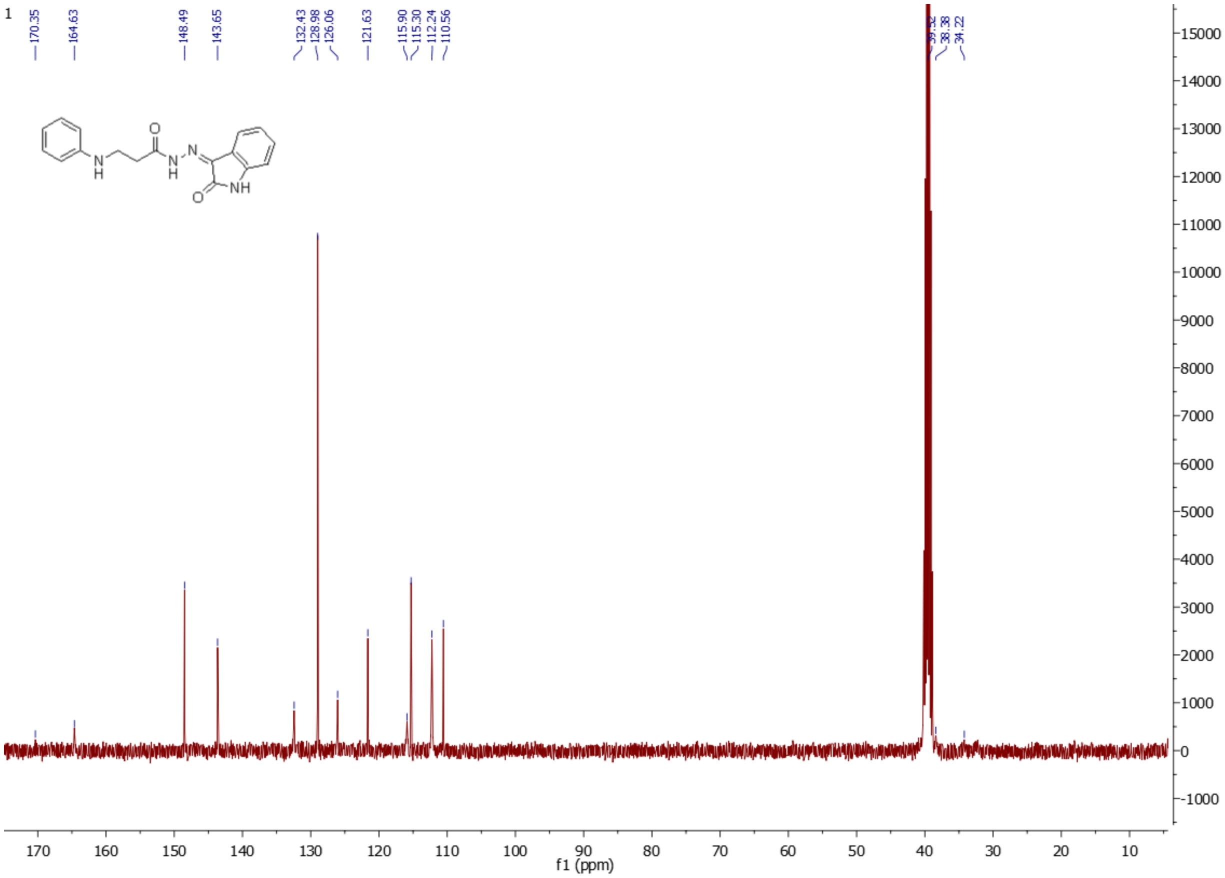

Figure S2. ^{13}C NMR (101 MHz, DMSO-d_6) spectrum of **12**

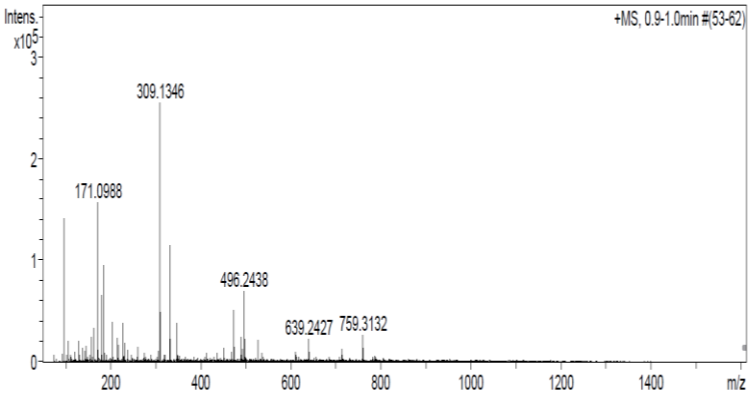

Meas. m/z	#	Formula	Score	m/z	err [ppm]	Mean err [ppm]	mSigma	rdb	e⁻ Conf	N-Rule
309.1346	1	C 17 H 17 N 4 O 2	100.00	309.1346	-0.1	0.2	4.9	11.5	even	ok
	2	C 16 H 21 O 6	45.67	309.1333	-4.4	-4.1	10.0	6.5	even	ok

Figure S3. HRMS spectrum of **12**

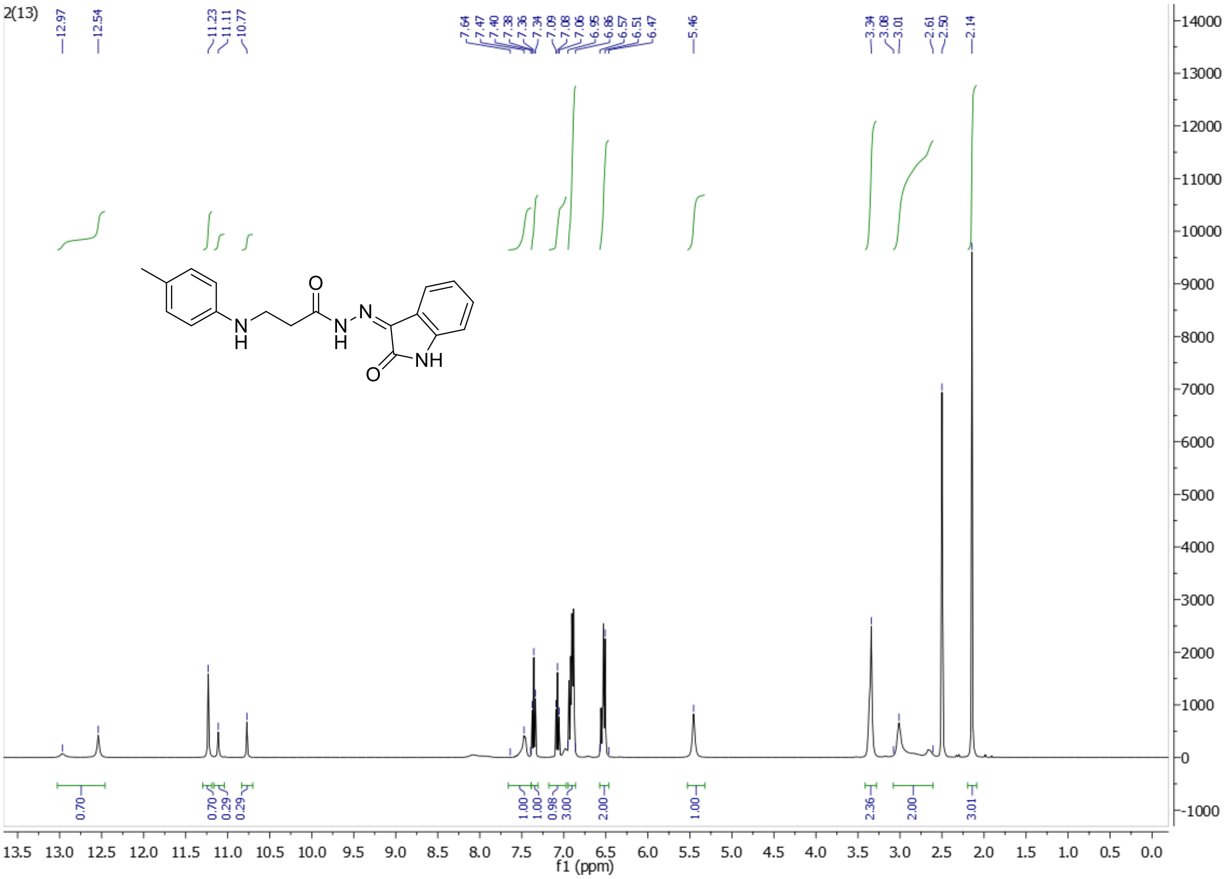

Figure S4. ^{1}H NMR (400 MHz, DMSO-d_6) spectrum of **13**

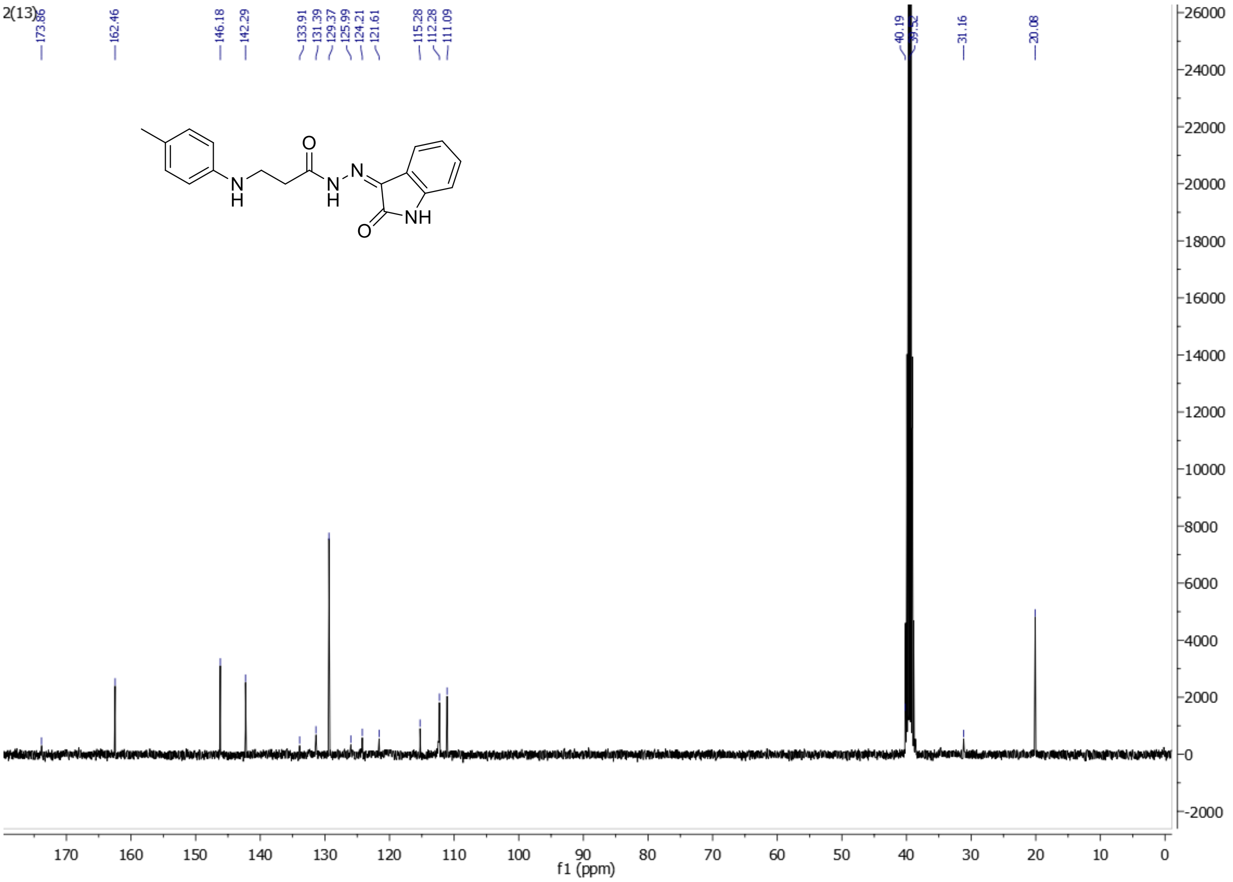

Figure S5. ^{13}C NMR (101 MHz, DMSO-d_6) spectrum of **13**

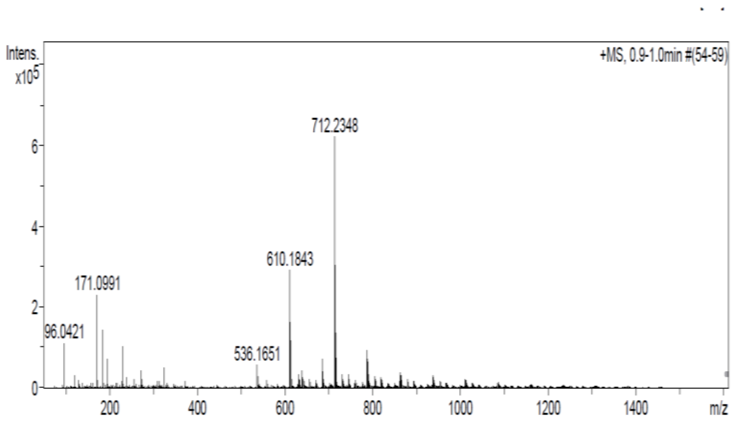

Meas. m/z	#	Formula	Score	m/z	err [ppm]	Mean err [ppm]	mSigma	rdb	e⁻ Conf	N-Rule
323.1501	1	C 17 H 23 O 6	59.58	323.1489	-3.7	-3.5	7.3	6.5	even	ok
	2	C 18 H 19 N 4 O 2	100.00	323.1503	0.5	0.6	7.8	11.5	even	ok

Figure S6. HRMS spectrum of **13**

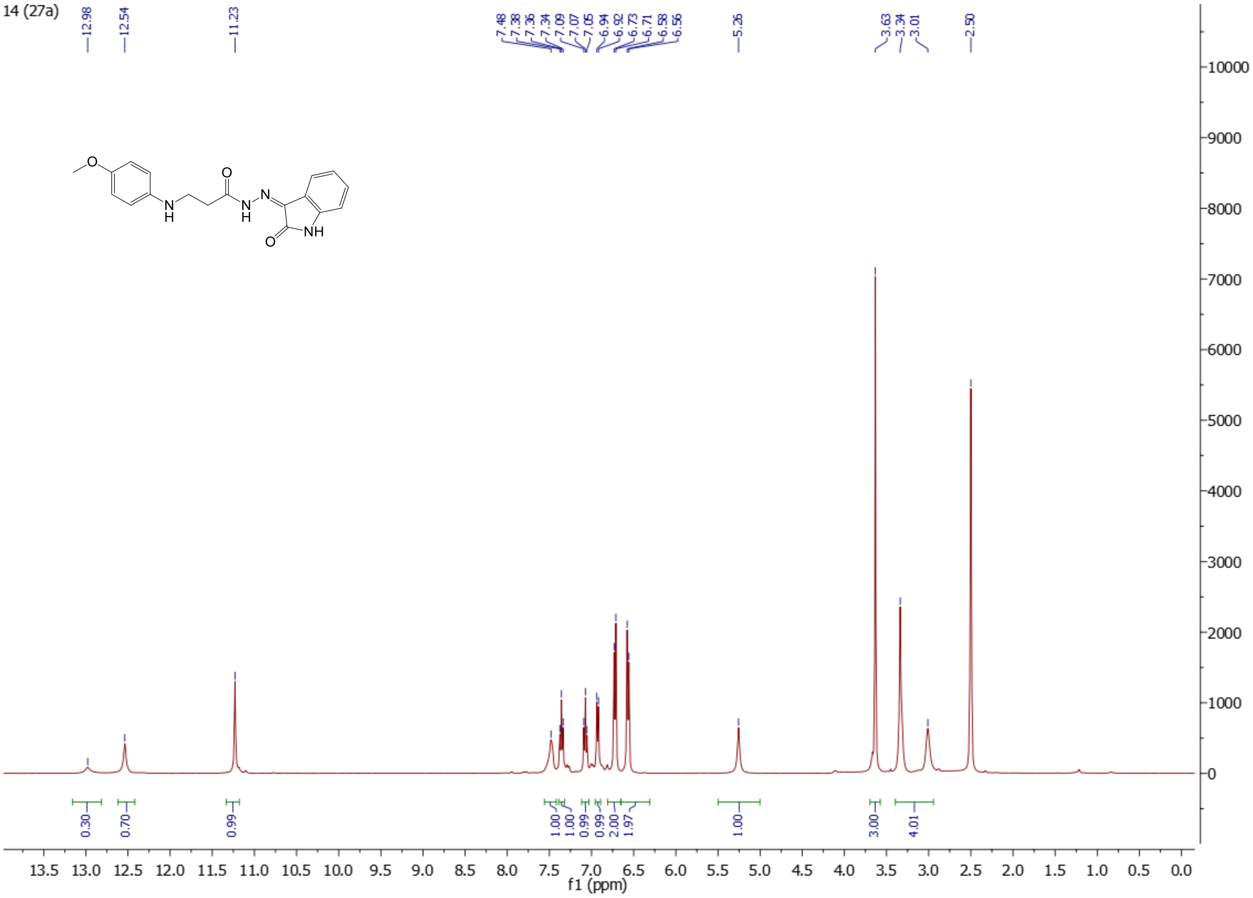

Figure S7. ^{1}H NMR (400 MHz, DMSO-d_6) spectrum of **14**

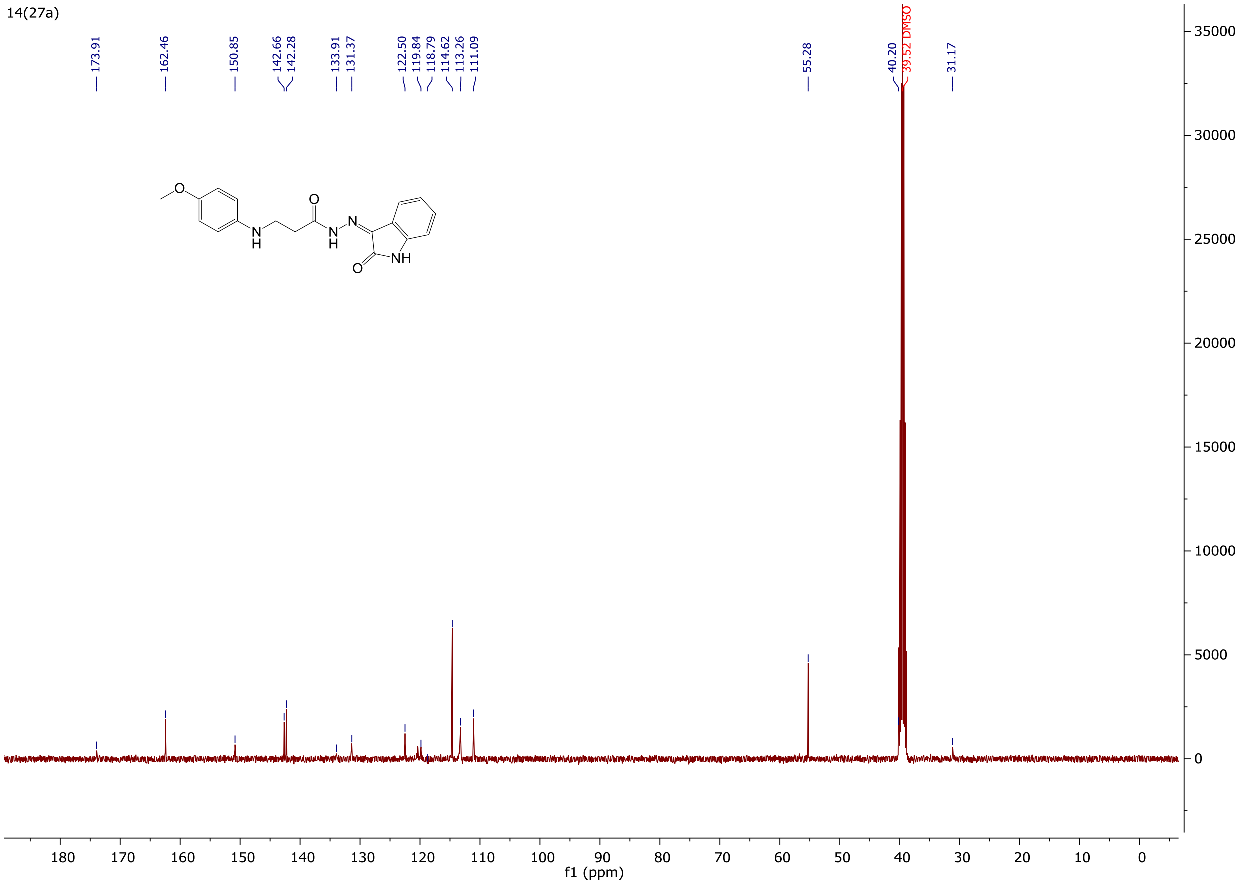

Figure S8. ^{13}C NMR (101 MHz, DMSO-d_6) spectrum of **14**

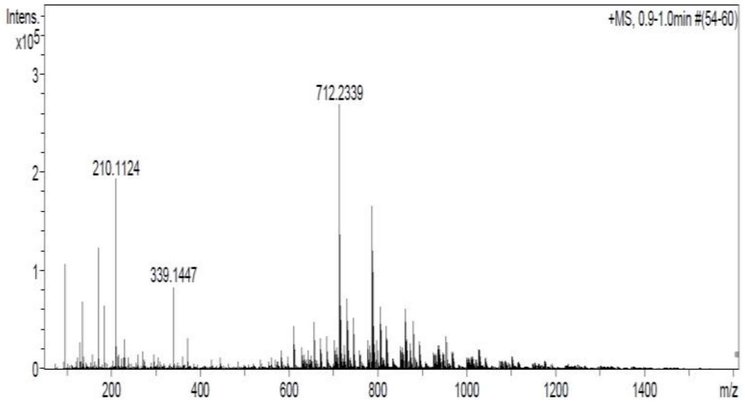

Meas. m/z	#	Formula	Score	m/z	err [ppm]	Mean err [ppm]	mSigma	rdb	e⁻ Conf	N-Rule
339.1447	1	C 18 H 19 N 4 O 3	100.00	339.1452	1.3	1.6	3.8	11.5	even	ok
	2	C 17 H 23 O 7	68.30	339.1438	-2.7	-2.3	11.5	6.5	even	ok

Figure S9. HRMS spectrum of **14**

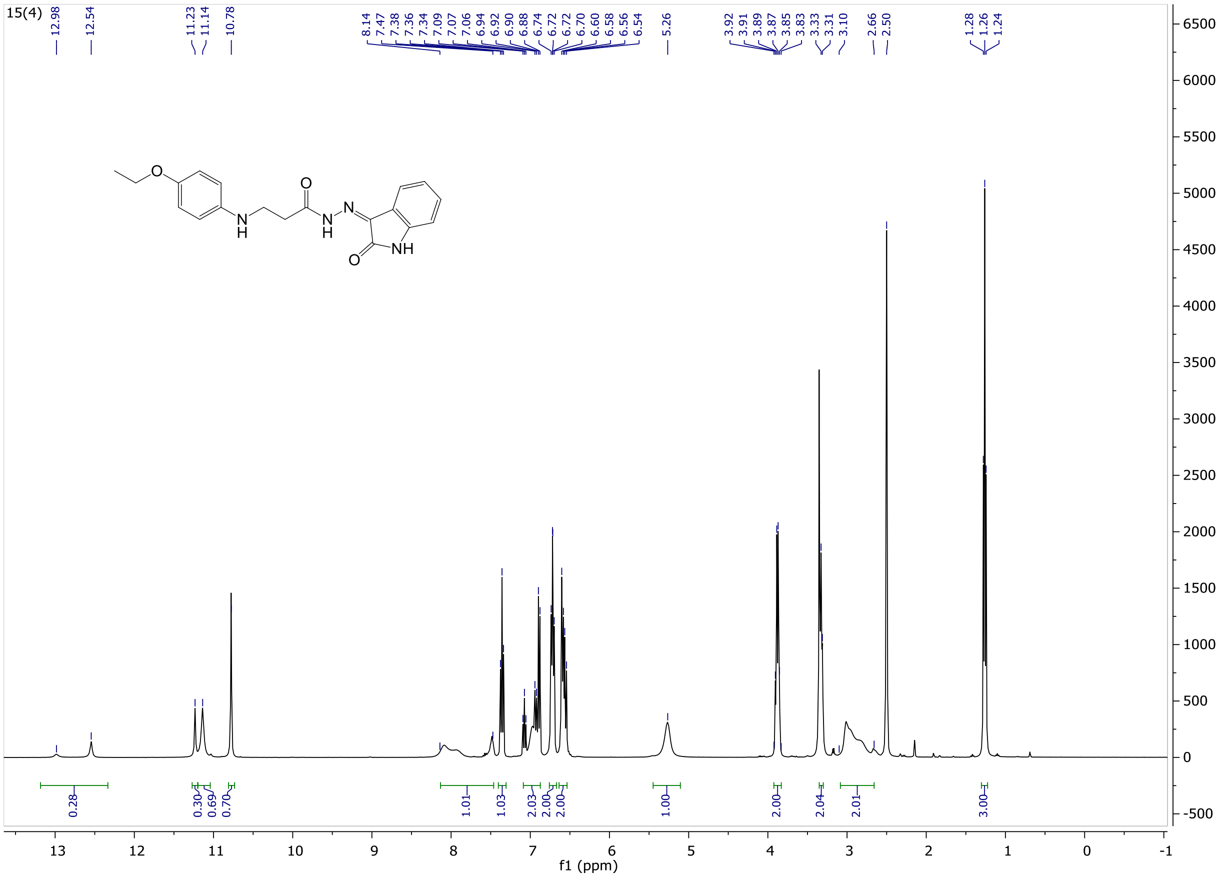

Figure S10. ^{1}H NMR (400 MHz, DMSO-d_6) spectrum of **15**

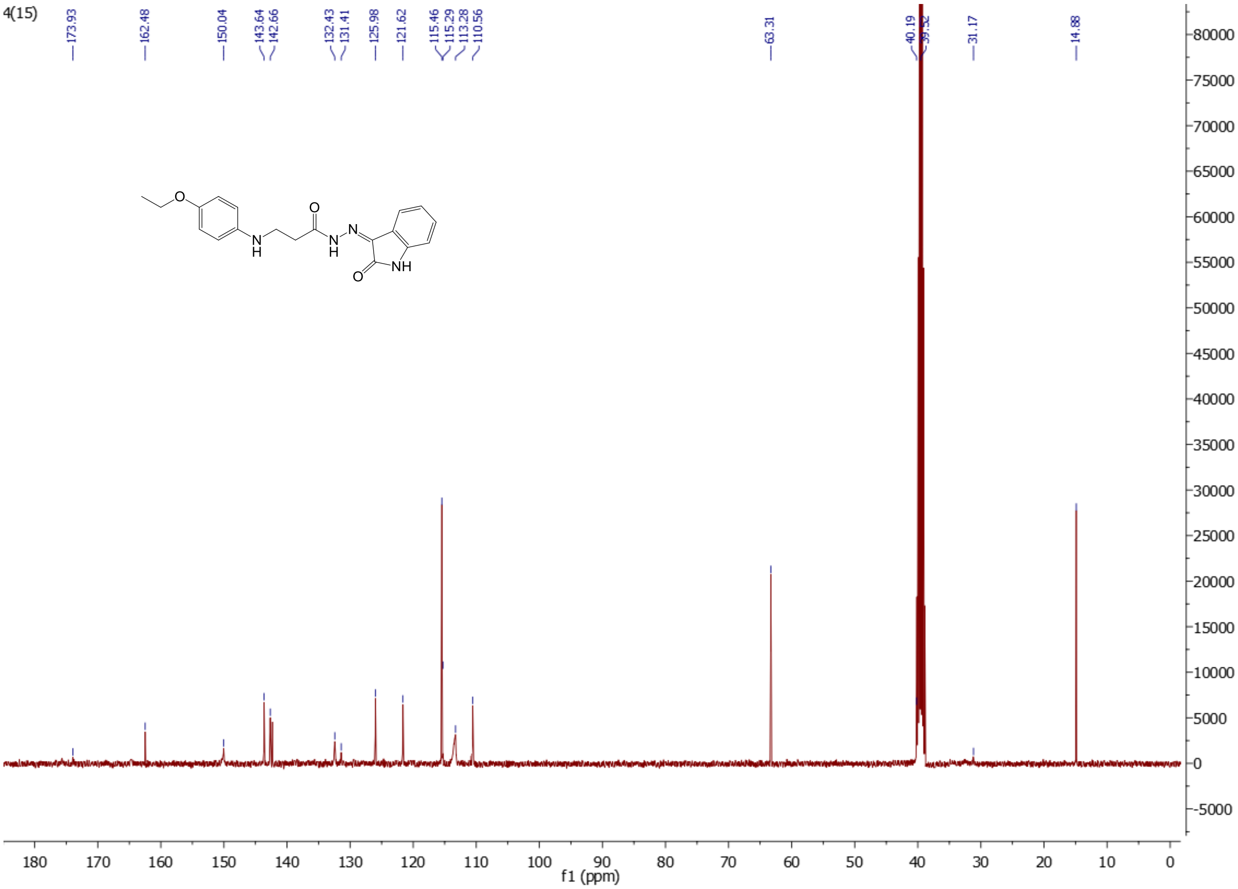

Figure S11. ^{13}C NMR (101 MHz, DMSO-d_6) spectrum of **15**

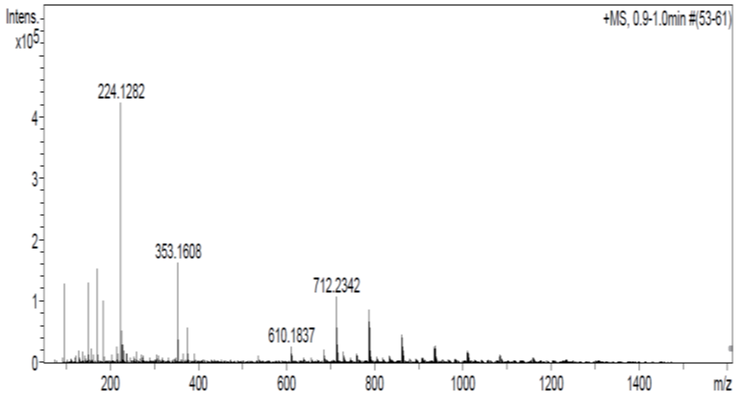

Meas. m/z	#	Formula	Score	m/z	err [ppm]	Mean err [ppm]	mSigma	rdb	e⁻ Conf	N-Rule
353.1608	1	C 19 H 21 N 4 O 3	100.00	353.1608	-0.1	0.2	3.4	11.5	even	ok
	2	C 18 H 25 O 7	38.35	353.1595	-3.9	-3.5	17.6	6.5	even	ok

Figure S12. HRMS spectrum of **15**

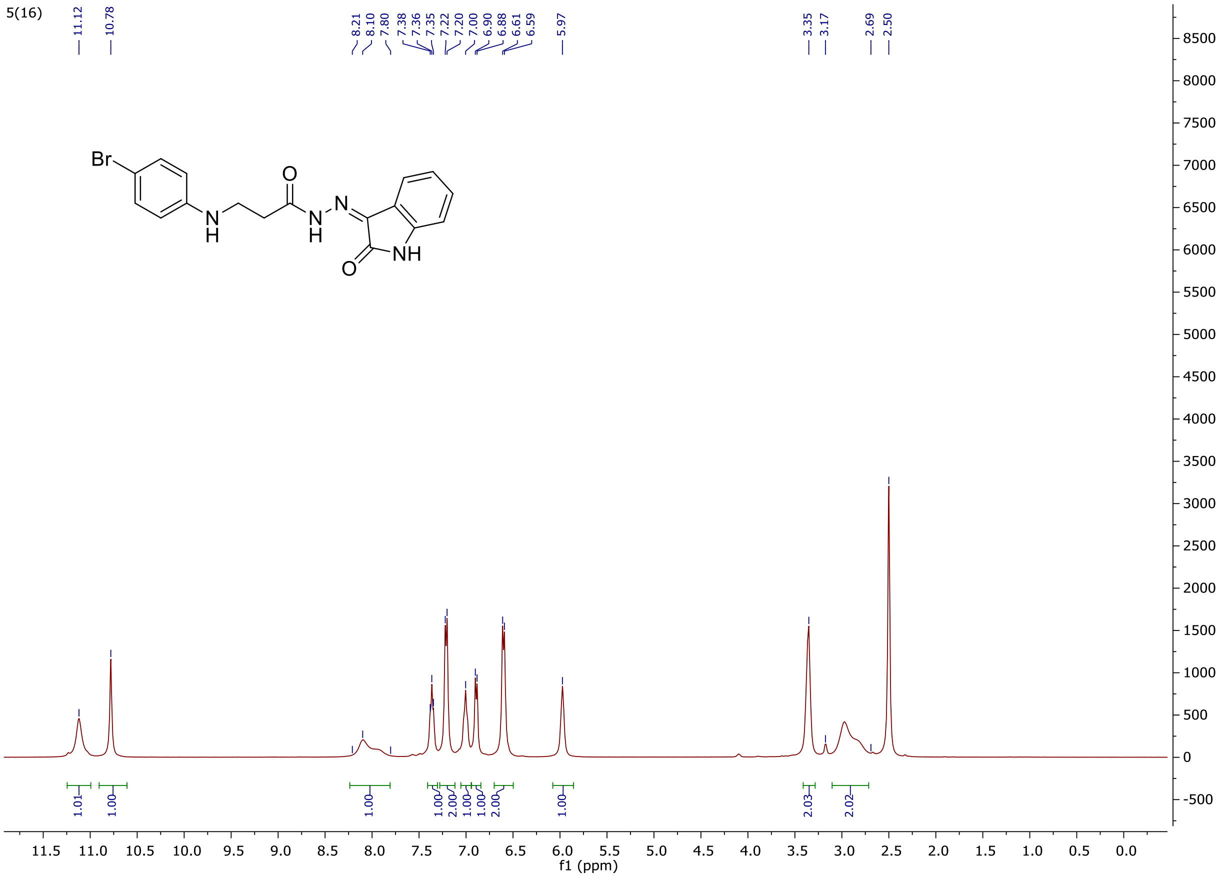

Figure S13. ¹H NMR (400 MHz, DMSO-d_6) spectrum of **16**

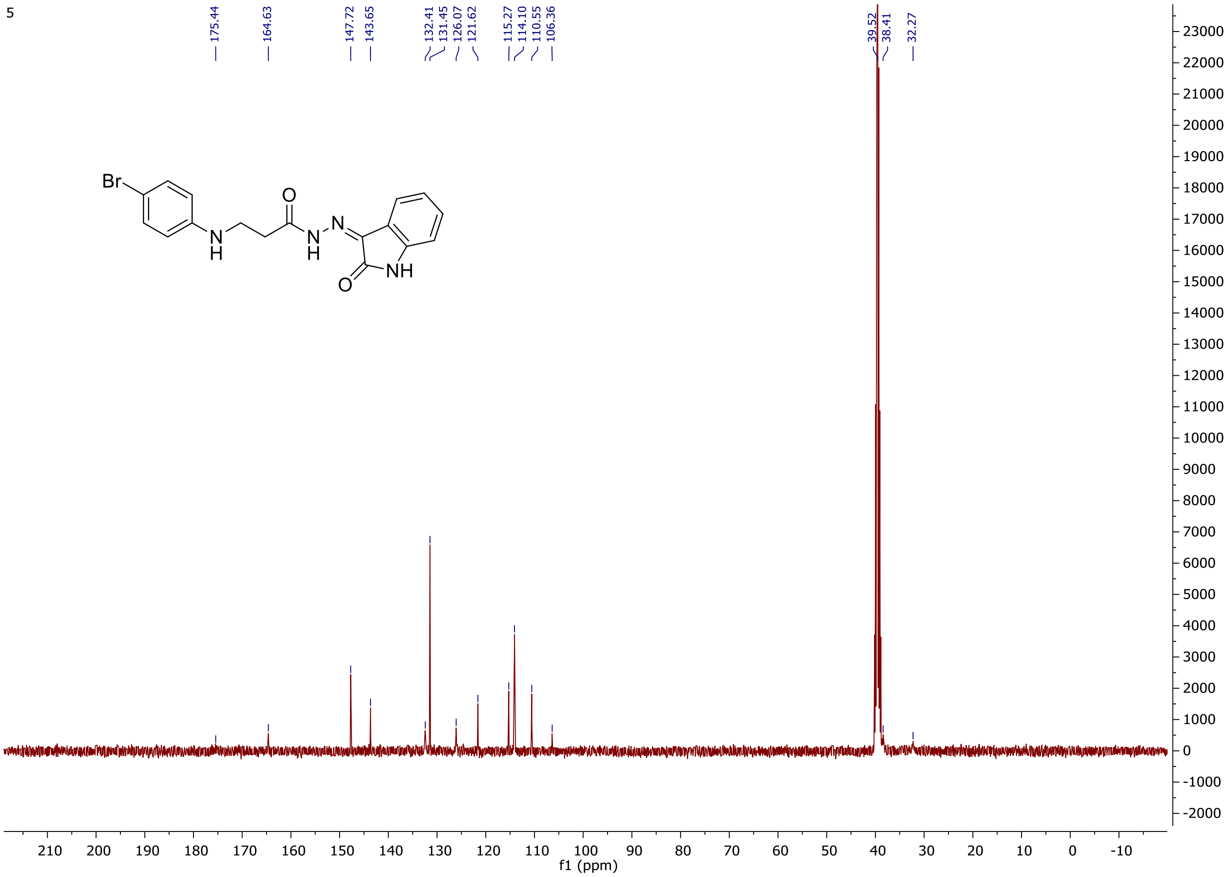

Figure S14. ^{13}C NMR (101 MHz, DMSO-d_6) spectrum of **16**

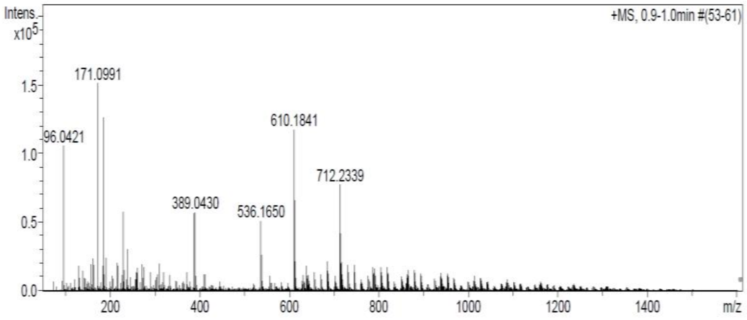

Meas. m/z	#	Formula	Score	m/z	err [ppm]	Mean err [ppm]	mSigma	rdb	e⁻ Conf	N-Rule
387.0449	1	C 17 H 16 Br N 4 O 2	100.00	387.0451	0.5	0.6	5.4	11.5	even	ok
	2	C 14 H 3 N 12 O 3	77.21	387.0446	-1.0	-0.8	14.9	19.5	even	ok
	3	C 16 H 20 Br O 6	48.46	387.0438	-3.0	-2.8	17.6	6.5	even	ok
	4	C 29 H 7 O 2	18.30	387.0441	-2.3	-2.0	59.6	26.5	even	ok
	5	C 17 H 11 N 2 O 9	0.00	387.0459	2.5	9.9	556.8	13.5	even	ok
	6	C 13 H 7 N 8 O 7	0.00	387.0432	-4.4	2.9	561.8	14.5	even	ok

Figure S15. HRMS spectrum of **16**

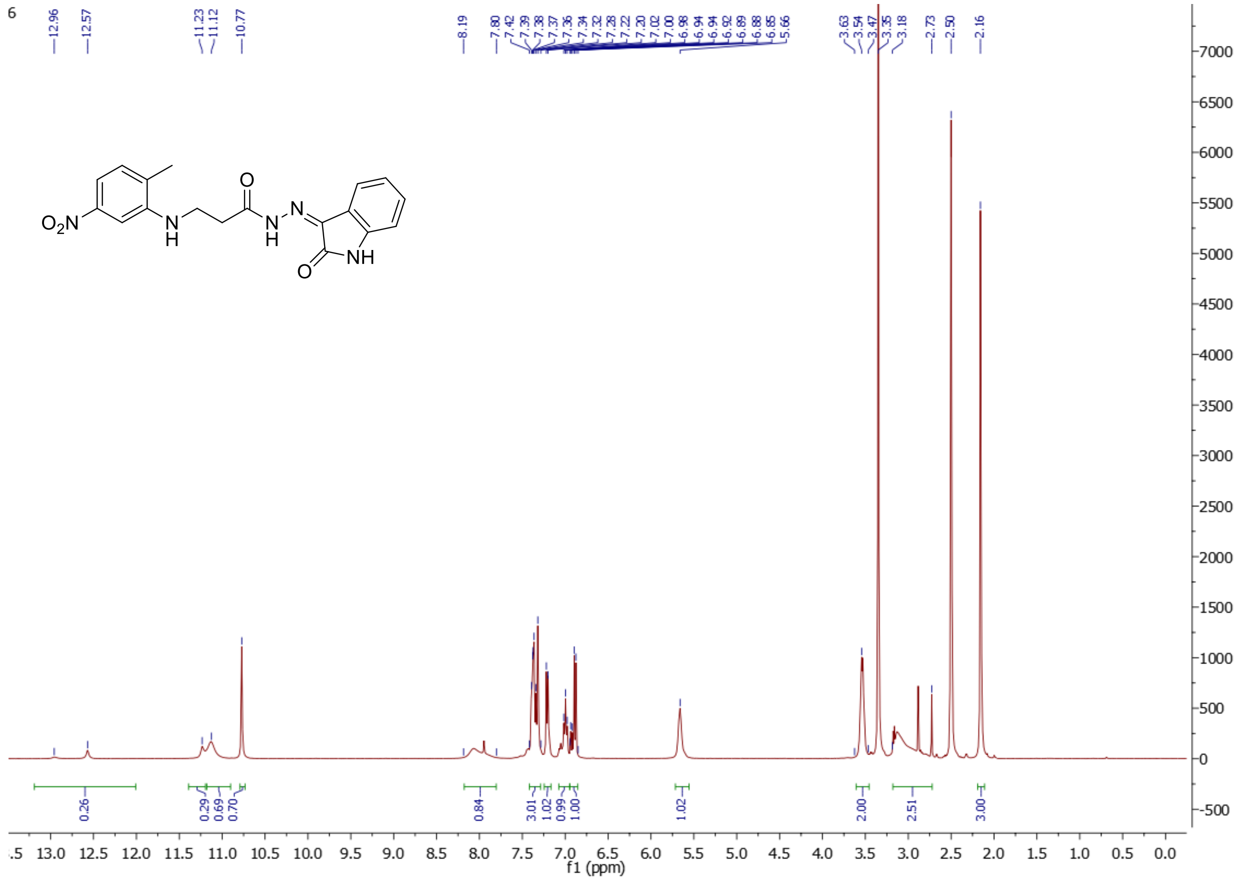

Figure S16. ^{1}H NMR (400 MHz, DMSO-*d*6) spectrum of **17**

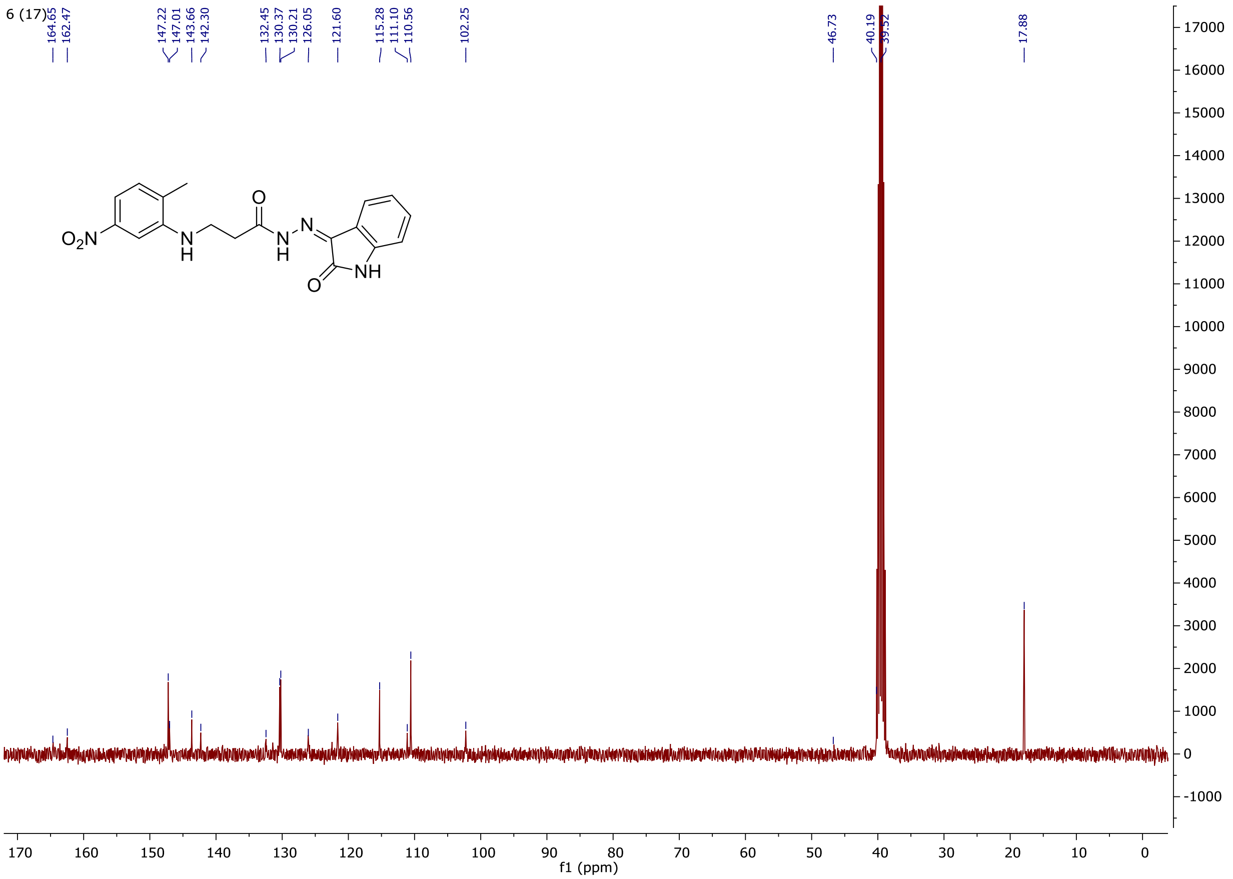

Figure S17. ^{13}C NMR (101 MHz, DMSO-d_6) spectrum of **17**

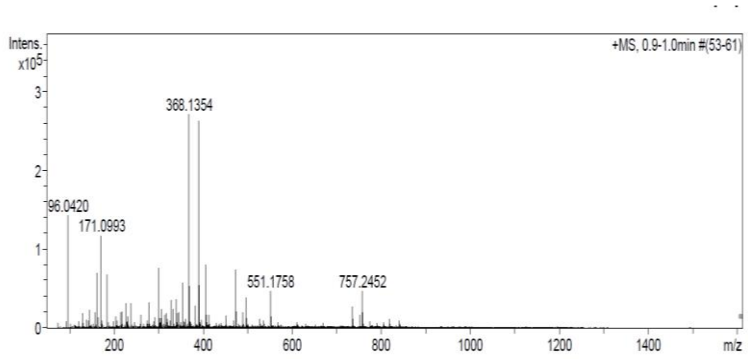

Meas. m/z	#	Formula	Score	m/z	err [ppm]	Mean err [ppm]	mSigma	rdb	e⁻ Conf	N-Rule
368.1354	1	C 17 H 22 N O 8	55.22	368.1340	-3.7	-3.5	4.9	7.5	even	ok
	2	C 18 H 18 N 5 O 4	100.00	368.1353	-0.1	0.2	10.5	12.5	even	ok
	3	C 19 H 14 N 9	38.60	368.1367	3.5	3.8	25.3	17.5	even	ok
390.1173	1	C 19 H 20 N O 8	67.47	390.1183	2.7	3.1	5.1	10.5	even	ok
	2	C 16 H 12 N 11 O 2	100.00	390.1170	-0.7	-0.4	5.2	16.5	even	ok
	3	C 15 H 16 N 7 O 6	43.03	390.1157	-4.2	-3.9	10.1	11.5	even	ok

Figure S18. HRMS spectrum of **17**

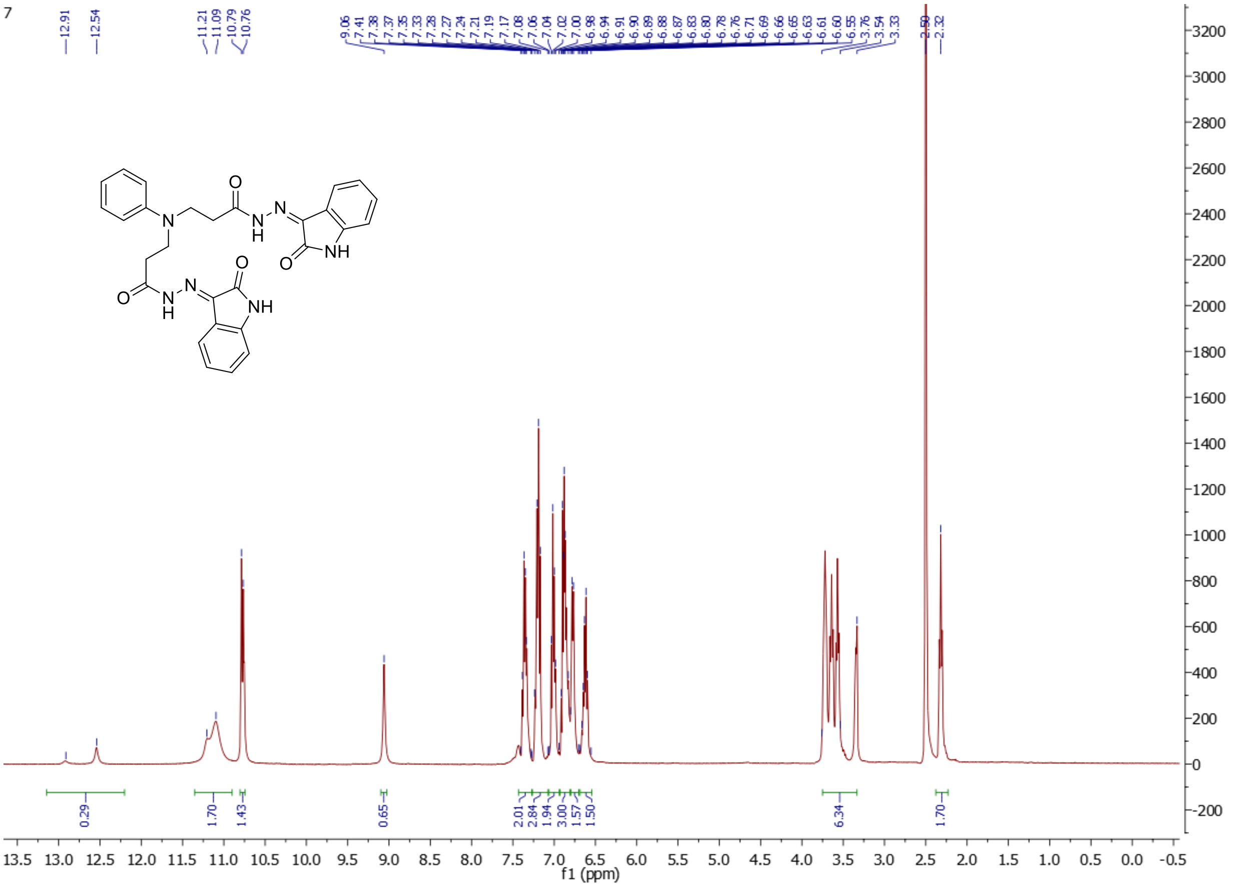

Figure S19. ^{1}H NMR (400 MHz, DMSO-d_6) spectrum of **18**

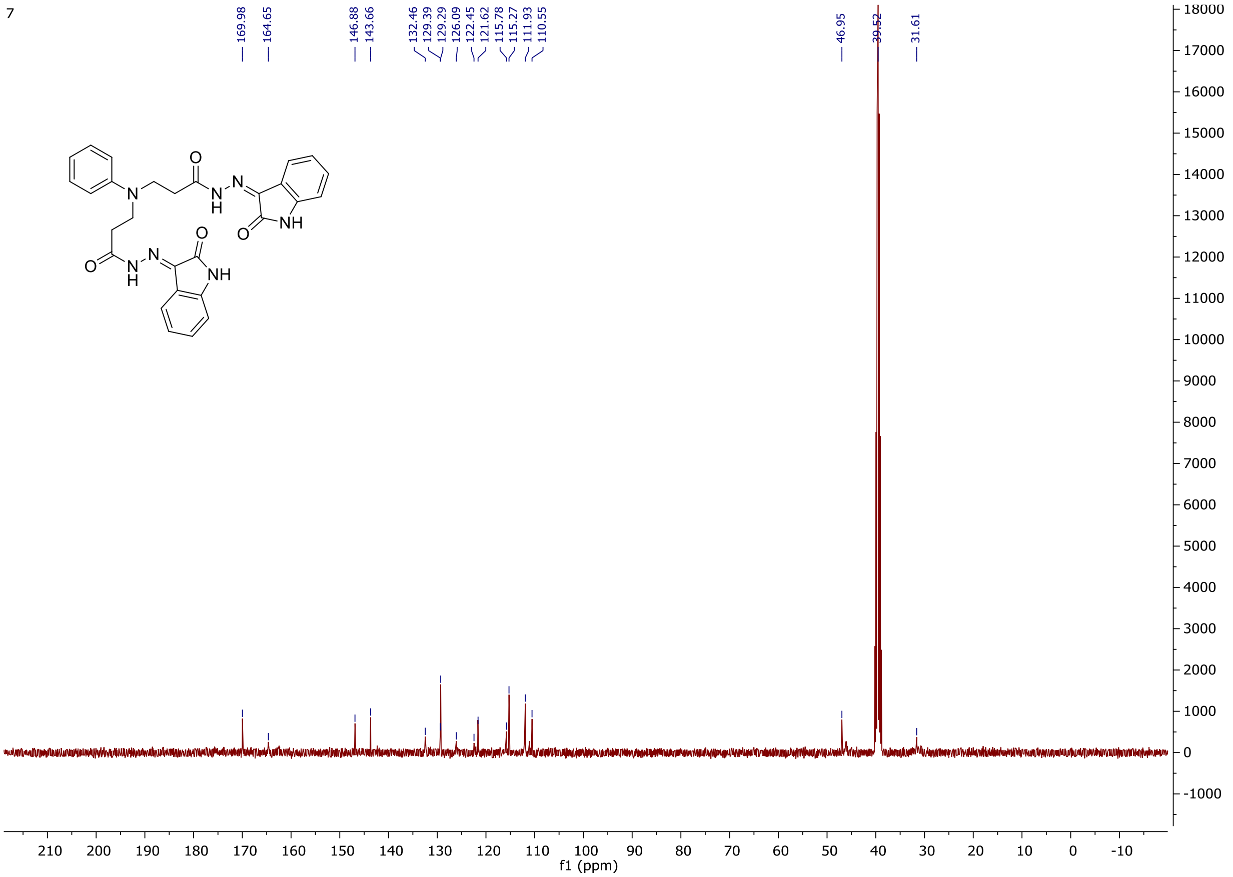

Figure S20. ^{13}C NMR (101 MHz, DMSO-d_6) spectrum of **18**

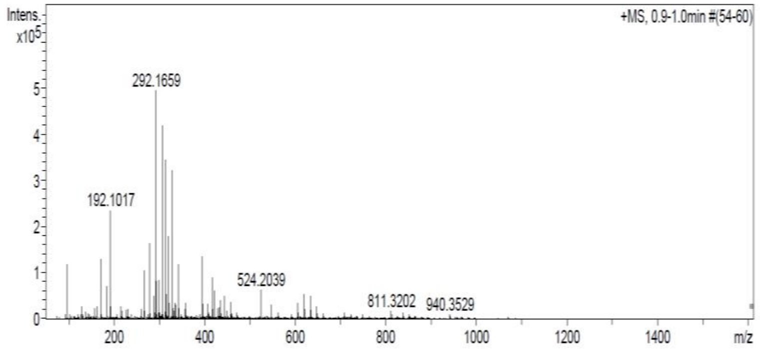

Meas. m/z	#	Formula	Score	m/z	err [ppm]	Mean err [ppm]	mSigma	rdb	e⁻ Conf	N-Rule
524.2039	1	C 28 H 26 N 7 O 4	100.00	524.2041	0.4	0.8	2.6	19.5	even	ok
	2	C 29 H 22 N 11	40.81	524.2054	2.9	3.3	11.8	24.5	even	ok
	3	C 27 H 30 N 3 O 8	49.77	524.2027	-2.2	-1.8	14.2	14.5	even	ok
	4	C 27 H 35 Br N 5 O	0.00	524.2019	-3.7	-4.1	401.2	12.5	even	ok

Figure S21. HRMS spectrum of **18**

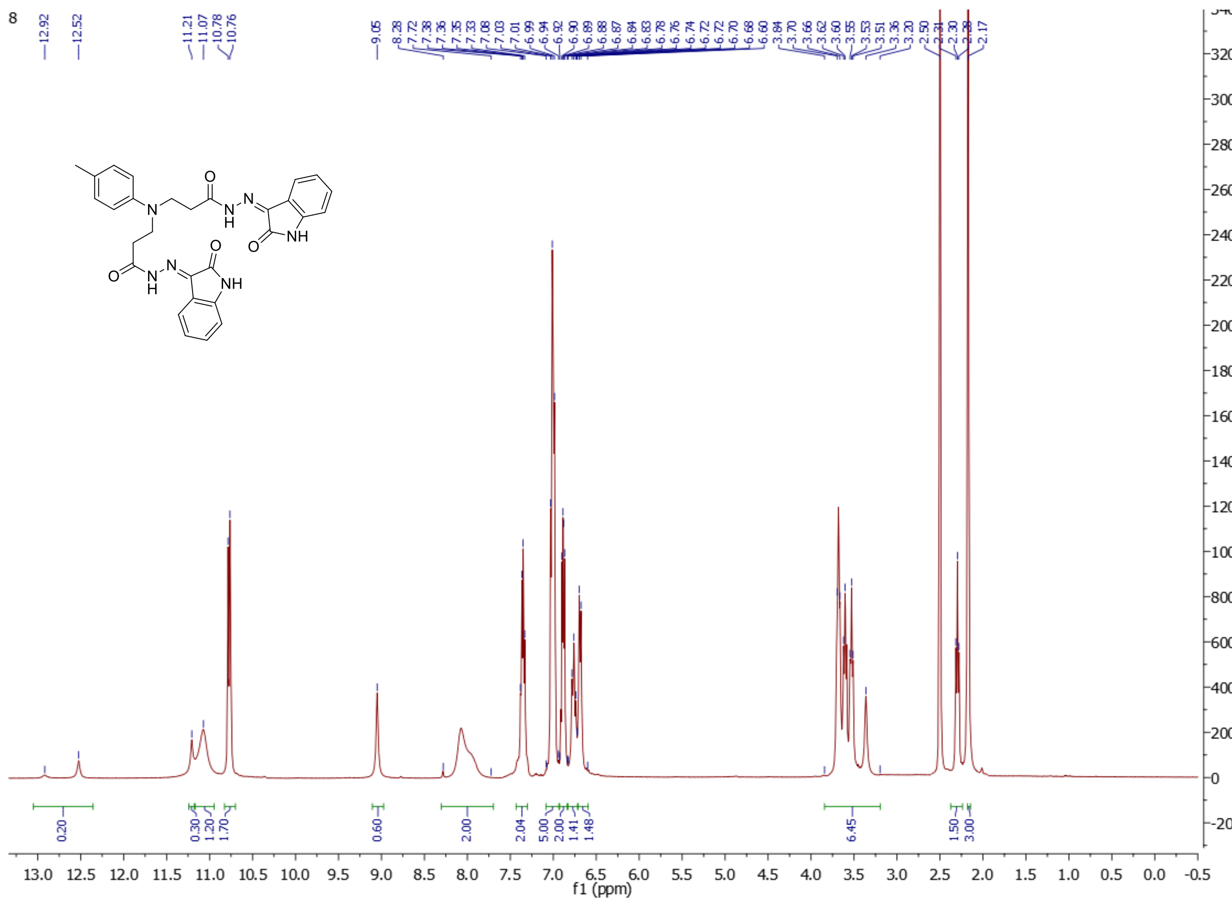

Figure S22. ^{1}H NMR (400 MHz, DMSO-*d*6) spectrum of **19**

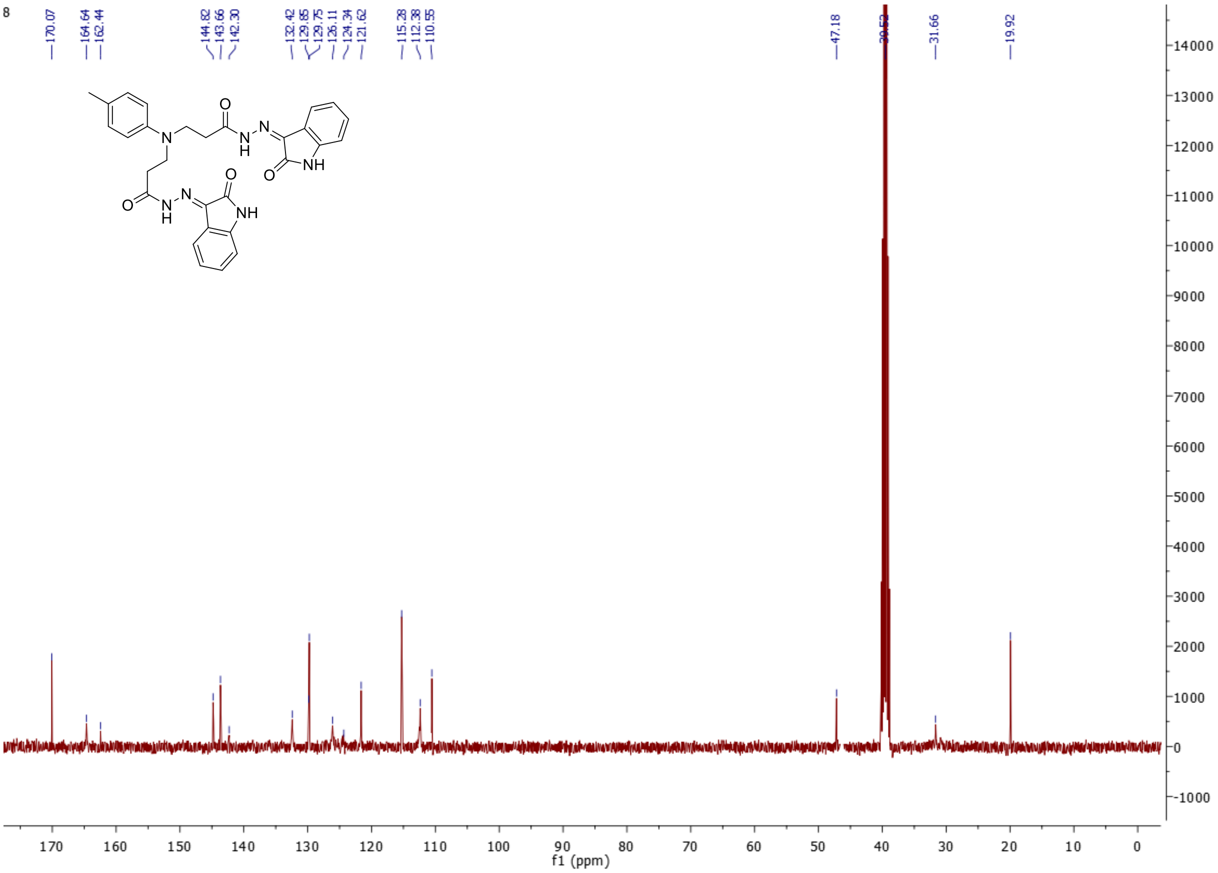

Figure S23. ^{13}C NMR (101 MHz, DMSO-d_6) spectrum of **19**

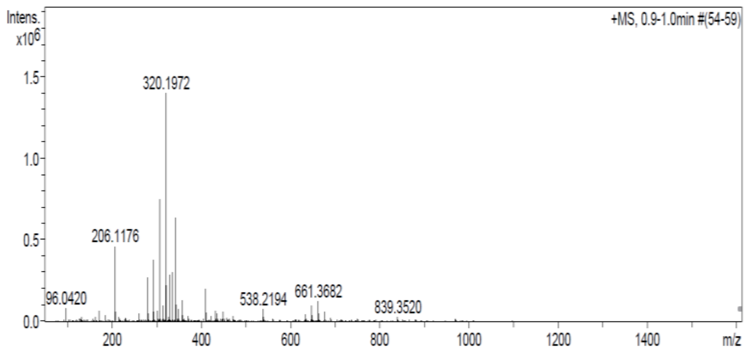

Meas. m/z	#	Formula	Score	m/z	err [ppm]	Mean err [ppm]	mSigma	rdb	e⁻ Conf	N-Rule
538.2194	1	C 29 H 28 N 7 O 4	100.00	538.2197	0.6	1.0	5.7	19.5	even	ok
	2	C 30 H 24 N 11	43.32	538.2211	3.1	3.4	9.4	24.5	even	ok
	3	C 28 H 32 N 3 O 8	60.07	538.2184	-1.8	-1.5	15.5	14.5	even	ok
	4	C 28 H 37 Br N 5 O	0.00	538.2176	-3.3	-3.7	400.9	12.5	even	ok

Figure S24. HRMS spectrum of **19**

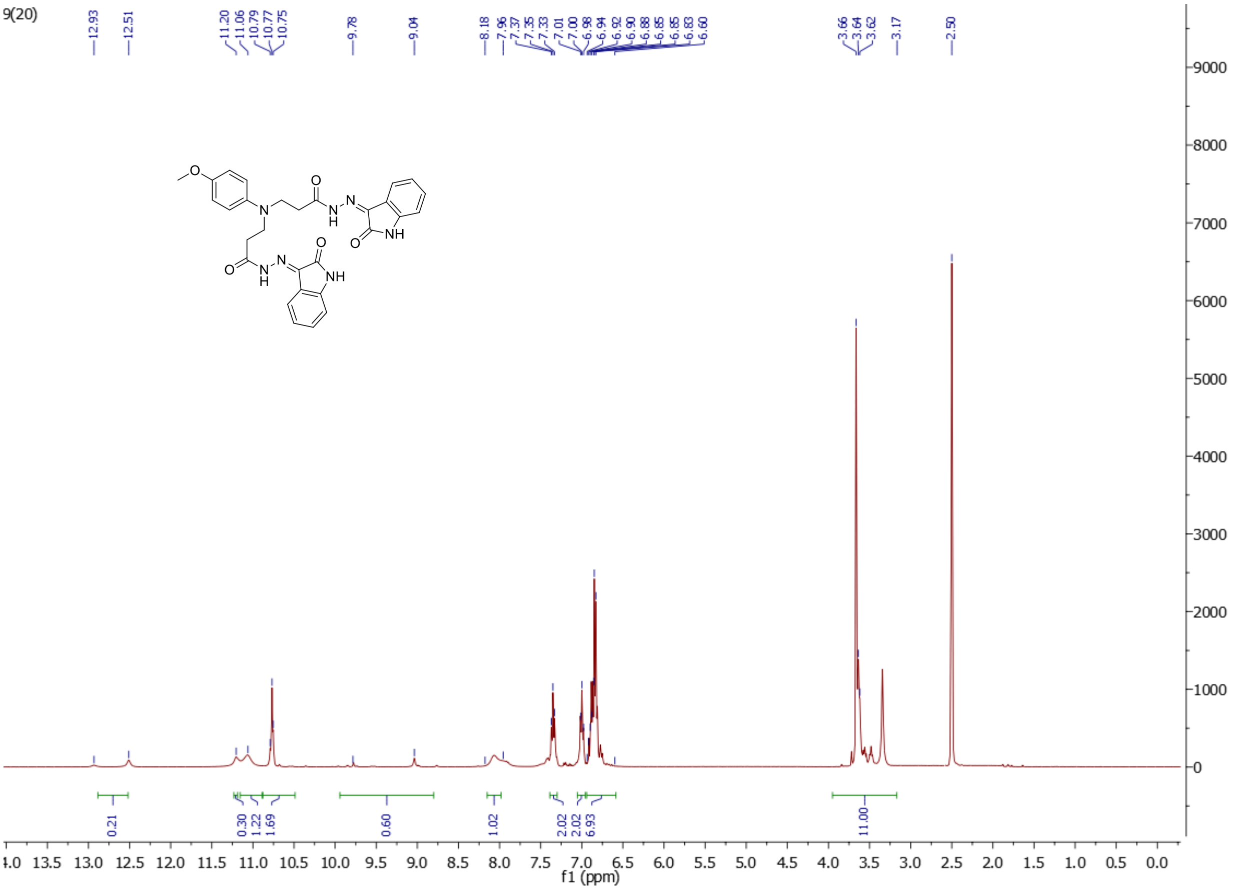

Figure S25. ^{1}H NMR (400 MHz, DMSO-d_6) spectrum of **20**

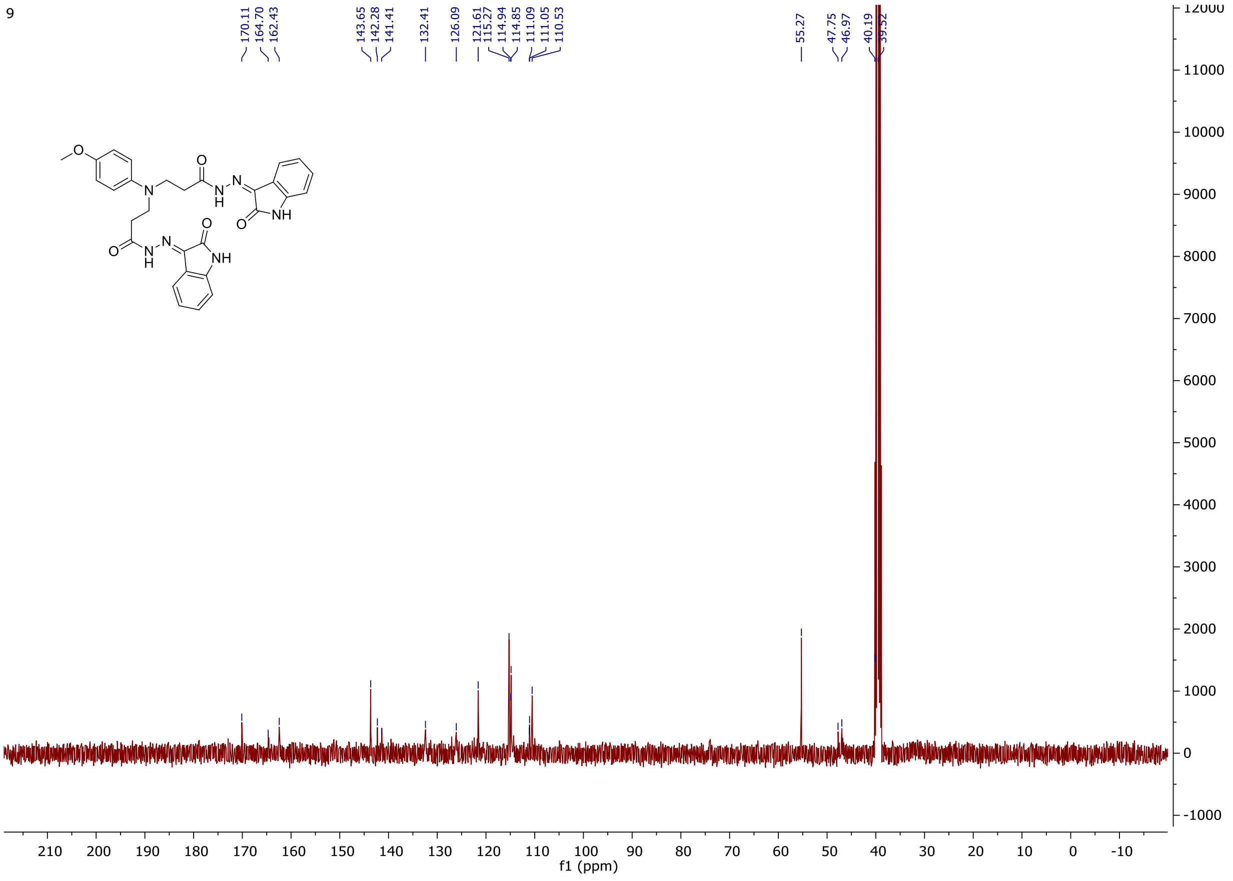

Figure S26. ^{13}C NMR (101 MHz, DMSO-d_6) spectrum of **20**

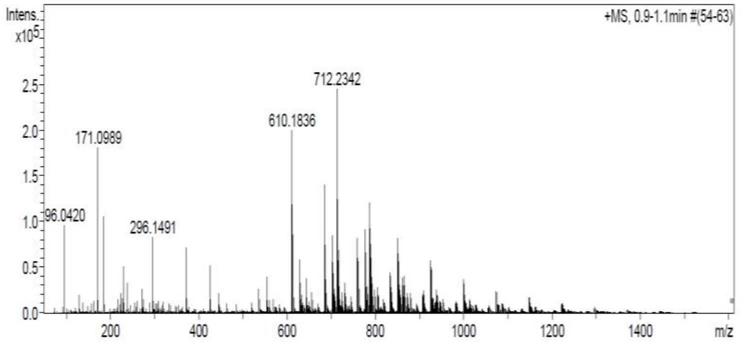

Meas. m/z	#	Formula	Score	m/z	err [ppm]	Mean err [ppm]	mSigma	rdb	e⁻ Conf	N-Rule
554.2137	1	C 29 H 28 N 7 O 5	92.76	554.2146	1.6	2.0	9.6	19.5	even	ok
	2	C 28 H 32 N 3 O 9	100.00	554.2133	-0.8	-0.4	17.8	14.5	even	ok
	3	C 25 H 24 N 13 O 3	39.49	554.2120	-3.2	-3.0	23.8	20.5	even	ok
	4	C 28 H 37 Br N 5 O 2	0.00	554.2125	-2.2	-2.8	395.6	12.5	even	ok

Figure S27. HRMS spectrum of **20**

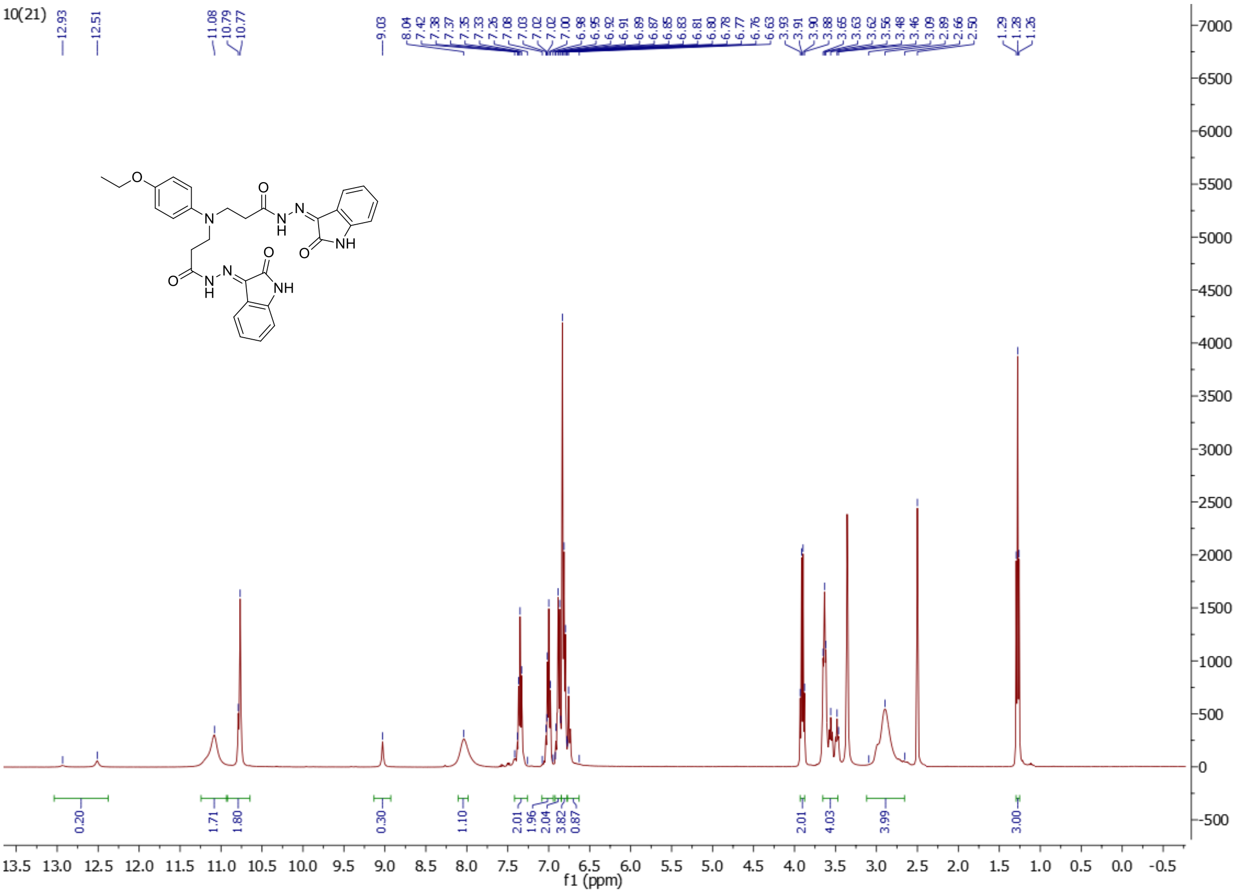

Figure S28. ^{1}H NMR (400 MHz, DMSO-d_6) spectrum of **21**

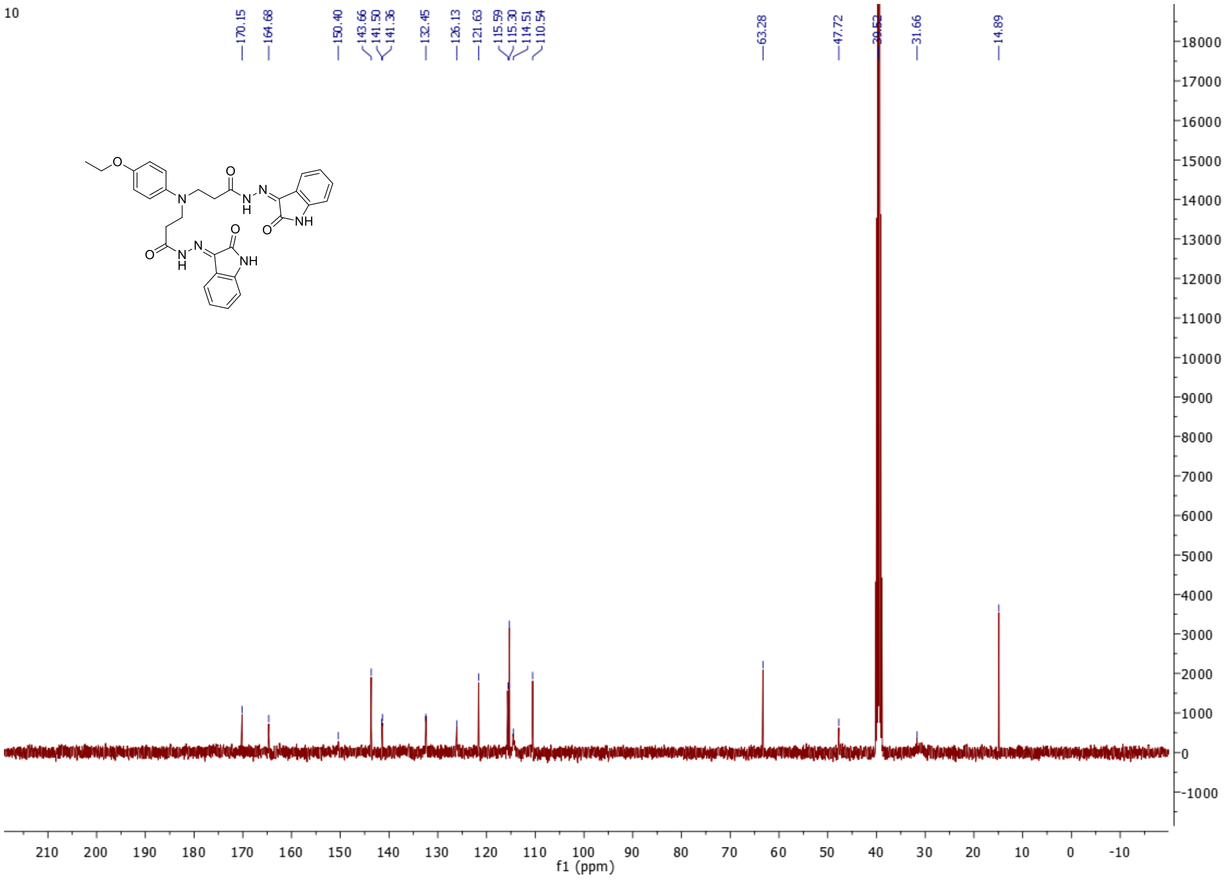

Figure S29. ^{13}C NMR (101 MHz, DMSO-d_6) spectrum of **21**

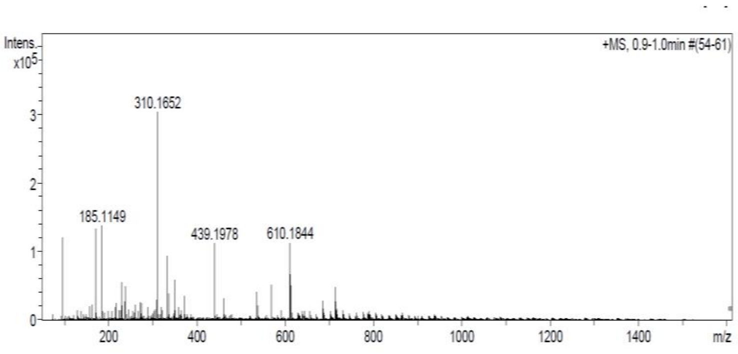

Meas. m/z	#	Formula	Score	m/z	err [ppm]	Mean err [ppm]	mSigma	rdb	e¯ Conf	N-Rule
310.1652	1	C 16 H 24 N O 5	100.00	310.1649	-1.0	-0.8	7.1	5.5	even	ok
	2	C 17 H 20 N 5 O	51.98	310.1662	3.3	3.4	22.0	10.5	even	ok
568.2302	1	C 30 H 30 N 7 O 5	100.00	568.2303	0.2	0.6	6.7	19.5	even	ok
	2	C 29 H 34 N 3 O 9	51.43	568.2290	-2.1	-1.8	13.3	14.5	even	ok
	3	C 31 H 26 N 11 O	42.53	568.2316	2.6	2.9	13.9	24.5	even	ok
610.1844	1	C 38 H 28 N O 7	68.55	610.1860	2.7	7.4	192.5	25.5	even	ok
	2	C 35 H 20 N 11 O	100.00	610.1847	0.5	5.1	197.3	31.5	even	ok
	[illegible]	[illegible]	[illegible]	[illegible]	[illegible]	[illegible]	[illegible]	[illegible]	[illegible]	[illegible]

Figure S30. HRMS spectrum of **21**

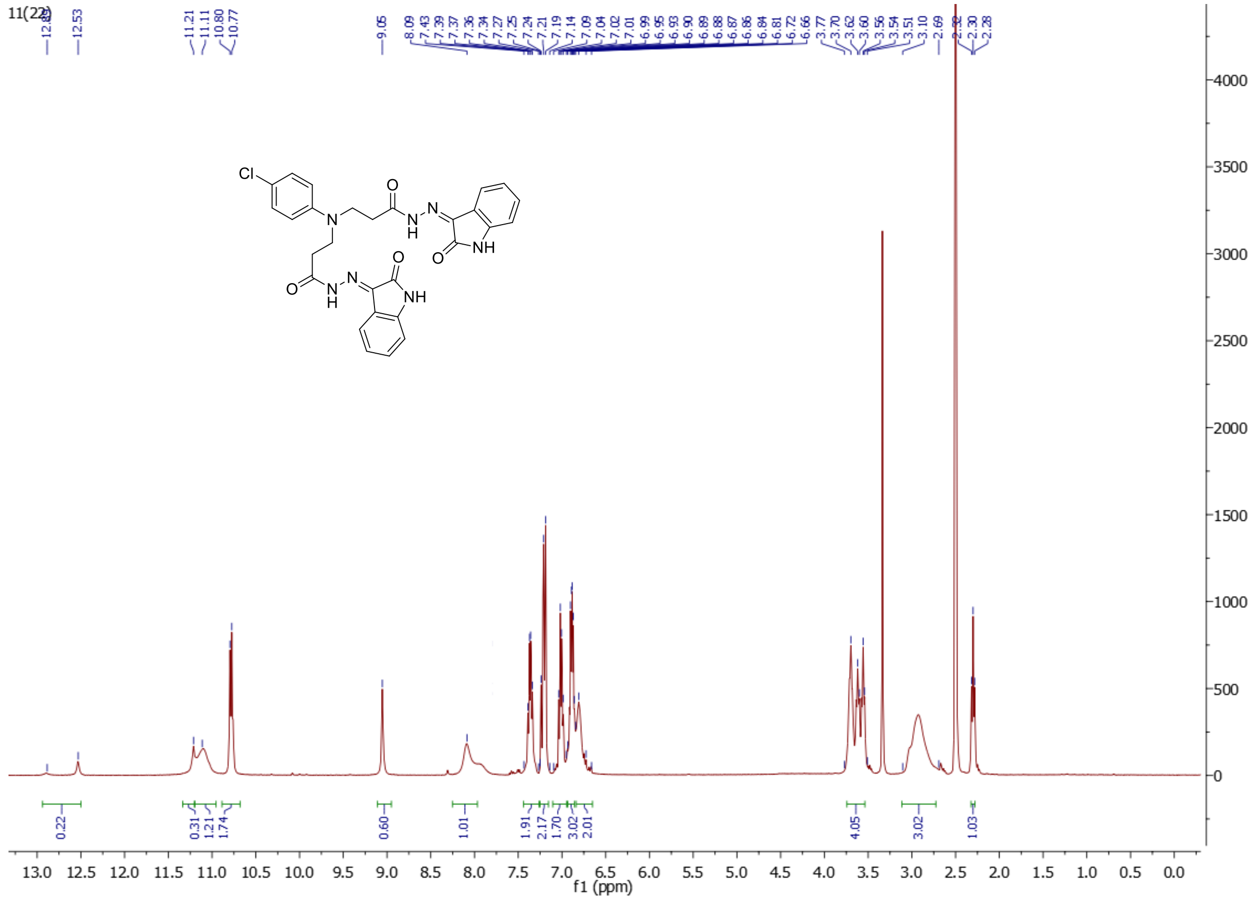

Figure S31. ^{1}H NMR (400 MHz, DMSO-d_6) spectrum of **22**

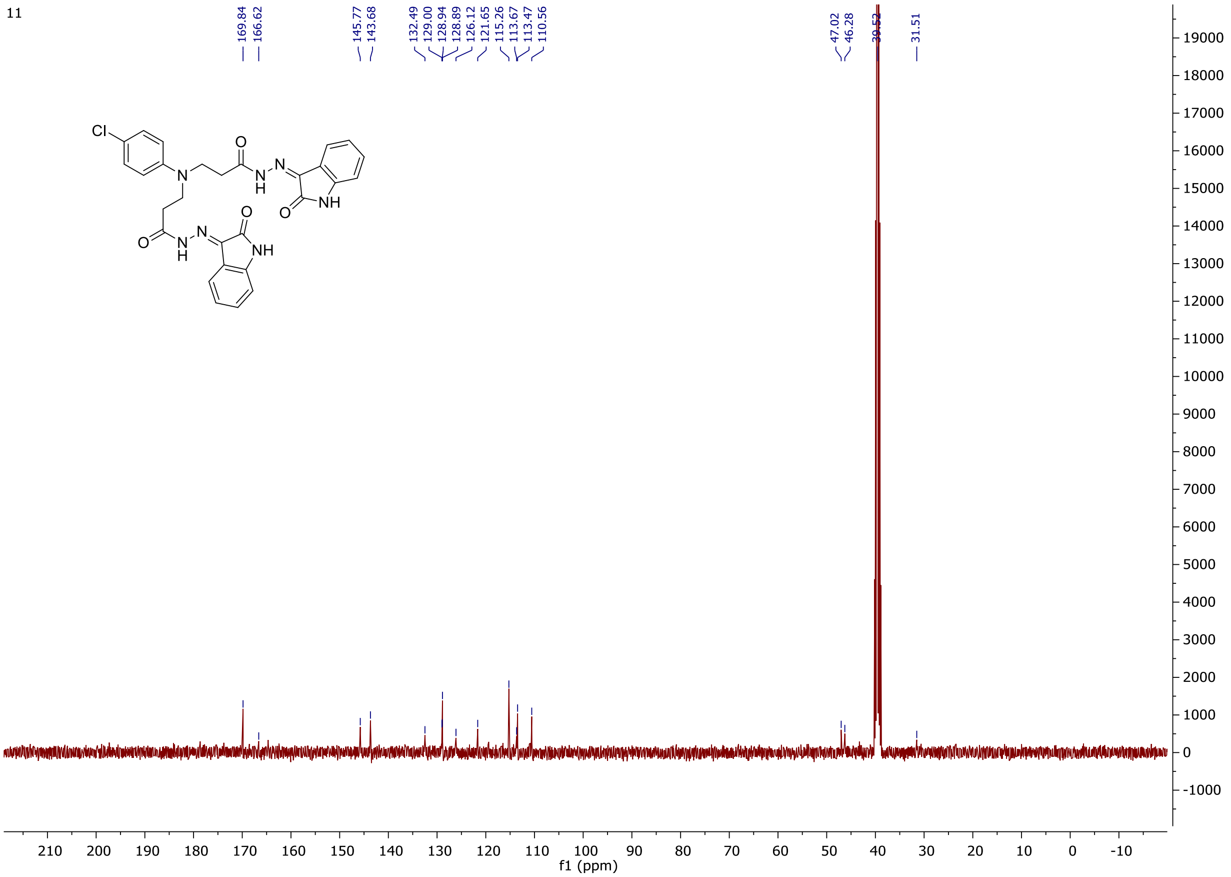

Figure S32. ^{13}C NMR (101 MHz, DMSO-d_6) spectrum of **22**

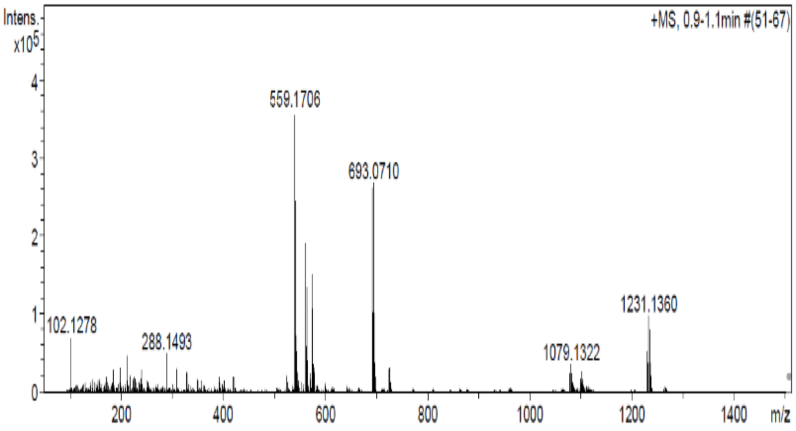

Meas. m/z	#	Formula	Score	m/z	err [ppm]	Mean err [ppm]	mSigma	rdb	e⁻ Conf	N-Rule
559.1706	1	C 28 H 24 Cl N 7 O 4	100.00	559.1706	0.0	0.1	24.7	17.5	even	ok

Figure S33. HRMS spectrum of **22**

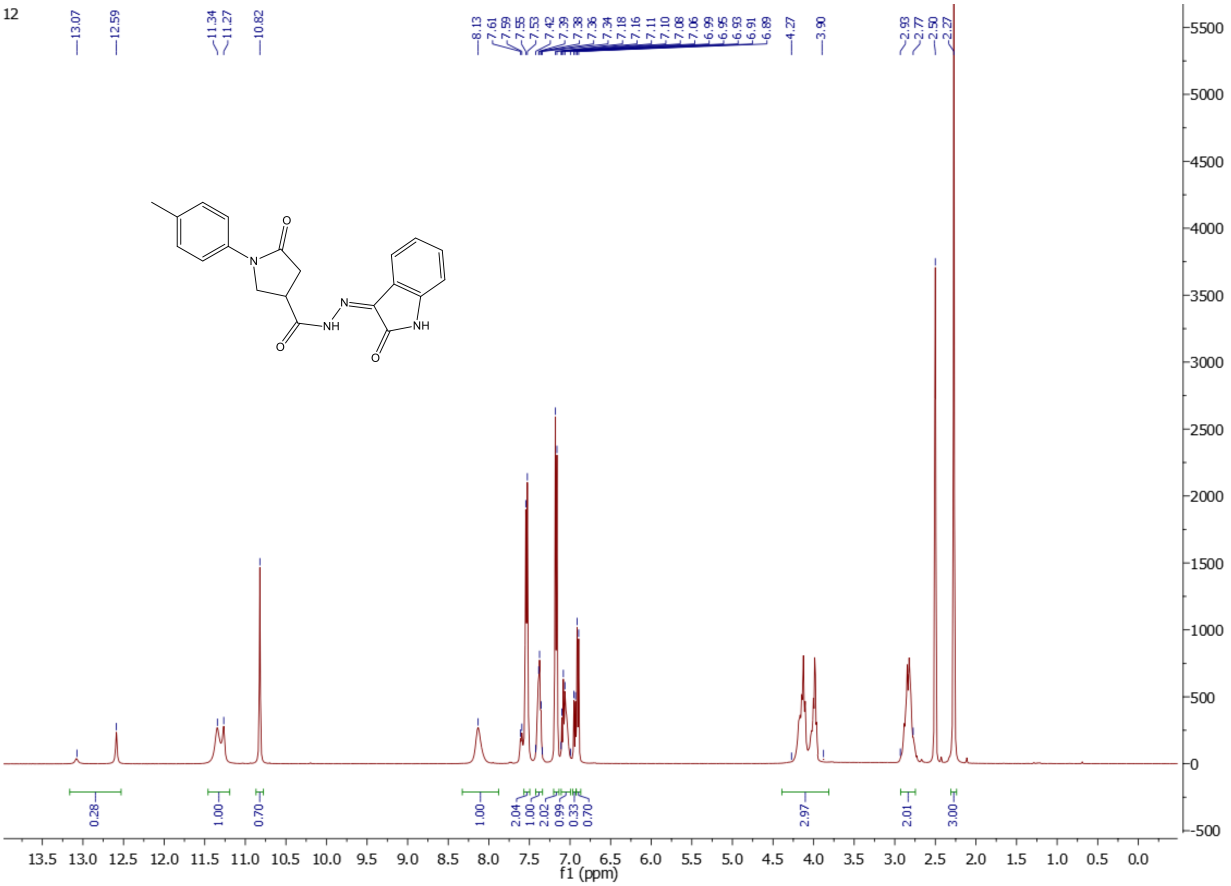

Figure S34. ^{1}H NMR (400 MHz, DMSO-d_6) spectrum of **26**

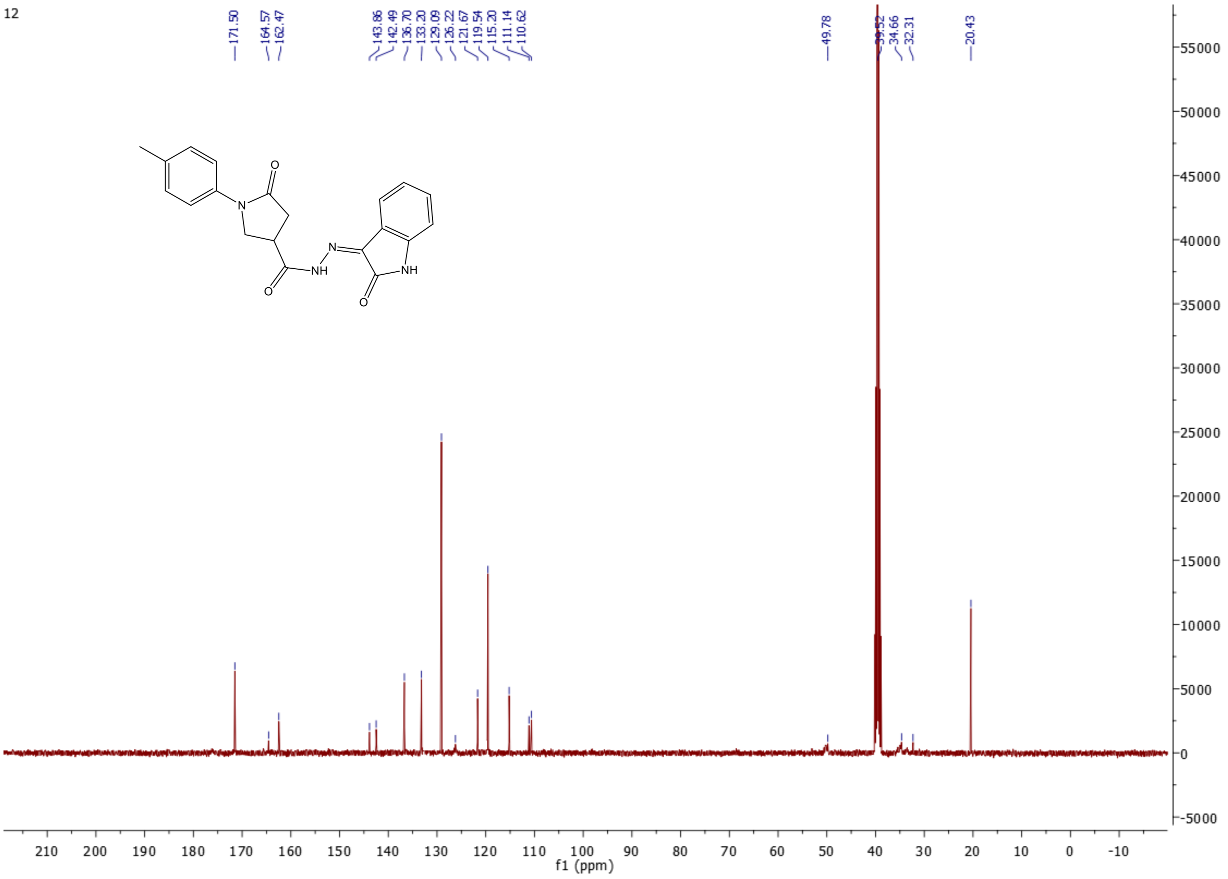

Figure S35. ^{13}C NMR (101 MHz, DMSO-d_6) spectrum of **26**

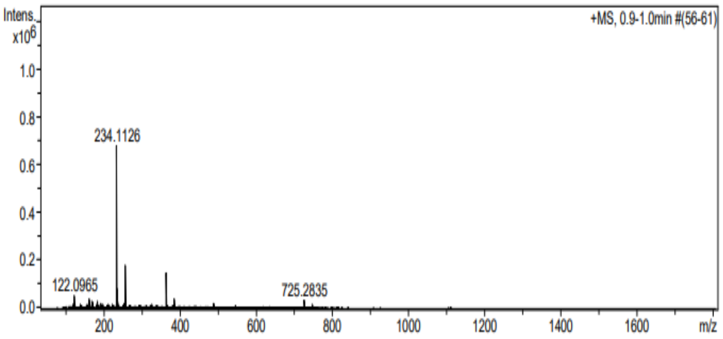

Meas. m/z	#	Formula	Score	m/z	err [ppm]	Mean err [ppm]	mSigma	rdb	e⁻ Conf	N-Rule
234.1126	1	C 13 H 16 N O 3	100.00	234.1125	-0.7	-0.5	15.1	6.5	even	ok
256.0944	1	C 11 H 10 N 7 O	100.00	256.0941	-0.9	-0.8	10.9	10.5	even	ok
	2	C 10 H 14 N 3 O 5	36.99	256.0928	-6.1	-6.1	23.2	5.5	even	ok
363.1453	1	C 20 H 19 N 4 O 3	100.00	363.1452	-0.4	-0.4	16.7	13.5	even	ok
	2	C 19 H 23 O 7	45.97	363.1438	-4.1	-4.1	19.3	8.5	even	ok

Figure S36. HRMS spectrum of **26**

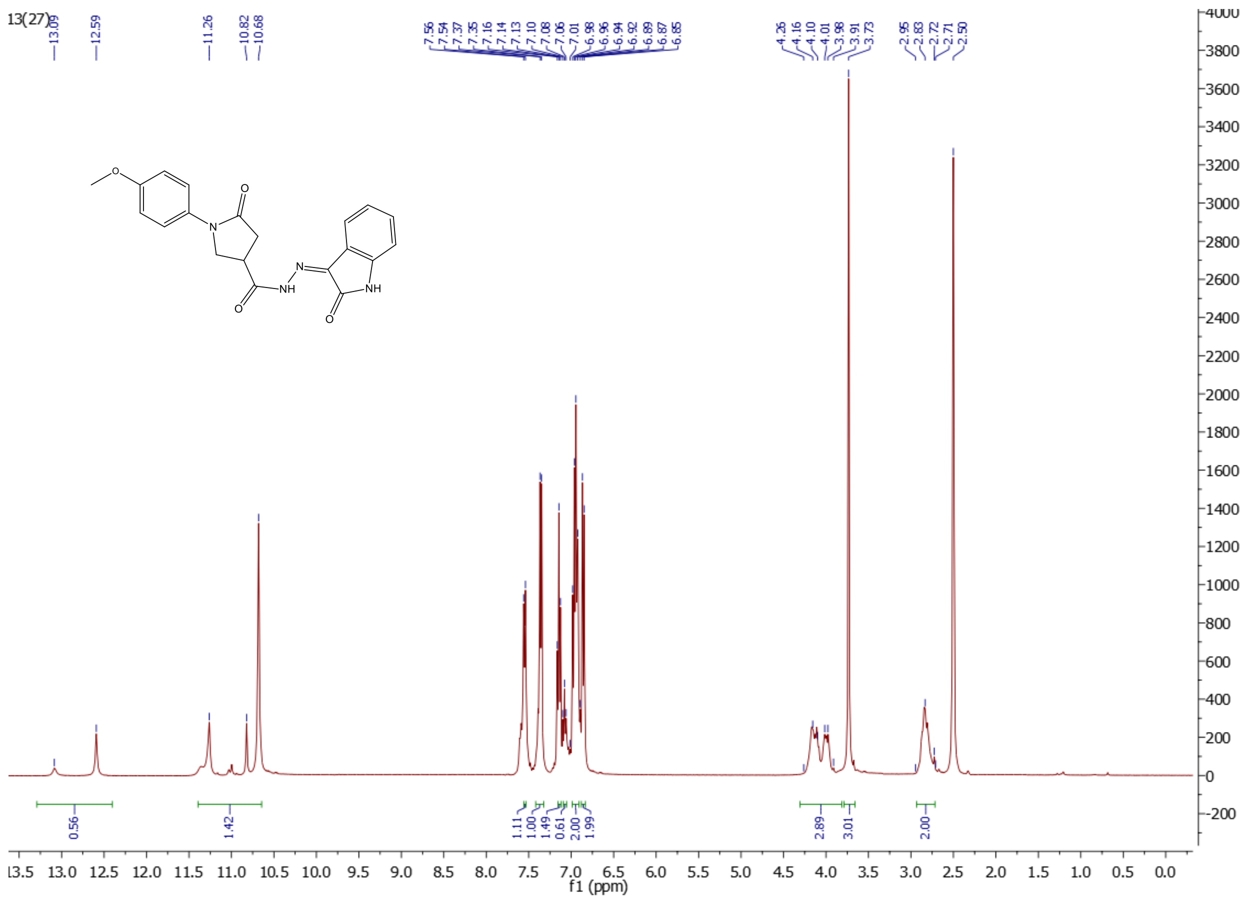

Figure S37. ^{1}H NMR (400 MHz, DMSO-d_6) spectrum of **27**

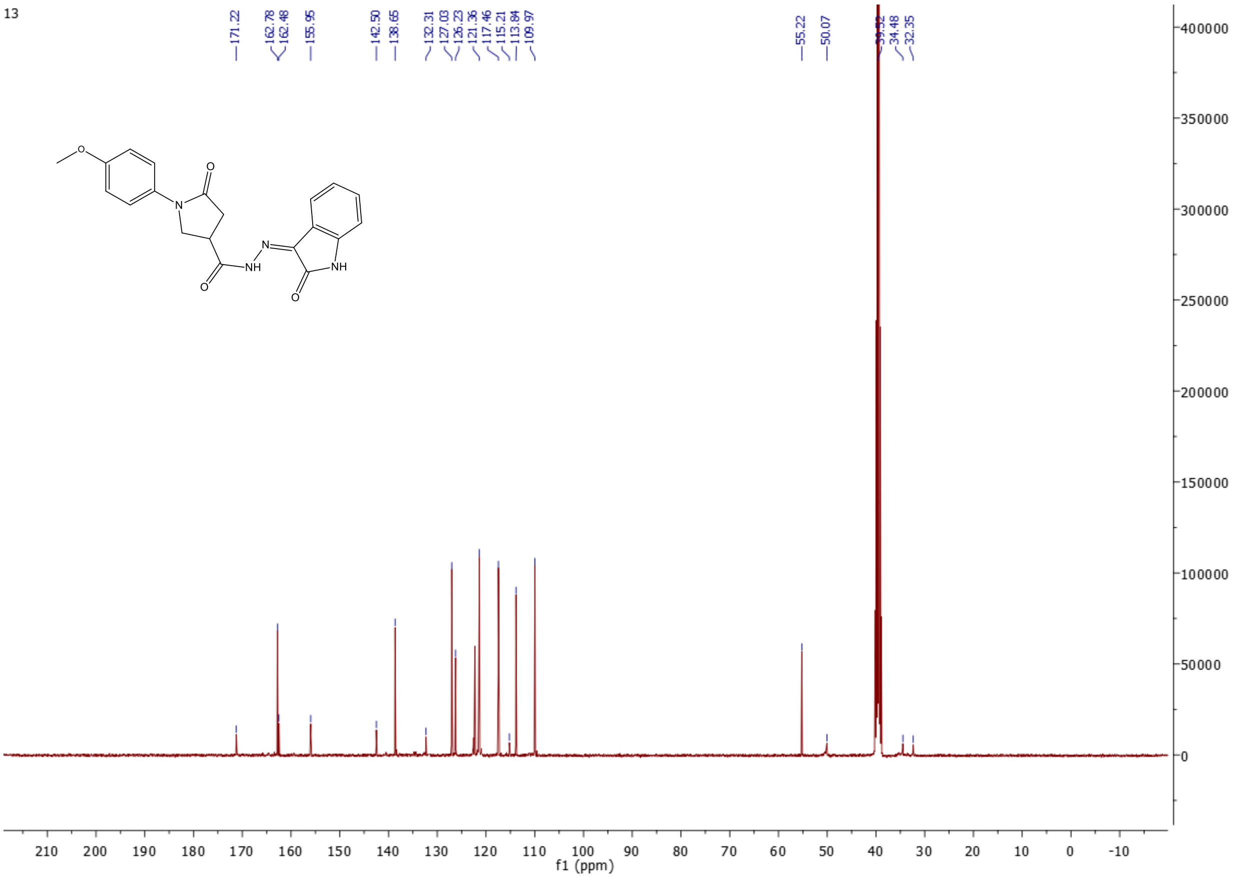

Figure S38. ^{13}C NMR (101 MHz, DMSO-d_6) spectrum of **27**

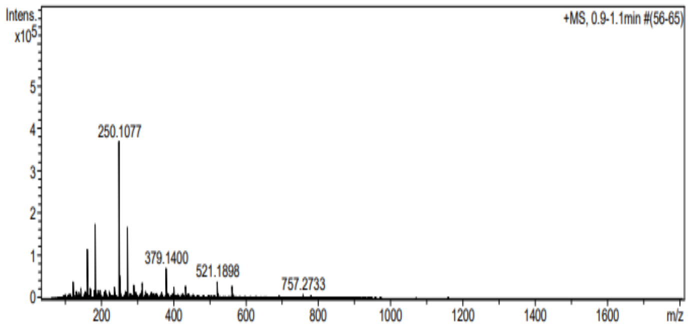

Meas. m/z	#	Formula	Score	m/z	err [ppm]	Mean err [ppm]	mSigma	rdb	e⁻ Conf	N-Rule
184.0481	1	C 5 H 6 N 5 O 3	100.00	184.0465	-8.7	-8.3	12.3	5.5	even	ok
250.1077	1	C 13 H 16 N O 4	100.00	250.1074	-1.1	-1.1	1.8	6.5	even	ok
	2	C 14 H 12 N 5	53.84	250.1087	4.3	4.2	13.7	11.5	even	ok
272.0895	1	C 11 H 10 N 7 O 2	100.00	272.0890	-1.7	-1.5	6.8	10.5	even	ok
	2	C 10 H 14 N 3 O 6	34.88	272.0877	-6.6	-6.6	19.9	5.5	even	ok
379.1400	1	C 20 H 19 N 4 O 4	100.00	379.1401	0.1	-0.6	44.6	13.5	even	ok
	2	C 19 H 23 O 8	50.88	379.1387	-3.4	-4.1	45.8	8.5	even	ok
	3	C 21 H 15 N 8	45.17	379.1414	3.6	2.9	48.0	18.5	even	ok

Figure S39. HRMS spectrum of **27**

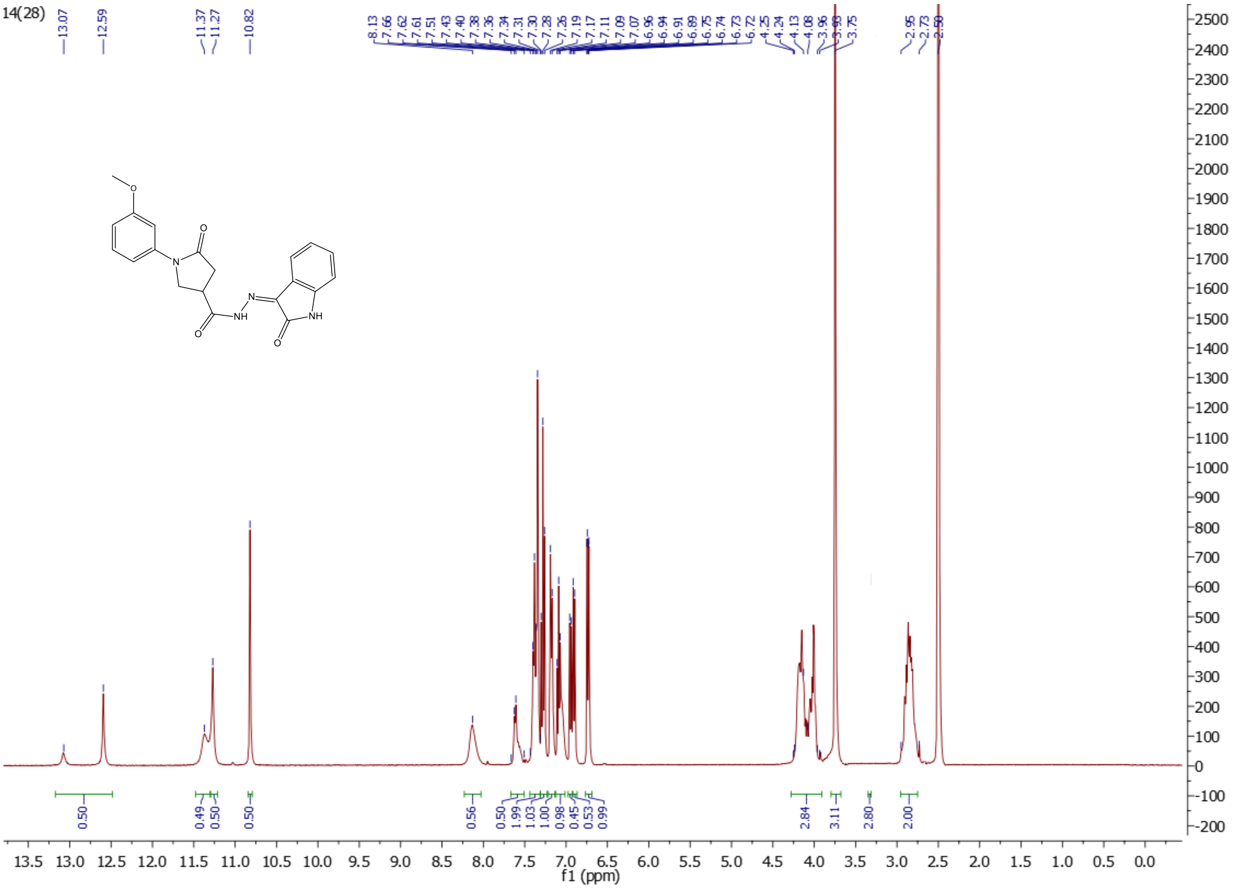

Figure S40. ^{1}H NMR (400 MHz, DMSO-d_6) spectrum of **28**

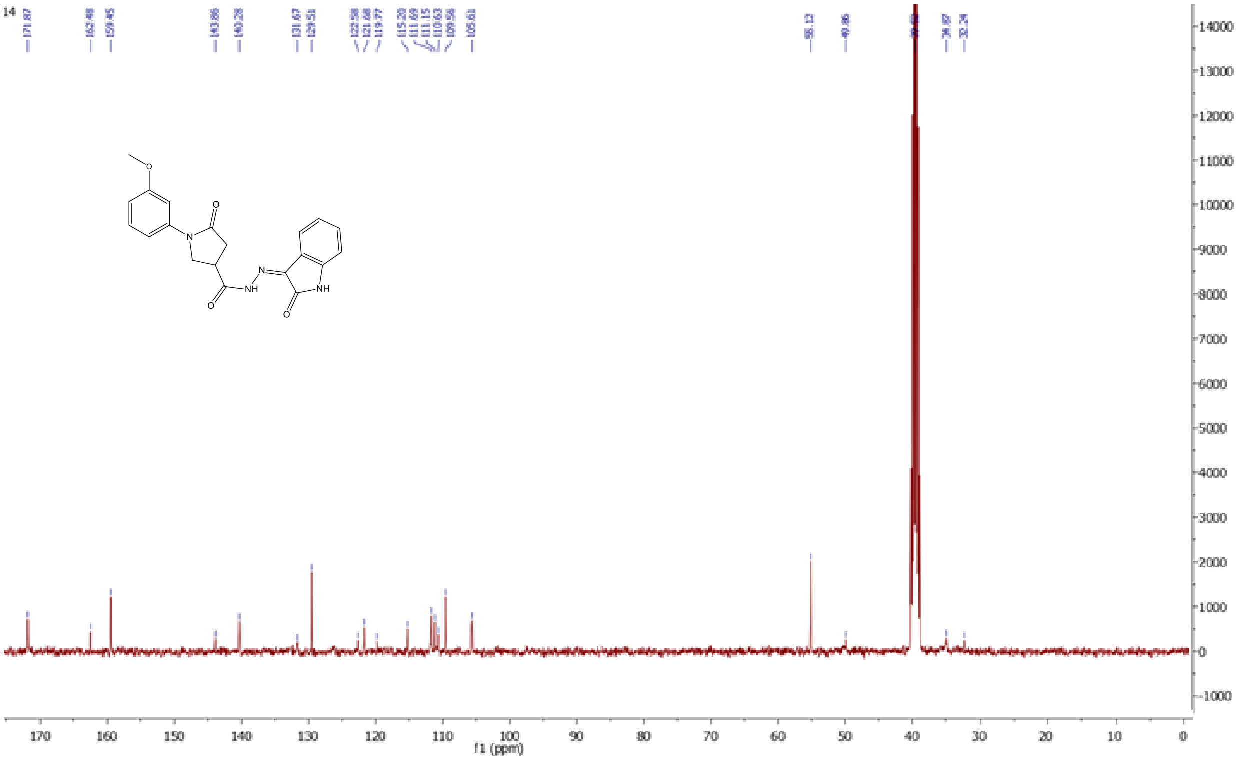

Figure S41. ^{13}C NMR (101 MHz, DMSO-d_6) spectrum of **28**

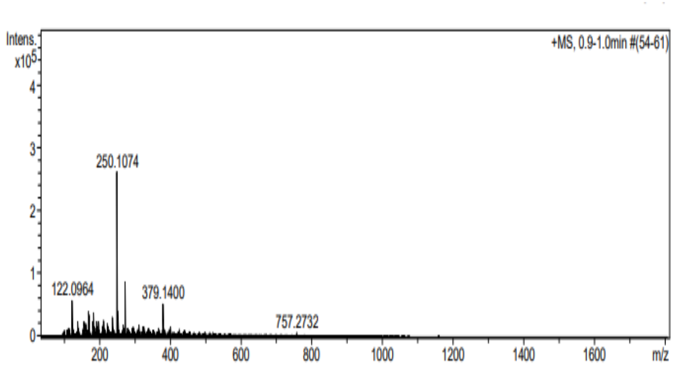

Meas. m/z	#	Formula	Score	m/z	err [ppm]	Mean err [ppm]	mSigma	rdb	e⁻ Conf	N-Rule
250.1074	1	C 13 H 16 N O 4	100.00	250.1074	-0.0	-0.3	7.0	6.5	even	ok
	2	C 14 H 12 N 5	48.76	250.1087	5.3	5.0	8.9	11.5	even	ok
272.0891	1	C 11 H 10 N 7 O 2	100.00	272.0890	-0.1	-0.3	13.4	10.5	even	ok
	2	C 10 H 14 N 3 O 6	36.61	272.0877	-5.0	-5.2	27.8	5.5	even	ok
379.1400	1	C 20 H 19 N 4 O 4	100.00	379.1401	0.3	-0.7	53.4	13.5	even	ok
	2	C 19 H 23 O 8	54.57	379.1387	-3.2	-4.2	55.1	8.5	even	ok
	3	C 21 H 15 N 8	45.69	379.1414	3.9	2.8	55.5	18.5	even	ok

Figure S42. HRMS spectrum of **28**

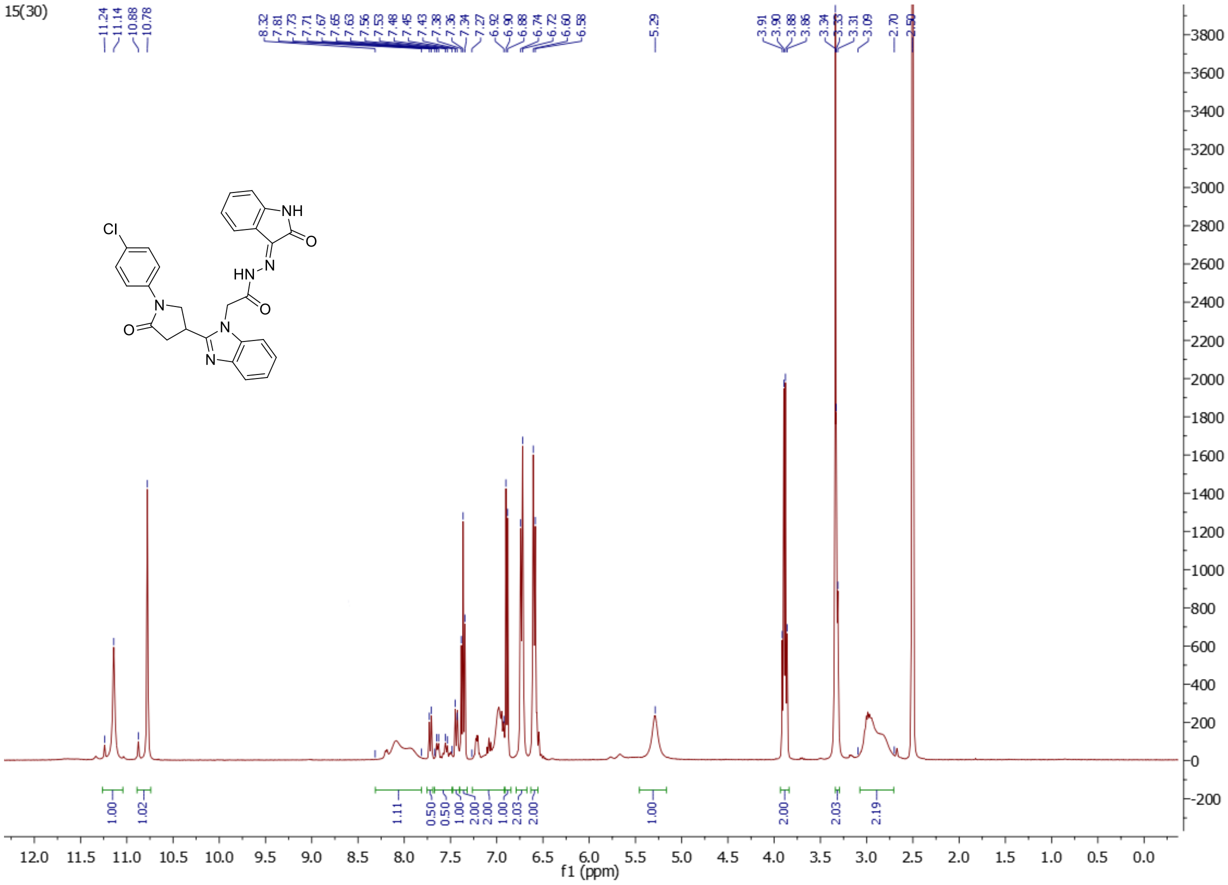

Figure S43. ^{1}H NMR (400 MHz, DMSO-*d*$_6$) spectrum of **30**

15

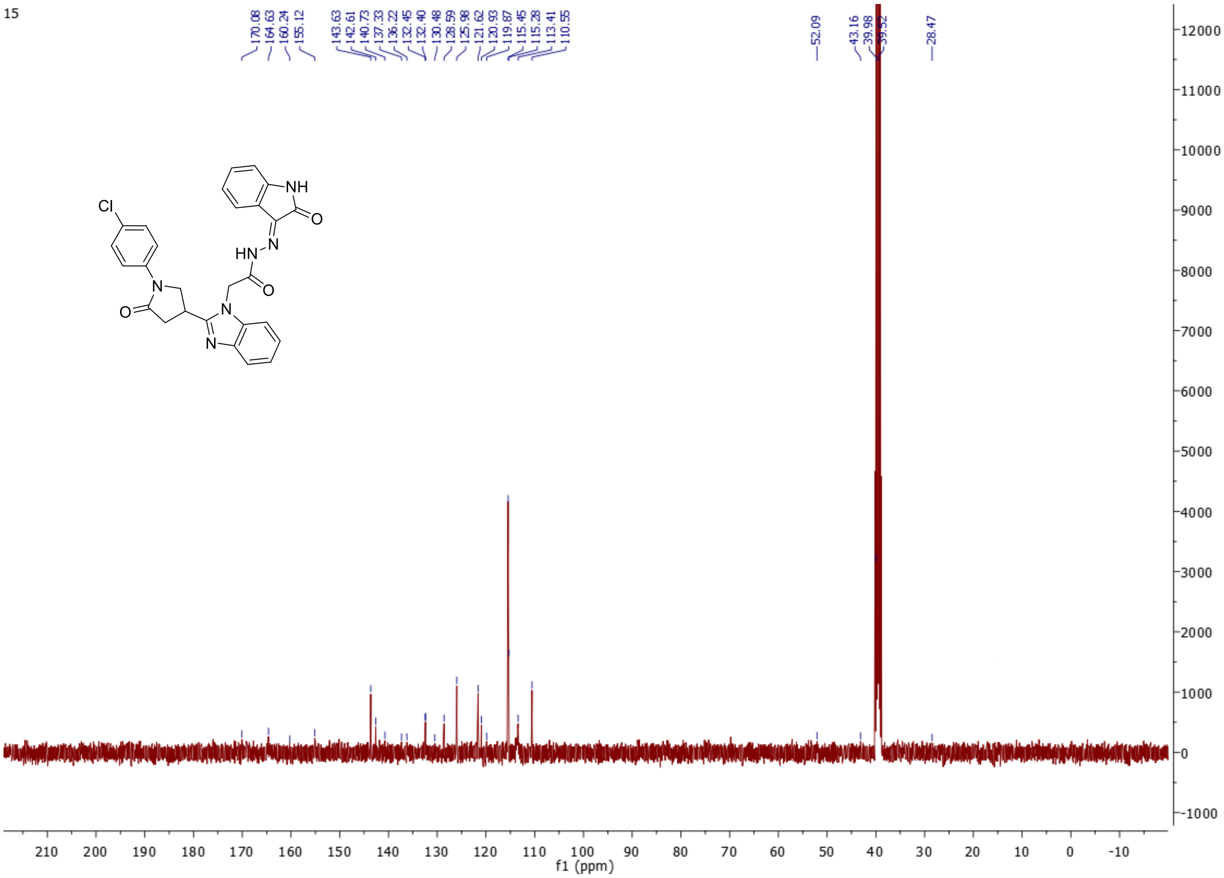

Figure S44. ^{13}C NMR (101 MHz, DMSO-d_6) spectrum of **30**

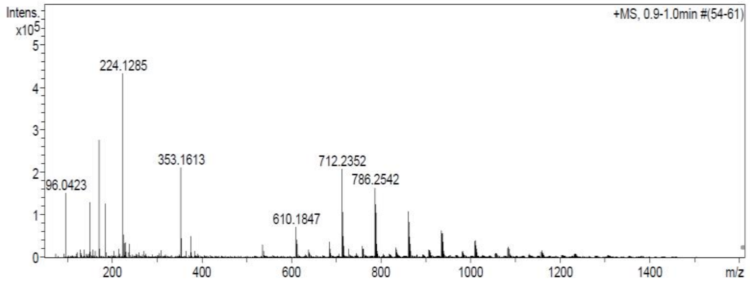

Meas. m/z	#	Formula	Score	m/z	err [ppm]	Mean err [ppm]	mSigma	rdb	e⁻ Conf	N-Rule
536.1656	1	C 39 H 22 N O 2	100.00	536.1645	-2.1	2.1	131.1	29.5	even	ok
	2	C 27 H 26 N 3 O 9	8.10	536.1664	1.4	5.5	172.3	16.5	even	ok
	3	C 23 H 22 N 9 O 7	1.67	536.1637	-3.6	0.5	182.8	17.5	even	ok
	4	C 24 H 18 N 13 O 3	0.74	536.1650	-1.2	2.9	203.0	22.5	even	ok
	5	C 10 H 30 N 7 O 18	0.03	536.1642	-2.7	0.6	234.6	-0.5	even	ok
	6	C 10 H 10 N 29	0.01	536.1668	2.3	4.1	249.0	20.5	even	ok
	7	C 12 H 22 N 15 O 10	0.00	536.1669	2.3	5.5	253.8	9.5	even	ok
	8	C 9 H 14 N 25 O 4	0.00	536.1655	-0.2	1.6	257.9	15.5	even	ok
	9	C 11 H 26 N 11 O 14	0.00	536.1655	-0.2	3.1	262.2	4.5	even	ok
	10	C 8 H 18 N 21 O 8	0.00	536.1642	-2.7	0.5	269.4	10.5	even	ok

Figure S45. HRMS spectrum of **30**

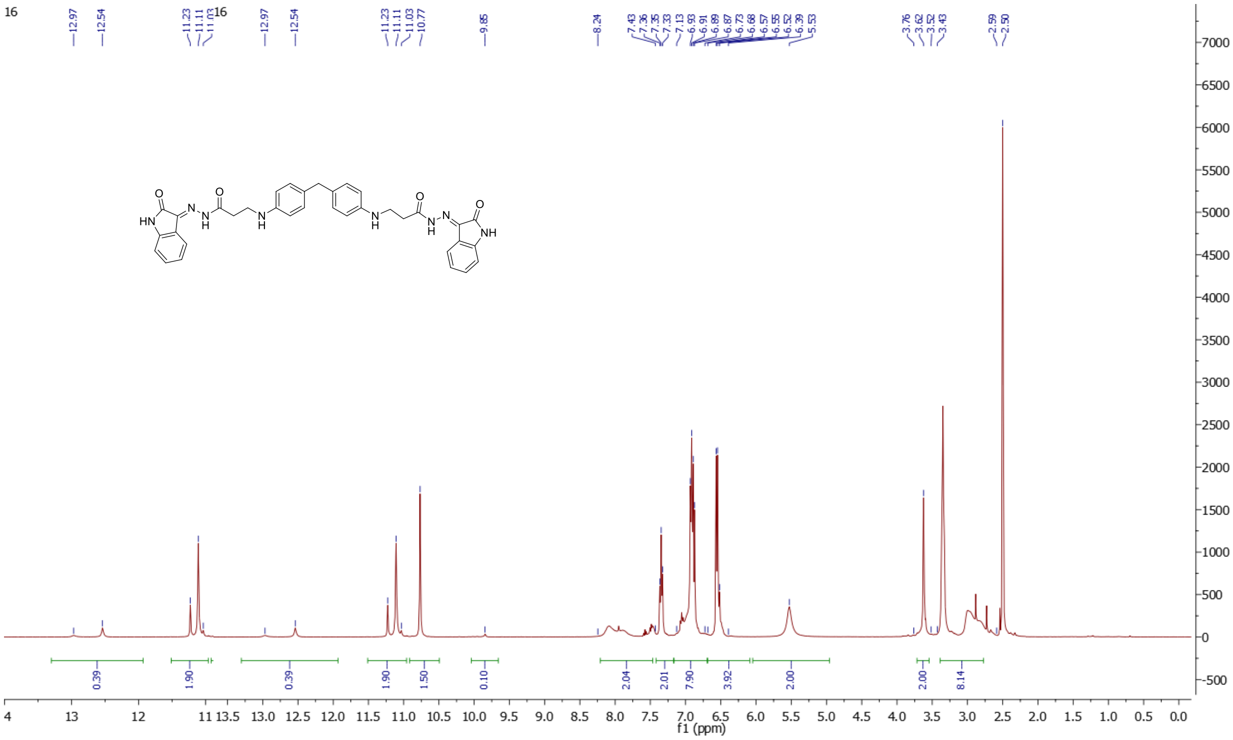

Figure S46. ^{1}H NMR (400 MHz, DMSO-*d*6) spectrum of **32**

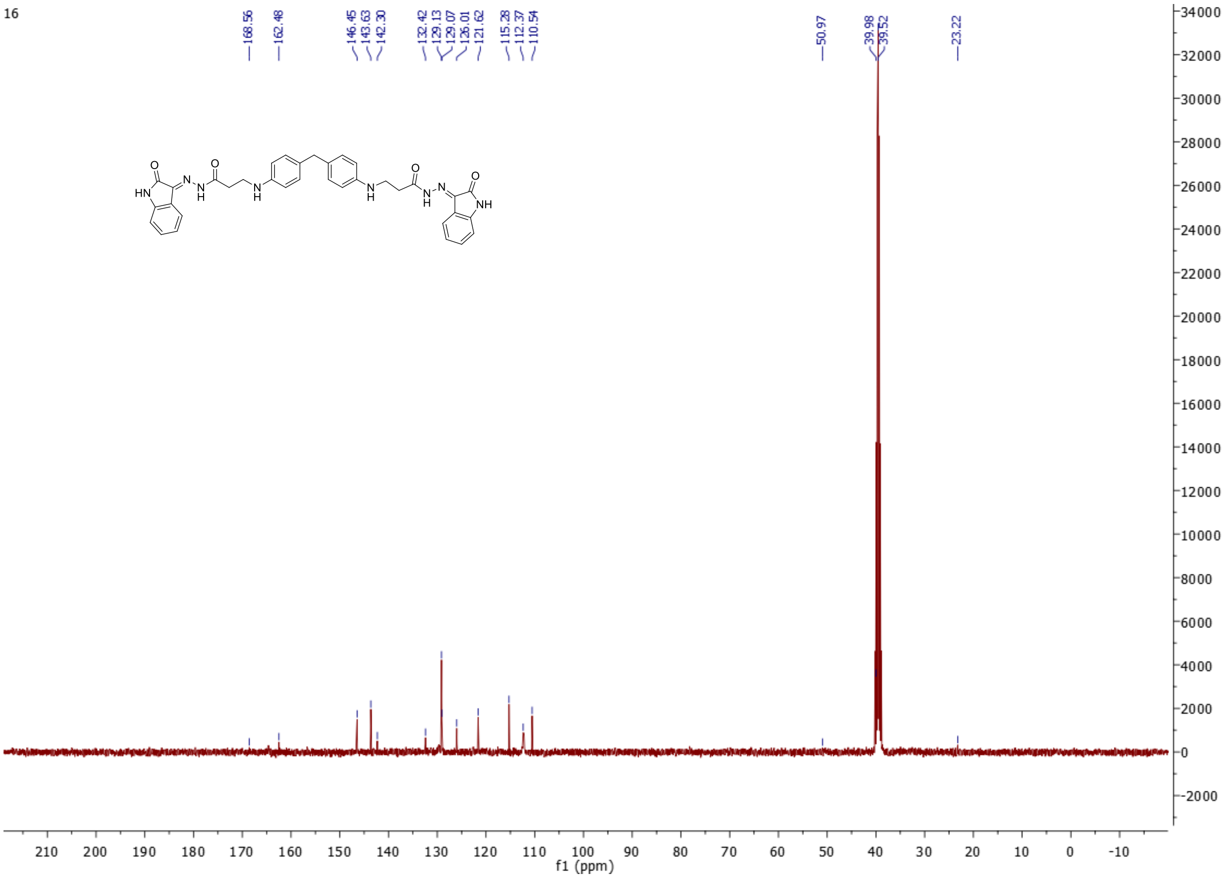

Figure S47. ^{13}C NMR (101 MHz, DMSO-d_6) spectrum of **32**

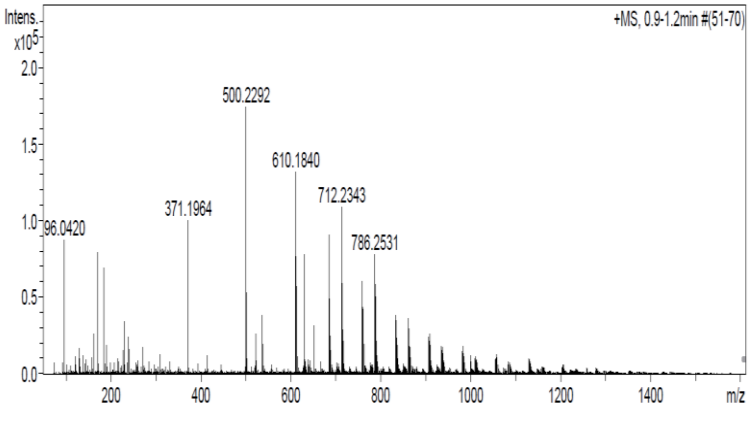

Meas. m/z	#	Formula	Score	m/z	err [ppm]	Mean err [ppm]	mSigma	rdb	e⁻ Conf	N-Rule
629.2617	1	C 35 H 33 N 8 O 4	100.00	629.2619	0.3	0.6	4.6	23.5	even	ok
	2	C 36 H 29 N 12	43.29	629.2633	2.5	2.7	10.3	28.5	even	ok
	3	C 34 H 37 N 4 O 8	51.55	629.2606	-1.8	-1.5	15.0	18.5	even	ok

Figure S48. HRMS spectrum of **32**

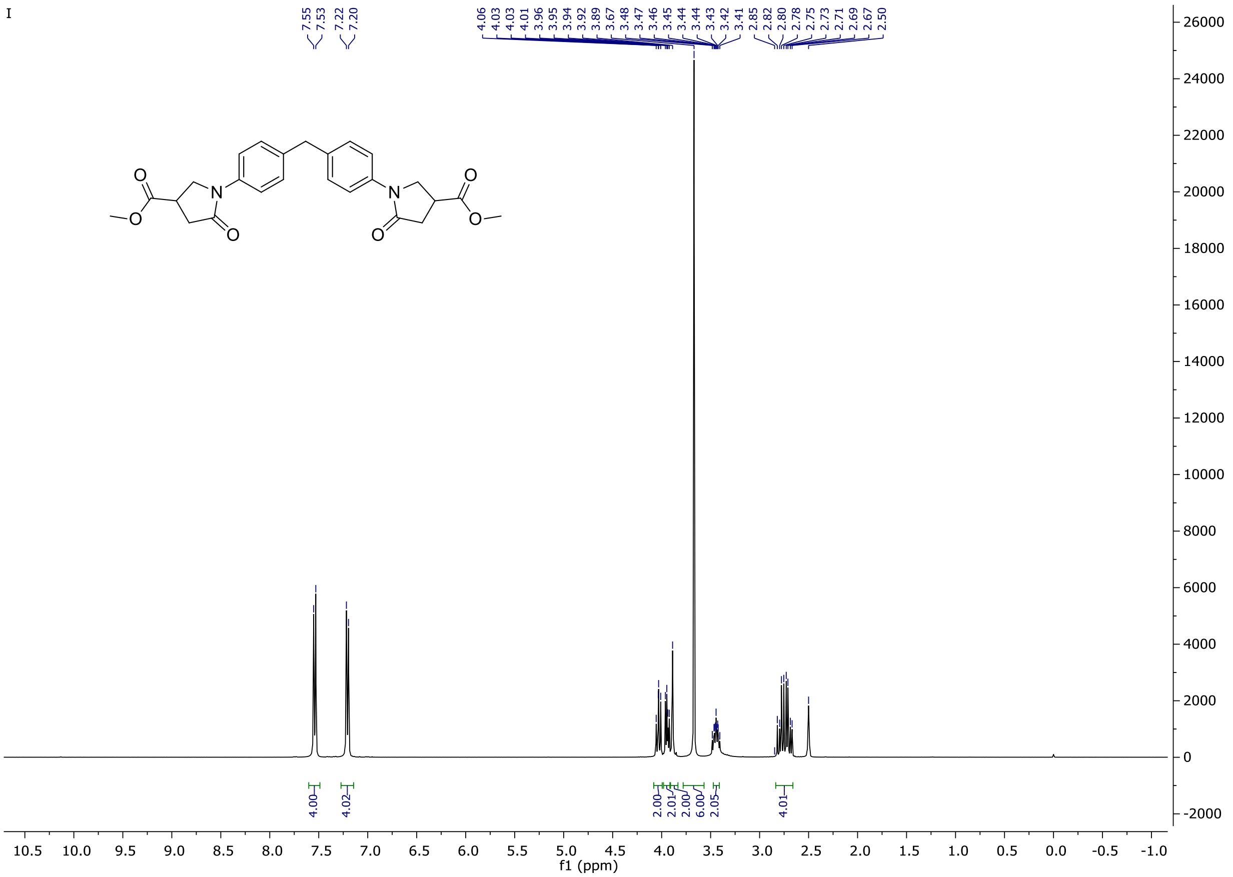

Figure S49. ^{1}H NMR (400 MHz, DMSO-d_6) spectrum of **33**

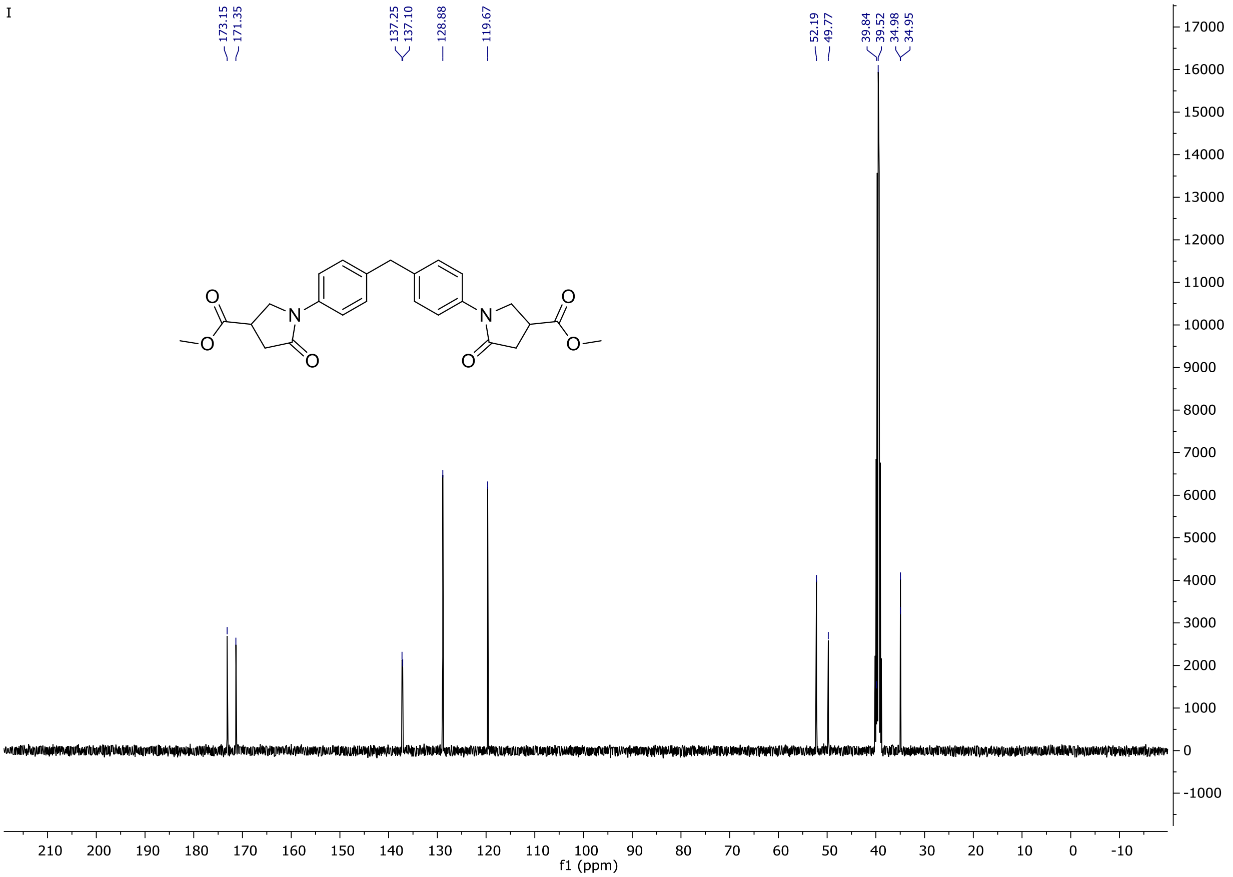

Figure S50. ^{13}C NMR (101 MHz, DMSO-d_6) spectrum of **33**

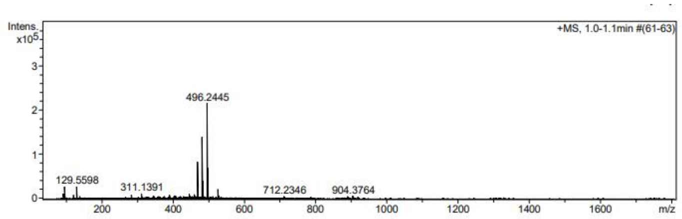

Meas. m/z	#	Formula	Score	m/z	err [ppm]	Mean err [ppm]	mSigma	rdb	e⁻ Conf	N-Rule
468.2130	1	C 26 H 26 N 7 O 2	54.31	468.2142	2.8	2.8	6.6	17.5	even	ok
	2	C 25 H 30 N 3 O 6	100.00	468.2129	-0.1	0.0	8.2	12.5	even	ok
	3	C 22 H 22 N 13	48.76	468.2116	-3.0	-2.9	9.1	18.5	even	ok
482.2288	1	C 26 H 32 N 3 O 6	100.00	482.2286	-0.5	-0.3	5.4	12.5	even	ok
	2	C 23 H 24 N 13	43.46	482.2272	-3.3	-3.2	10.1	18.5	even	ok
	3	C 27 H 28 N 7 O 2	52.41	482.2299	2.3	2.4	15.9	17.5	even	ok
496.2445	1	C 27 H 34 N 3 O 6	100.00	496.2442	-0.6	-0.3	6.4	12.5	even	ok
	2	C 28 H 30 N 7 O 2	67.91	496.2455	2.1	2.3	7.3	17.5	even	ok
	3	C 24 H 26 N 13	43.18	496.2429	-3.3	-3.1	10.4	18.5	even	ok

Figure S51. HRMS spectrum of **33**

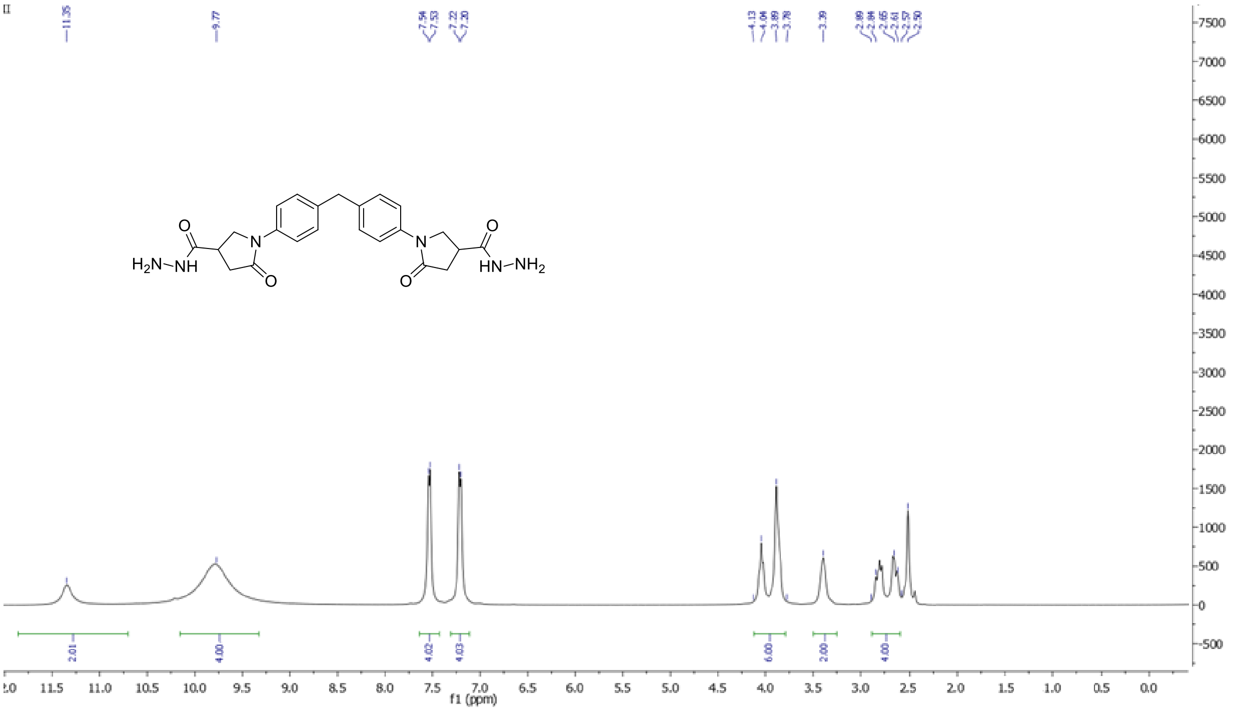

Figure S52. ^{1}H NMR (400 MHz, DMSO-d_6) spectrum of **34**

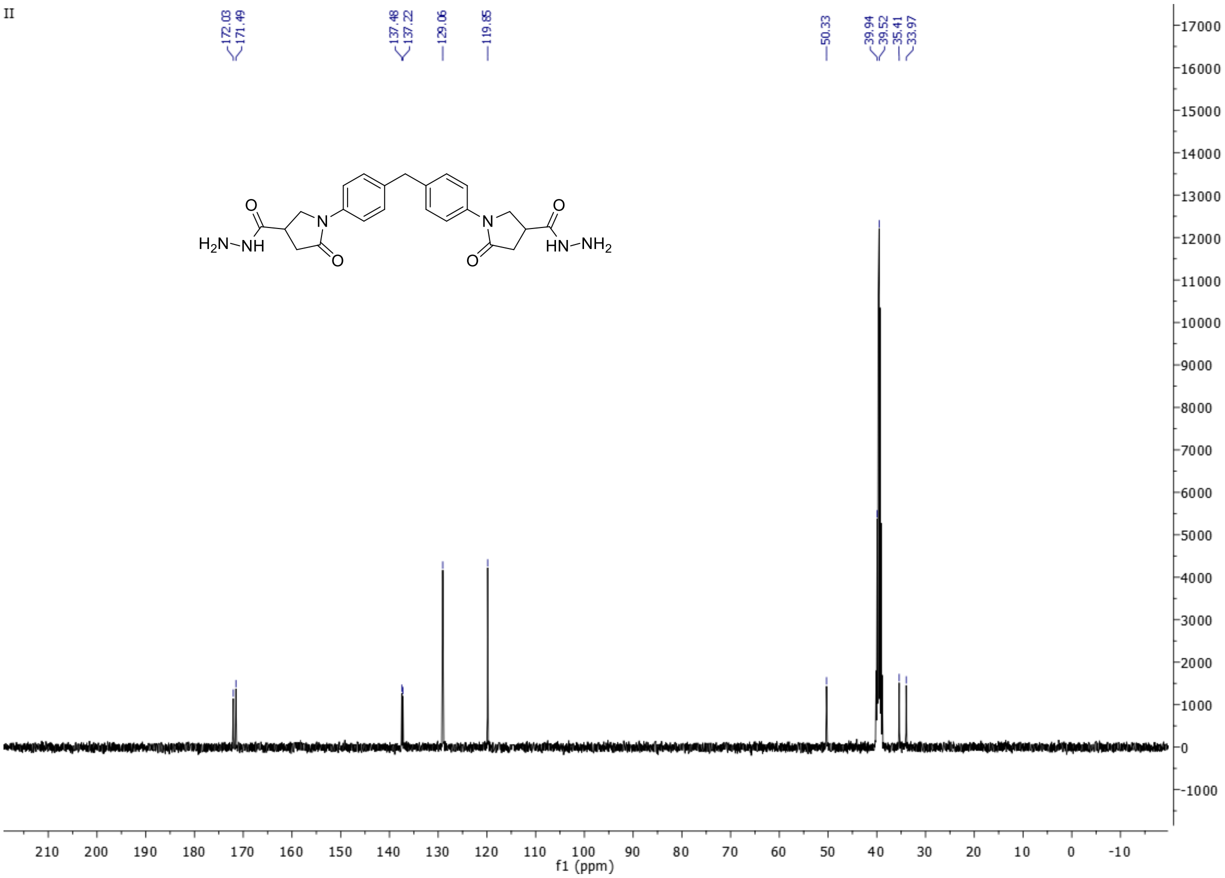

Figure S53. ^{13}C NMR (101 MHz, DMSO-d$_6$) spectrum of **34**

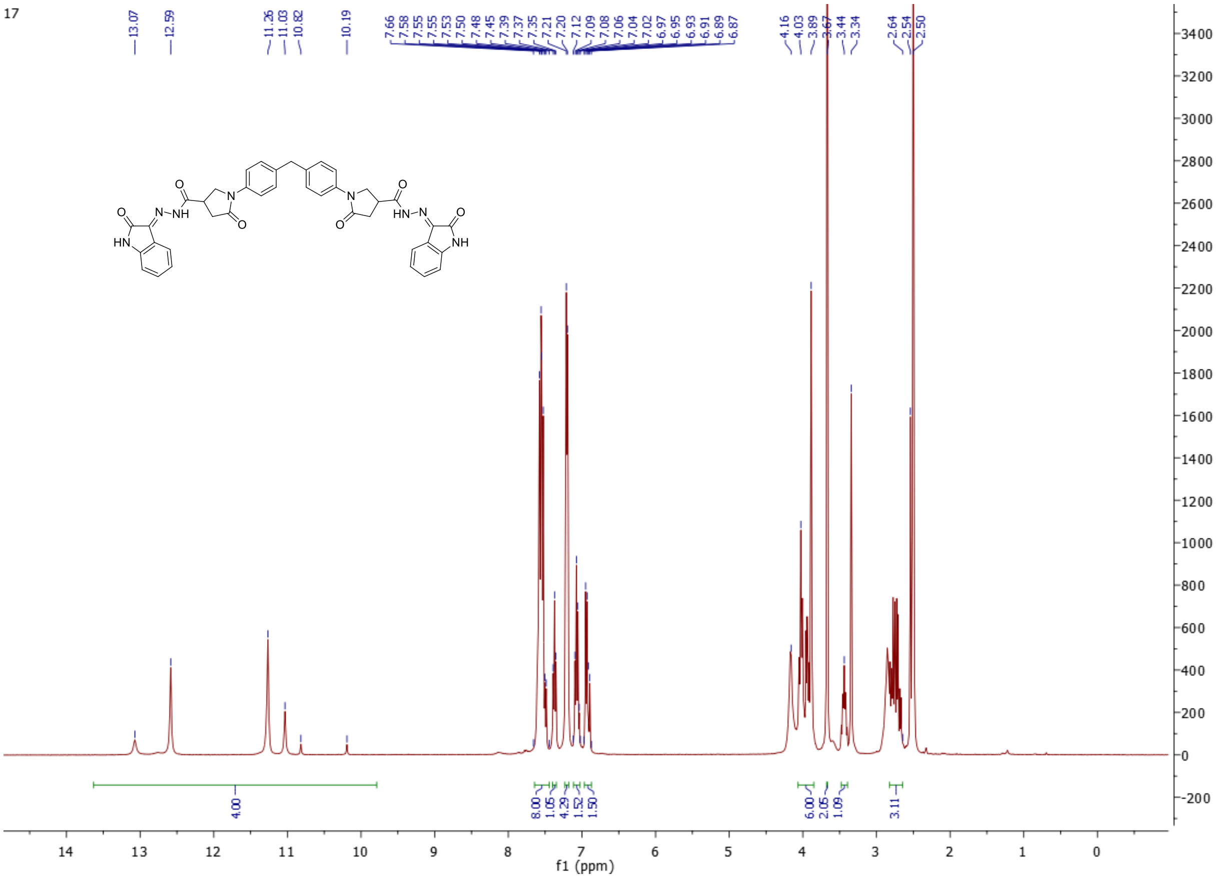

Figure S54 . ^{1}H NMR (400 MHz, DMSO-d_6) spectrum of **35**

17

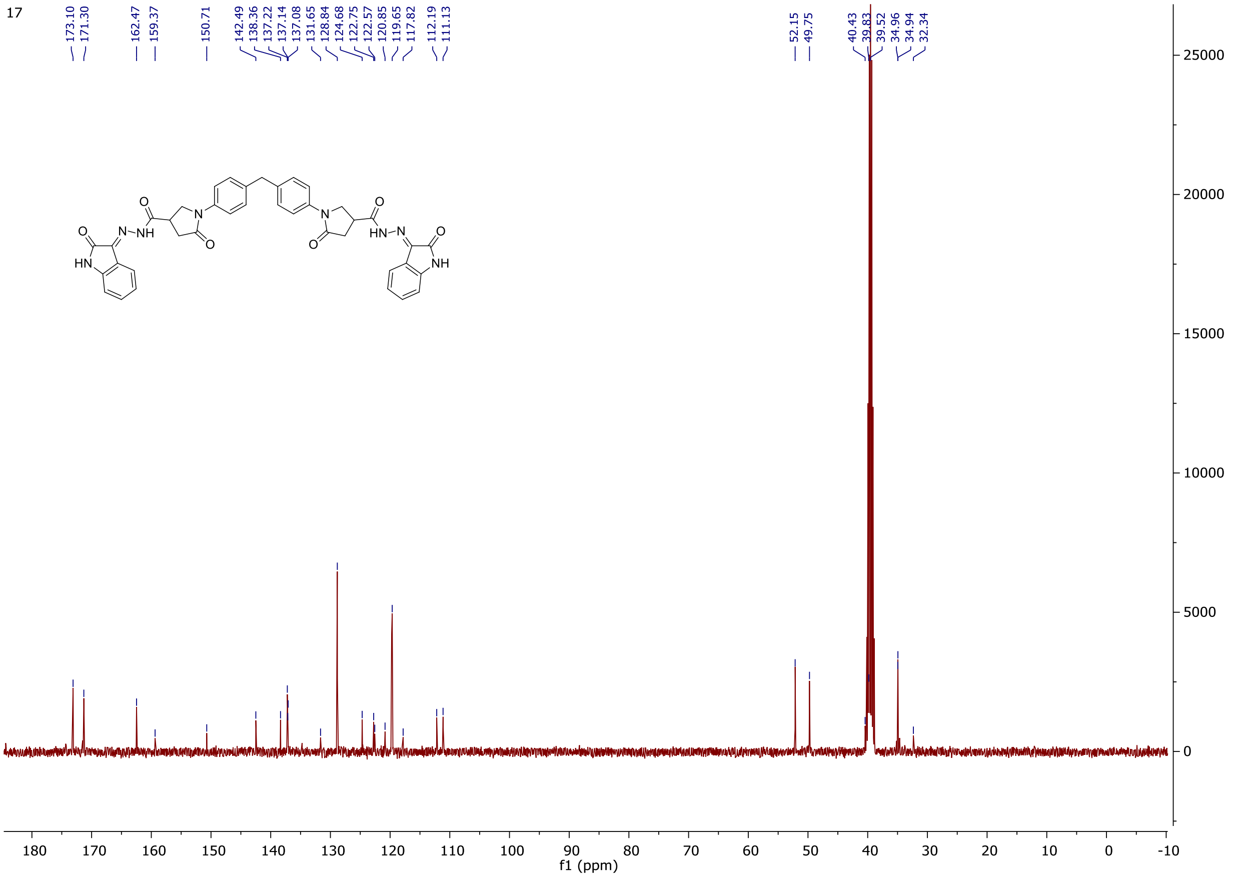

Figure S55. ^{13}C NMR (101 MHz, DMSO-d_6) spectrum of **35**

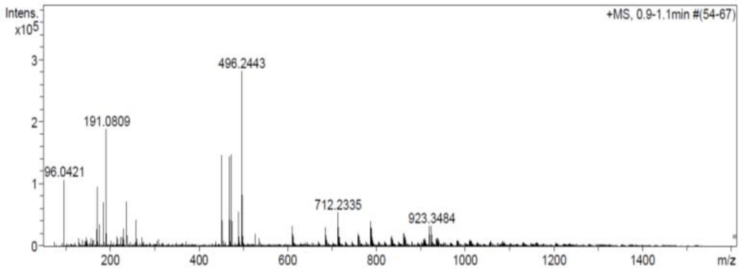

Meas. m/z	#	Formula	Score	m/z	err [ppm]	Mean err [ppm]	mSigma	rdb	e⁻ Conf	N-Rule
258.1210	1	C 10 H 12 N 9	100.00	258.1210	-0.0	1.5	8.9	9.5	even	ok
	2	C 9 H 16 N 5 O 4	40.29	258.1197	-5.2	-3.8	20.1	4.5	even	ok
708.5092	1	C 46 H 66 N 3 O 3	100.00	708.5099	0.9	1.0	41.8	15.5	even	ok
712.2335	1	C 47 H 30 N 5 O 3	100.00	712.2343	1.1	7.5	253.3	35.5	even	ok
	2	C 46 H 34 N O 7	74.60	712.2330	-0.8	5.7	257.4	30.5	even	ok
	3	C 43 H 26 N 11 O	14.18	712.2316	-2.6	3.7	265.1	36.5	even	ok
	4	C 34 H 38 N 3 O 14	0.52	712.2348	1.8	7.9	297.0	17.5	even	ok
	5	C 32 H 26 N 17 O 4	0.39	712.2348	1.8	8.1	299.4	28.5	even	ok
	6	C 31 H 30 N 13 O 8	0.36	712.2335	-0.0	6.3	305.3	23.5	even	ok
	7	C 30 H 34 N 9 O 12	0.13	712.2321	-1.9	4.1	308.1	18.5	even	ok
	8	C 28 H 22 N 23 O 2	0.09	712.2321	-1.9	4.3	310.4	29.5	even	ok
	9	C 19 H 34 N 15 O 15	0.00	712.2353	2.6	7.7	344.2	10.5	even	ok
	10	C 21 H 46 N O 25	0.00	712.2353	2.6	7.9	345.6	-0.5	even	ok

Figure S56. HRMS spectrum of **35**

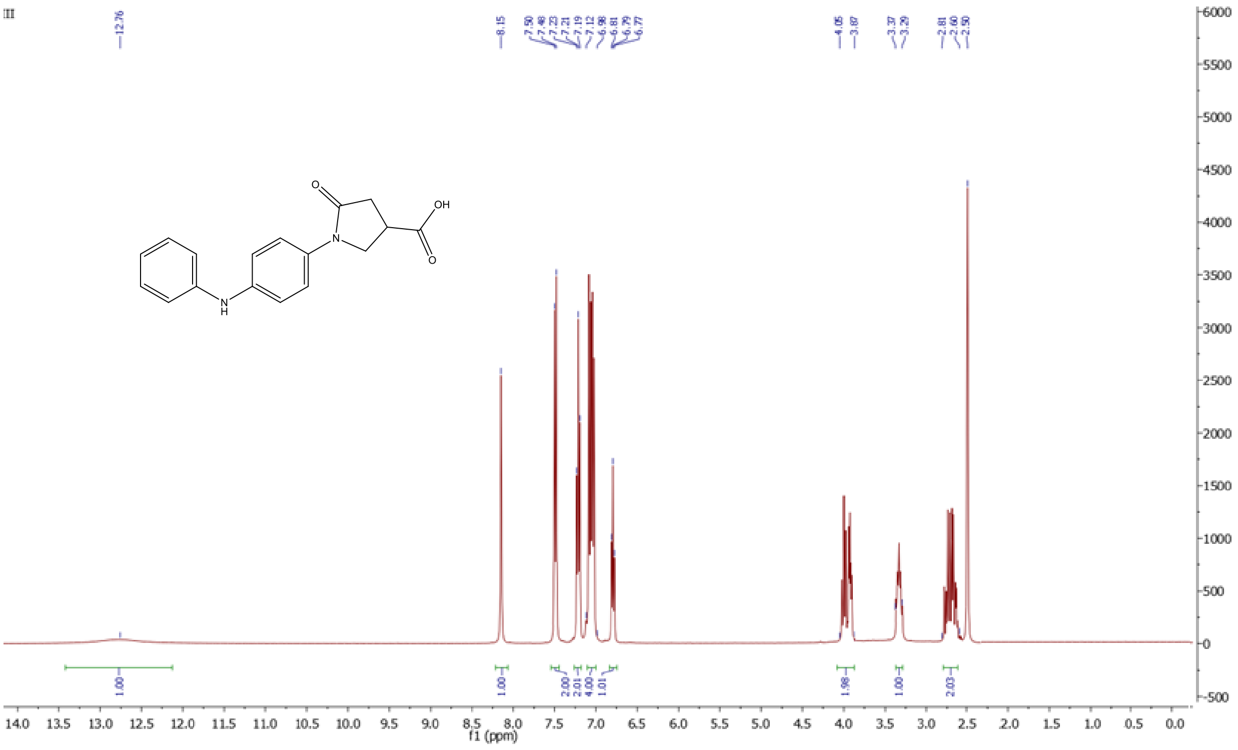

Figure S57. ^{1}H NMR (400 MHz, DMSO-d_6) spectrum of **36**

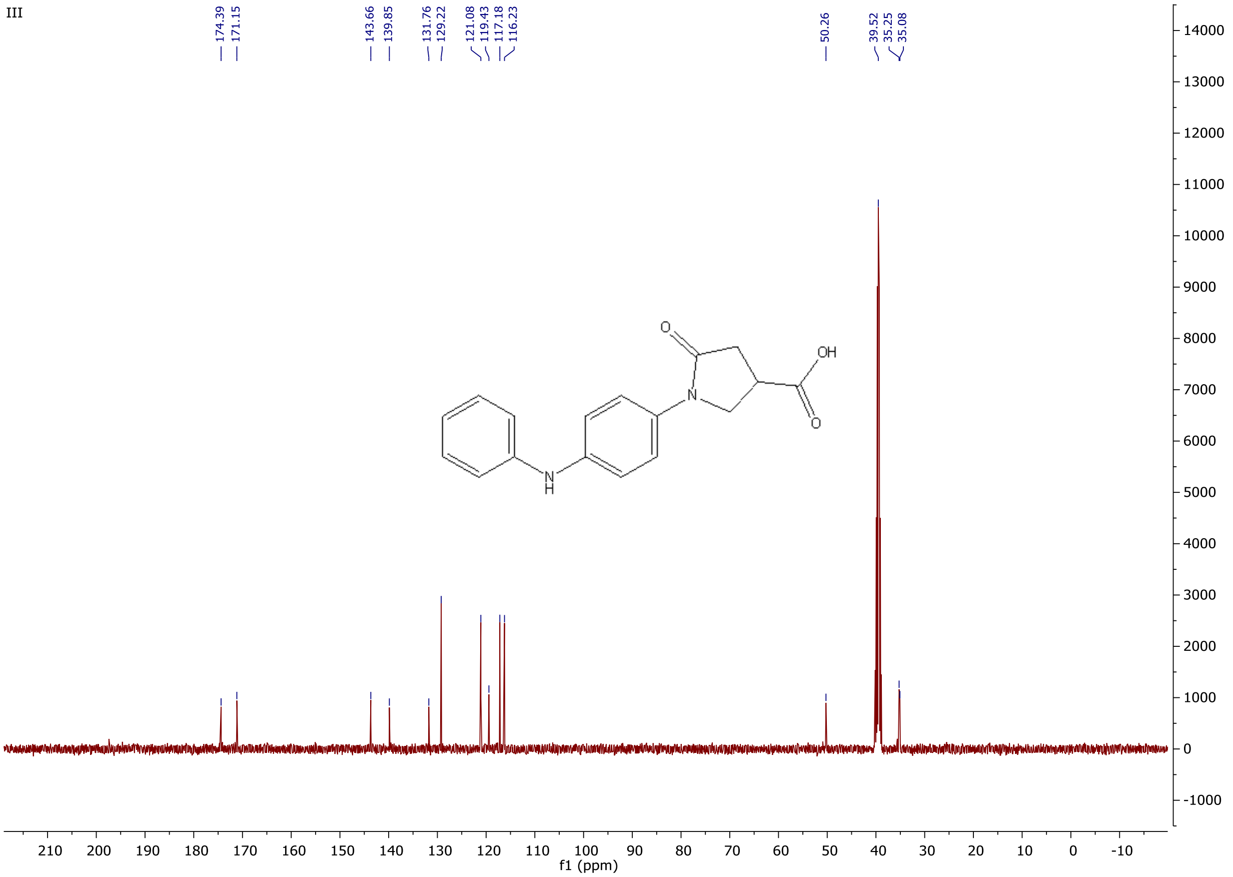

Figure S58. ^{13}C NMR (101 MHz, DMSO-d_6) spectrum of **36**

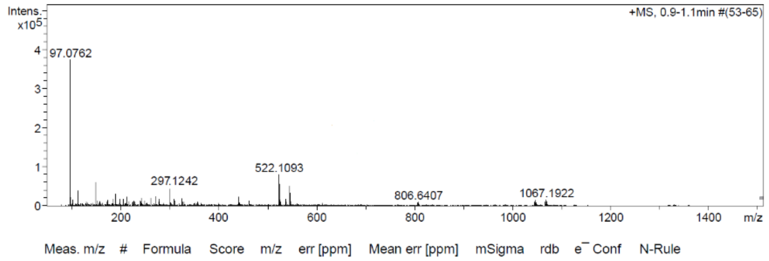

Figure S59. HRMS spectrum of **36**

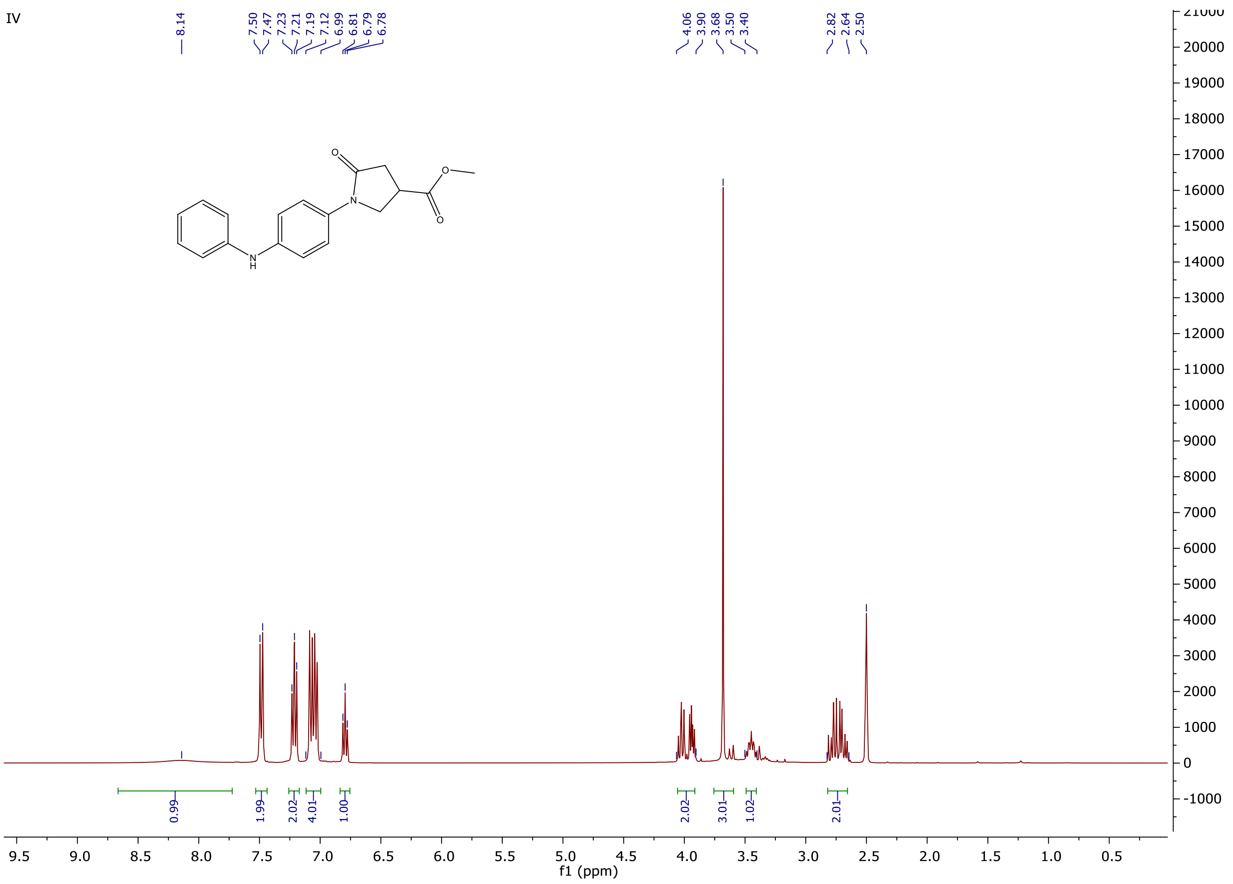

Figure S60. ^{1}H NMR (400 MHz, DMSO-d_6) spectrum of **37**

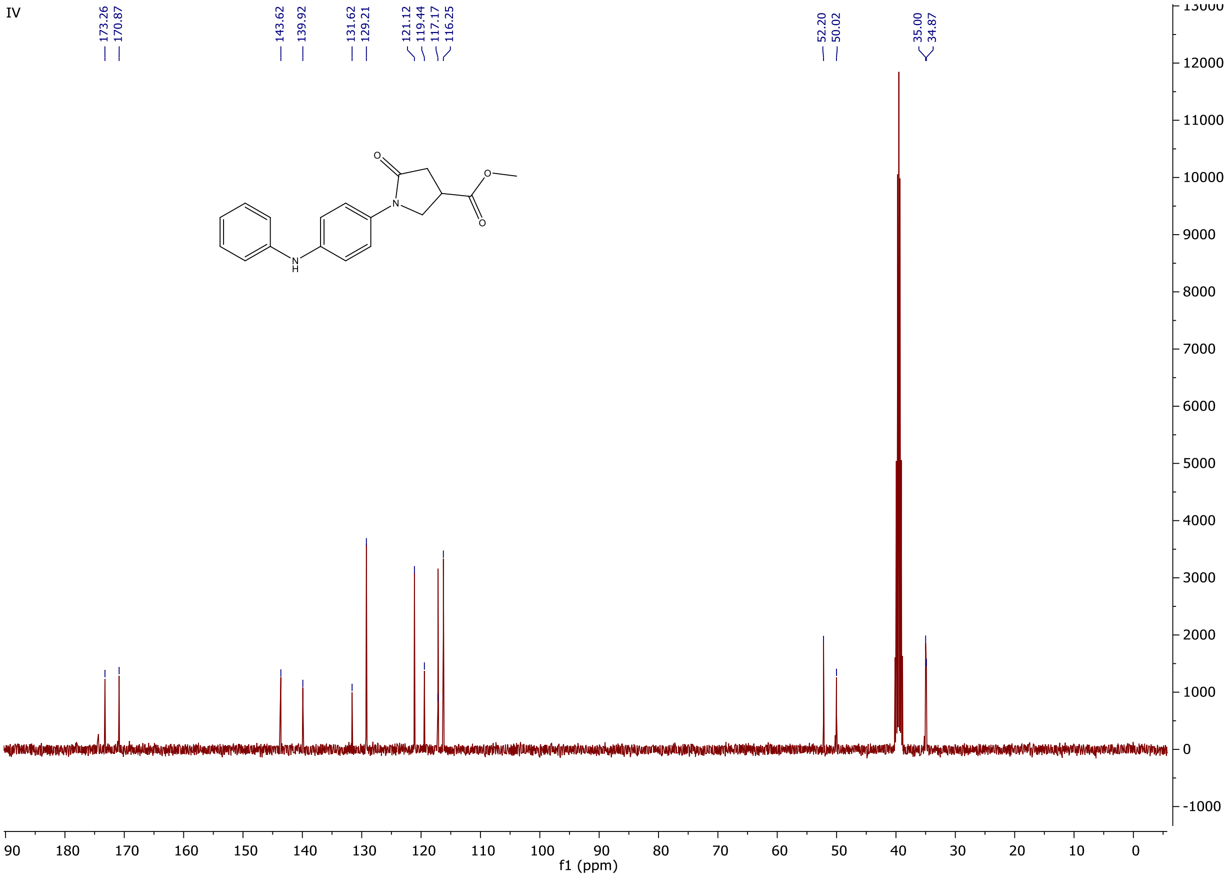

Figure S61. ^{13}C NMR (101 MHz, DMSO-d_6) spectrum of **37**

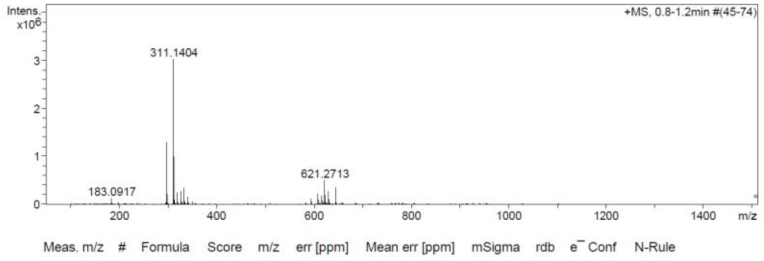

Figure S62. HRMS spectrum of **37**

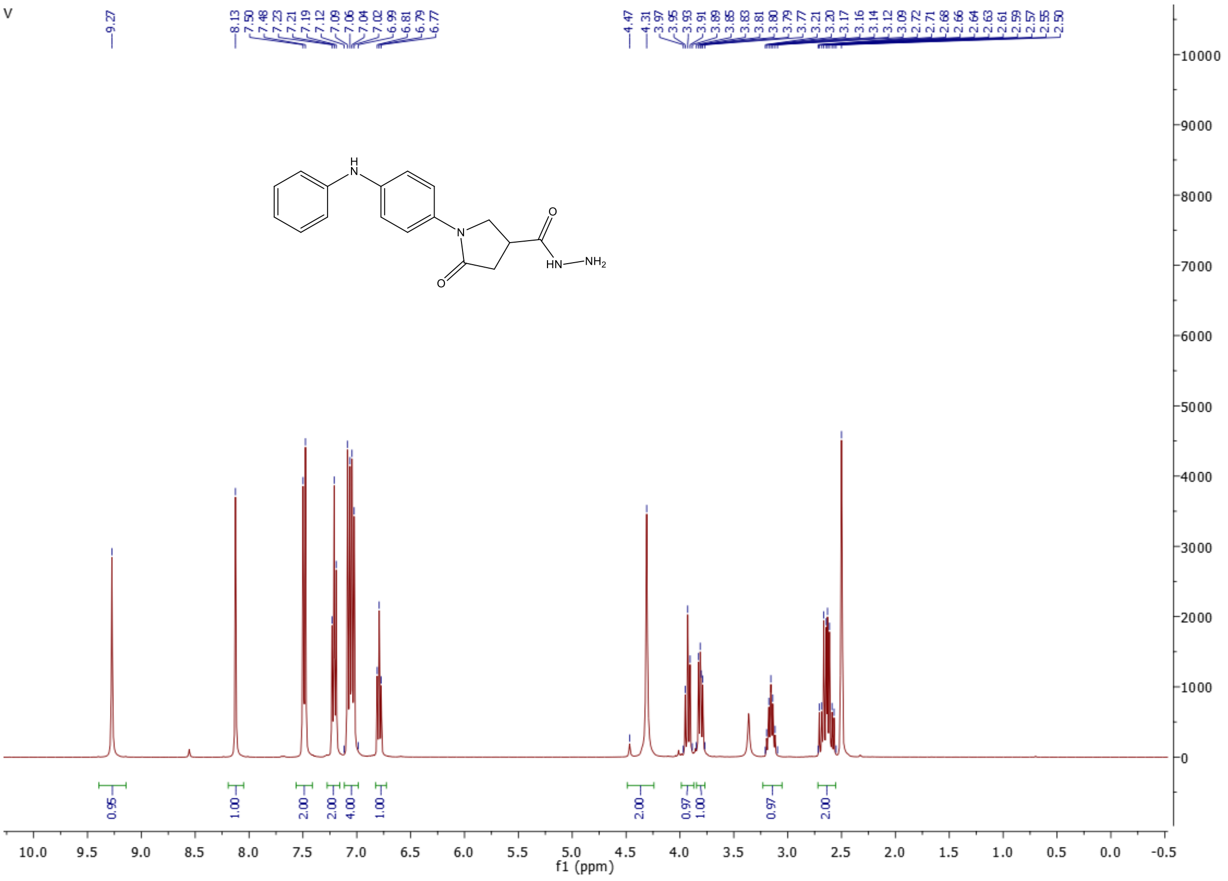

Figure S63. ^{1}H NMR (400 MHz, DMSO-*d*6) spectrum of **38**

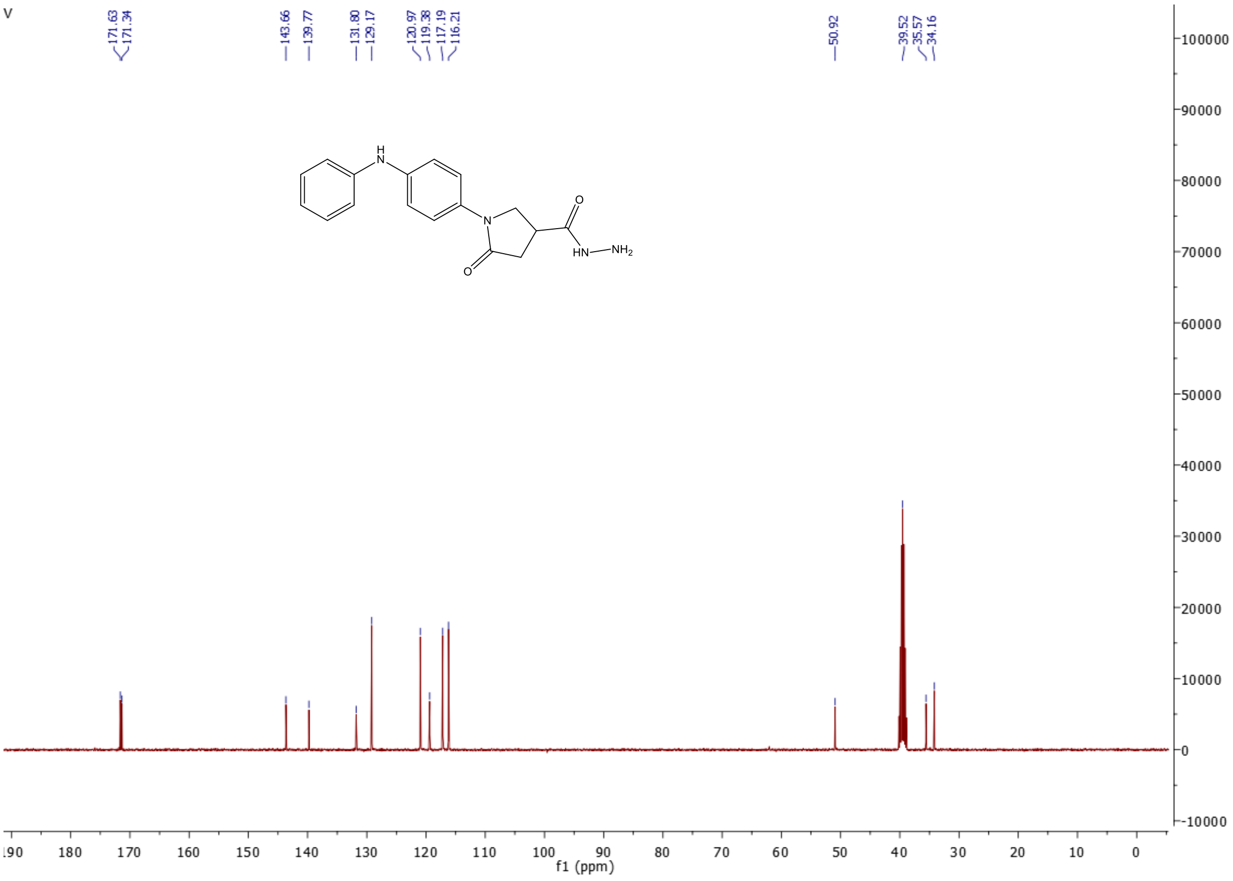

Figure S64. ^{13}C NMR (101 MHz, DMSO-d_6) spectrum of **38**

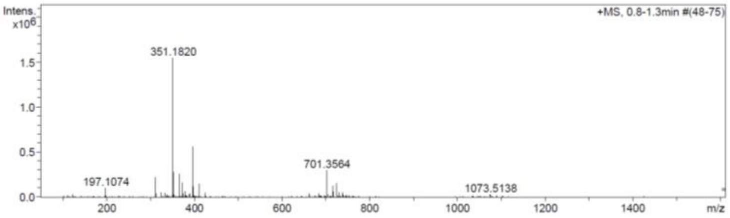

Meas. m/z	#	Formula	Score	m/z	err [ppm]	Mean err [ppm]	mSigma	rdb	e⁻ Conf	N-Rule
311.1498	1	C 15 H 17 N 7 O	87.43	311.1489	-2.7	-2.5	3.8	11.0	odd	ok
	2	C 17 H 19 N 4 O 2	100.00	311.1503	1.6	1.9	6.7	10.5	even	ok
	3	C 16 H 23 O 6	80.99	311.1489	-2.7	-2.4	8.3	5.5	even	ok
	4	C 19 H 21 N O 3	39.65	311.1516	5.9	6.2	13.0	10.0	odd	ok
351.1820	1	C 19 H 27 O 6	61.77	351.1802	-5.0	-4.8	16.6	6.5	even	ok
	2	C 18 H 21 N 7 O	52.65	351.1802	-5.1	-4.8	23.9	12.0	odd	ok
	3	C 20 H 23 N 4 O 2	100.00	351.1816	-1.2	-1.0	30.4	11.5	even	ok
	4	C 22 H 25 N O 3	66.45	351.1829	2.6	2.9	36.8	11.0	odd	ok
396.2398	1	C 21 H 34 N O 6	59.24	396.2381	-4.3	-3.9	15.1	5.5	even	ok
	2	C 20 H 28 N 8 O	53.09	396.2381	-4.3	-4.0	20.3	11.0	odd	ok
	3	C 22 H 30 N 5 O 2	100.00	396.2394	-0.9	-0.6	26.7	10.5	even	ok
	4	C 24 H 32 N 2 O 3	59.10	396.2407	2.5	2.8	35.4	10.0	odd	ok
701.3564	1	C 39 H 49 N 4 O 8	49.14	701.3545	-2.7	-2.3	6.2	17.5	even	ok
	2	C 41 H 51 N O 9	100.00	701.3558	-0.8	-0.4	12.7	17.0	odd	ok
	3	C 38 H 43 N 11 O 3	43.32	701.3545	-2.7	-2.4	12.8	23.0	odd	ok

Figure S65. HRMS spectrum of **38**

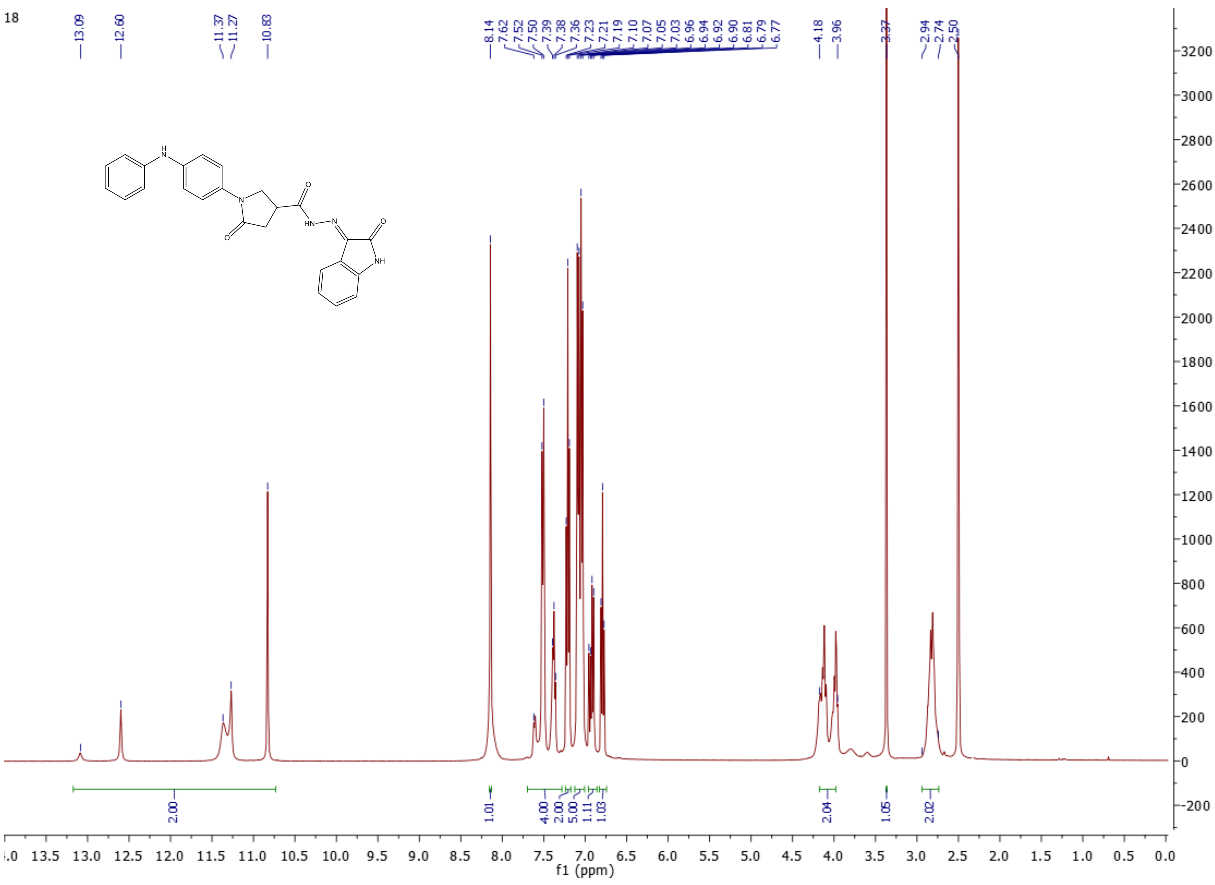

Figure S66. ^{1}H NMR (400 MHz, DMSO-d_6) spectrum of **39**

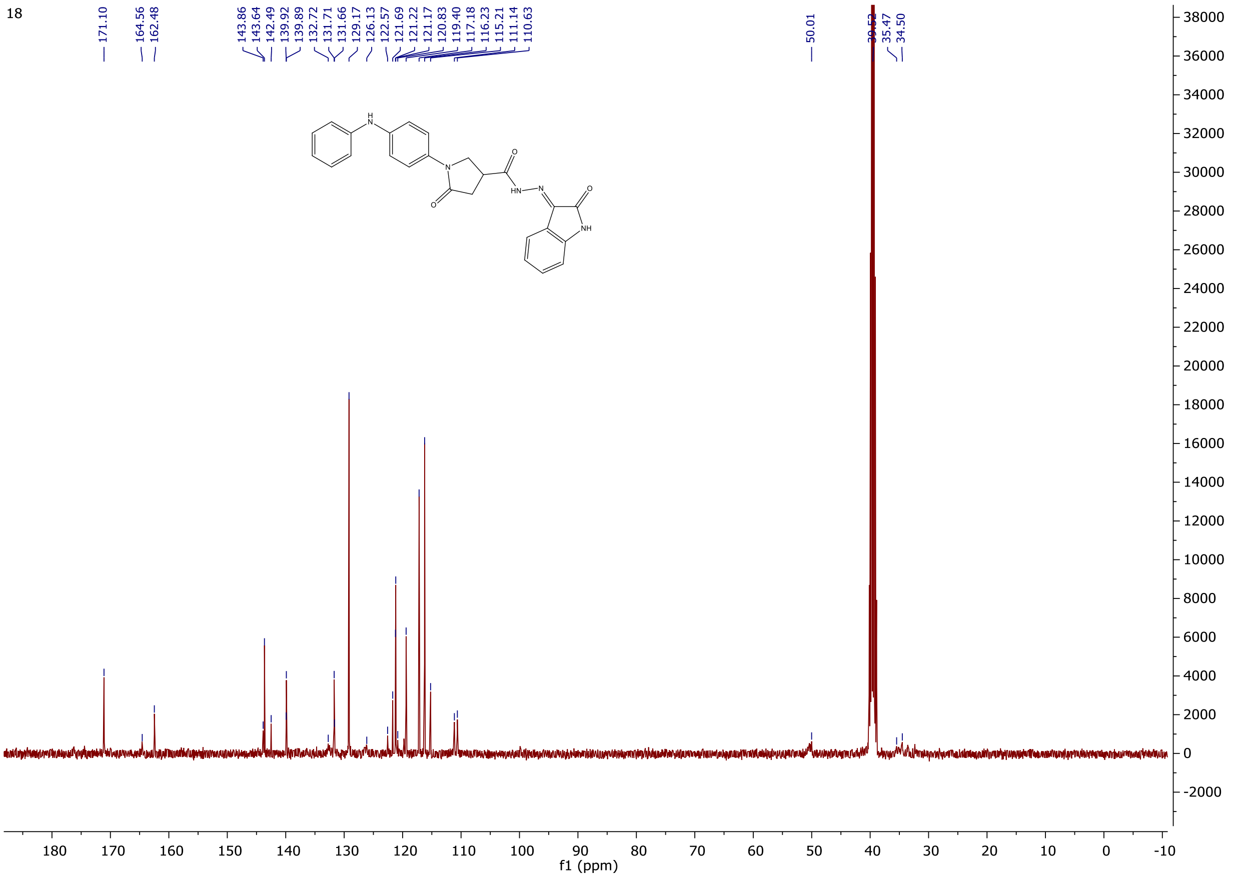

Figure S67. ^{13}C NMR (400 MHz, DMSO-d_6) spectrum of **39**

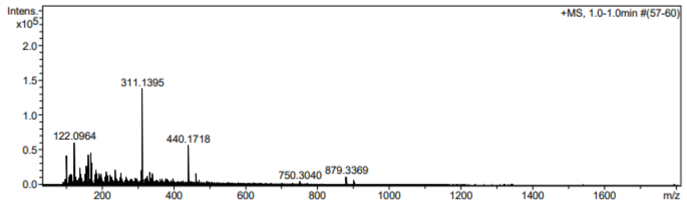

Meas. m/z	#	Formula	Score	m/z	err [ppm]	Mean err [ppm]	mSigma	rdb	e⁻ Conf	N-Rule
311.1395	1	C 18 H 19 N 2 O 3	100.00	311.1390	-1.5	-1.4	14.8	10.5	even	ok
440.1718	1	C 25 H 22 N 5 O 3	100.00	440.1717	-0.3	-0.3	24.9	17.5	even	ok
	2	C 24 H 26 N O 7	39.82	440.1704	-3.3	-3.3	33.5	12.5	even	ok

Figure S68. HRMS spectrum of **39**
